# Interpretable survival prediction for colorectal cancer using deep learning

**DOI:** 10.1038/s41746-021-00427-2

**Published:** 2021-04-19

**Authors:** Ellery Wulczyn, David F. Steiner, Melissa Moran, Markus Plass, Robert Reihs, Fraser Tan, Isabelle Flament-Auvigne, Trissia Brown, Peter Regitnig, Po-Hsuan Cameron Chen, Narayan Hegde, Apaar Sadhwani, Robert MacDonald, Benny Ayalew, Greg S. Corrado, Lily H. Peng, Daniel Tse, Heimo Müller, Zhaoyang Xu, Yun Liu, Martin C. Stumpe, Kurt Zatloukal, Craig H. Mermel

**Affiliations:** 1 Google Health, Palo Alto, CA USA; 2 Medical University of Graz, Graz, Austria; 3 Google Health via Advanced Clinical, https://www.health.google; 4 Google Health, Tempus Labs Inc., Chicago, IL USA

**Keywords:** Colon cancer, Prognostic markers, Computer science

## Abstract

Deriving interpretable prognostic features from deep-learning-based prognostic histopathology models remains a challenge. In this study, we developed a deep learning system (DLS) for predicting disease-specific survival for stage II and III colorectal cancer using 3652 cases (27,300 slides). When evaluated on two validation datasets containing 1239 cases (9340 slides) and 738 cases (7140 slides), respectively, the DLS achieved a 5-year disease-specific survival AUC of 0.70 (95% CI: 0.66–0.73) and 0.69 (95% CI: 0.64–0.72), and added significant predictive value to a set of nine clinicopathologic features. To interpret the DLS, we explored the ability of different human-interpretable features to explain the variance in DLS scores. We observed that clinicopathologic features such as T-category, N-category, and grade explained a small fraction of the variance in DLS scores (*R*^2^ = 18% in both validation sets). Next, we generated human-interpretable histologic features by clustering embeddings from a deep-learning-based image-similarity model and showed that they explained the majority of the variance (*R*^2^ of 73–80%). Furthermore, the clustering-derived feature most strongly associated with high DLS scores was also highly prognostic in isolation. With a distinct visual appearance (poorly differentiated tumor cell clusters adjacent to adipose tissue), this feature was identified by annotators with 87.0–95.5% accuracy. Our approach can be used to explain predictions from a prognostic deep learning model and uncover potentially-novel prognostic features that can be reliably identified by people for future validation studies.

## Introduction

Understanding and characterizing a patient’s cancer in order to estimate prognosis is essential for treatment decisions. Cancer staging systems, such as TNM classification, were created to categorize patients into different groups with distinct outcomes^1^. However, even within a specific TNM stage, there is often substantial variability in patient outcomes. While additional data, such as clinical variables, histopathologic parameters, and molecular features can provide important information^2,3^, there remains a need for more precise patient risk stratification to improve patient management and disease outcomes. In recent years, there has been a surge of interest in developing machine learning methods to provide novel prognostic information that is not captured in current staging guidelines^4–8^. However, despite some existing efforts to understand machine-learned prognostic features, strategies to gain insights into such features remain limited. If the learned features can be reproducibly identified and demonstrated to have independent prognostic value, this could enable the discovery of potentially novel features as well as build the necessary trust for AI-supported decision-making in medicine.

A specific use case of the role of prognostication in guiding treatment decisions can be found with colorectal adenocarcinoma, which is the third-most commonly diagnosed cancer and second only to lung cancer in terms of cancer mortality^9^. For stage II patients, adjuvant chemotherapy can be beneficial following resection of the tumor for a small subset of patients, but identifying the high-risk patients most likely to benefit represents a clinical challenge as overtreatment can result in substantial adverse effects^10,11^. For patients with stage III disease, although adjuvant chemotherapy is generally the standard of care, prognostic information has important implications for therapy regimen and duration^12^. Known histoprognostic features such as tumor budding and lymphovascular invasion among others can provide useful information, but challenges in both sensitivity and inter-pathologist variability limit their utility^2,13–15^. Better risk stratification within stage II and stage III colorectal cancer, therefore, offers opportunities to improve therapy decisions and patient care.

Previous machine learning-based efforts to predict the clinical outcomes using histopathology samples have used one of two main approaches^16^. The first strategy focuses on the extraction of pre-defined morphologic features using custom tools such as CellProfiler^17,18^, followed by statistical or machine learning techniques to understand which of the pre-defined features are correlated with survival^5,7,8,19,20^. The second and more recent strategy involves the use of weakly supervised deep learning approaches to directly predict survival from WSIs^4,6,21,22^, thus eliminating reliance on pre-defined features but introducing additional challenges in regards to model explainability. While some weakly supervised studies have tried to visualize the morphological features learned by the models^21,23,24^, providing reproducible descriptions of such features and evaluating the extent to which they actually explain the model predictions remain as challenges. In this study, we first present a weakly supervised deep learning system (DLS) for predicting disease-specific survival (DSS) in colorectal cancer patients and then develop a method for generating human-interpretable histologic features that can both explain the DLS predictions and be used as independent prognostic features.

## Results

### Data cohorts

This study included two cohorts of colorectal cancer cases. The first cohort spanned the years from 1984 to 2007. It was randomly split into a development set of 3652 cases (which was further split into training and tuning sets, see “Methods”) and a held-out validation set of 1239 cases (validation set 1). The second cohort of 738 colorectal cancer cases from 2008 to 2013 served as a second held-out validation set (validation set 2) to evaluate temporal generalization of the model to a more recent cohort (Table 1, Supplementary Fig. 1). Patient characteristics of the two validation sets are reported in Supplementary Table 1.

**Table 1:** Data used in this study.

Study	No. of cases				No. of DSS events (%)				No. of slides			
	Train	Tune	Validation set 1	Validation set 2	Train	Tune	Validation set 1	Validation set 2	Train	Tune	Validation set 1	Validation set 2
Stage II	1173	586	601	328	303 (26%)	152 (26%)	152 (25%)	80 (24%)	8687	4205	4452	3227
Stage III	1266	627	638	410	609 (48%)	294 (47%)	312 (49%)	183 (45%)	9617	4791	4888	3913
Stage II/III	2439	1213	1239	738	912 (37%)	446 (37%)	464 (37%)	263 (36%)	18304	8996	9340	7140

All cases were from the Institute of Pathology and the Biobank at the Medical University of Graz. Cases between 1984 and 2007 were randomly split in a ratio of 3:1 into a development set and validation set 1. The development set was further split into train and tune sets in a 2:1 ratio. Additional cases from 2008 to 2013 were obtained after model development as validation set 2. Disease-specific survival (DSS) was inferred from the International Classification of Diseases (ICD) code available for the cause of death. Only slides containing colorectal tissue were used for development and validation.

### Tumor segmentation model

We first developed a tumor segmentation model for the purpose of categorizing every region on a whole-slide image as tumor or non-tumor. This model was developed using pixel-level annotations provided for a subset of slides from the overall training split (Supplementary Fig. 1) and was evaluated on a held-out set of slides, also from the overall training split (44 slides, 6,866,573 patches, Supplementary Figs. 2–4). For classifying individual image patches as tumor vs. non-tumor, this model achieved an area under the receiver operating characteristic curve (AUC) of 0.985 (95% CI: 0.984–0.985). Using this model to identify regions of interest for the prognostic model instead of a simple tissue detector substantially improved the performance of the prognostic model (Methods, Supplementary Fig. 5).

### Evaluating DLS performance

The regions identified by the tumor segmentation model were used as the input for a second, prognostic model to produce case-level risk scores. The tumor segmentation model and prognostic model were applied sequentially to predict prognosis for each case, and are collectively referred to as the DLS.

We evaluated the ability of the DLS to predict DSS in two separate held-out validation sets (each comprising cases from different time periods). Validation set 1 had 10–35 years of follow-up, while the cases in the more recent validation set 2 had 5–9 years of follow-up. Thus, to allow direct comparisons across the two validation sets, we used the AUC for 5-year DSS, which is not affected by the differences in follow-up period available for the two validation sets. For stage II cases, the DLS demonstrated a 5-year AUC of 0.680 in the validation set 1 and 0.663 in the validation set 2 (Table 2). The 5-year AUC for stage III cases was 0.655 in both validation sets. In the combined cohorts of stage II and stage III cases, the 5-year AUC was 0.698 and 0.686 for the two validation sets, respectively. The 95% confidence intervals (CIs) are provided in Table 2.

**Table 2:** The 5-year AUC for disease-specific survival (DSS) prediction.

Cancer stage	Dataset	DLS	Quantitation of tumor-adipose feature
Stage II	Validation set 1 (*n* = 601 cases)	0.680 [0.631, 0.739]	0.645 [0.598, 0.700]
	Validation set 2 (*n* = 328 cases)	0.663 [0.592, 0.730]	0.634 [0.570, 0.697]
Stage III	Validation set 1 (*n* = 638 cases)	0.655 [0.617, 0.694]	0.629 [0.593, 0.680]
	Validation set 2 (*n* = 410 cases)	0.655 [0.600, 0.707]	0.682 [0.638, 0.743]
Stage II/III	Validation set 1 (*n* = 1239 cases)	0.698 [0.660, 0.729]	0.661 [0.629, 0.694]
	Validation set 2 (*n* = 738 cases)	0.686 [0.638, 0.723]	0.682 [0.641, 0.734]

**Fig. 1:**
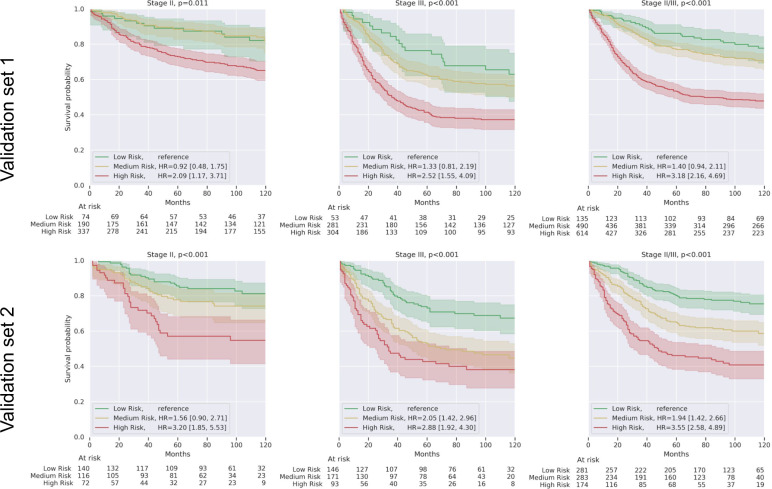
Kaplan–Meier curves on both validation sets for patients stratified by the prognostic deep learning system (DLS). Results are presented for stage II and stage III patients separately, and as a combined cohort (Stage II/III). High- and low-risk groups represent the highest and lowest risk quartiles from the tune set, respectively, based on the DLS prediction. Hazard ratios (HR) for the medium and high-risk groups are provided with the low-risk group as the reference group. Shaded areas represent 95% confidence intervals. *p* Values were calculated using the log-rank test comparing each high-risk group with the corresponding low-risk group.

In Kaplan–Meier analysis, the DLS demonstrated significant risk stratification in both validation sets (*p* < 0.001 for log-rank test comparing the high and low-risk DLS prediction quartiles; Fig. 1). The 5-year DSS rates of the high- and low-risk groups among stage II cases were 73% and 89%, respectively in the validation set 1. In validation set 2, the difference in survival rates between risk groups was similar with 5-year DSS of 57% (high risk) vs. 86% (low risk). For stage III cases, the survival rates for the high and low-risk groups were 41% versus 76% in the validation set 1 and 43% vs. 73% in the validation set 2. Similar results were observed for analysis over the combined cohort of stage II/III cases (Supplementary Table 2).

We further performed univariable and multivariable Cox regressions for both the DLS and clinicopathologic features (age, sex, tumor grade, and T, N, R, L, and V categories). The univariable analysis showed that the DLS was significantly associated with DSS for both stage II and stage III as well for the combined stage II/III cohort in both validation sets (*p* < 0.001; Supplementary Table 3). After adjusting for the clinicopathologic features, the DLS remained a significant predictor of DSS (*p* < 0.001; Table 3). We also compared the 5-year AUC of the Cox models containing the clinicopathologic features to those that additionally incorporated the DLS-assigned risk score (Supplementary Table 4A). For stage II, the addition of the DLS to the clinicopathologic features increased 5-year AUC over the clinicopathologic features alone by 0.120 and 0.085 for the two validation sets. For stage III, the corresponding increase over the clinicopathologic features alone was 0.065 (validation set 1) and 0.022 (validation set 2). For the combined stage II/III cases, the absolute increases were 0.055 and 0.038 with final AUCs of 0.733 and 0.721, respectively. The increases in prognostic value provided by the addition of the DLS were also observed based on c-index analysis (Supplementary Table 5). Finally, to more directly address the possibility of DLS correlation with depth of tumor invasion, we performed subanalysis on the T3 cases only. The performance of the DLS remained similar for this T3 subanalysis (Supplementary Table 6A).

**Table 3:** Multivariable Cox regression on the validation sets.

Variable		Stage II		Stage III		Stage II/III	
		Validation set 1	Validation set 2	Validation set 1	Validation set 2	Validation set 1	Validation set 2
DLS		**1.64 [1.39, 1.93], <0.001**	**1.54 [1.22, 1.94], <0.001**	**1.42 [1.26, 1.61]**, <**0.001**	**1.39 [1.20, 1.61], <0.001**	**1.54 [1.38, 1.70], <0.001**	**1.42 [1.25, 1.61], <0.001**
Age		1.13 [0.96, 1.33], 0.128	**1.49 [1.17, 1.89], <0.001**	**1.15 [1.05, 1.26], 0.004**	**1.25 [1.10, 1.43], <0.001**	**1.14 [1.05, 1.24], 0.002**	**1.31 [1.17, 1.47], <0.001**
Sex	Male	1.0 (reference)					
	Female	**0.69 [0.49, 0.96], 0.028**	0.75 [0.47, 1.21], 0.240	**0.76 [0.60, 0.95], 0.017**	0.96 [0.71, 1.29], 0.766	**0.74 [0.61, 0.89], 0.002**	0.89 [0.70, 1.14], 0.360
Grade	G1	1.0 (reference)					
	G2	0.80 [0.39, 1.66], 0.550	1.38 [0.49, 3.88], 0.536	1.16 [0.51, 2.64], 0.719	2.80 [0.87, 8.99], 0.083	0.96 [0.56, 1.66], 0.897	1.98 [0.92, 4.25], 0.082
	G3	0.88 [0.40, 1.96], 0.756	0.99 [0.33, 3.00], 0.990	1.47 [0.63, 3.39], 0.372	2.88 [0.89, 9.28], 0.077	1.19 [0.68, 2.08], 0.550	1.86 [0.85, 4.07], 0.119
	GX	0.92 [0.19, 4.36], 0.916	1.37 [0.25, 7.61], 0.718	0.73 [0.15, 3.68], 0.707	2.56 [0.60, 10.93], 0.204	0.78 [0.25, 2.37], 0.657	1.86 [0.65, 5.36], 0.250
Lymphatic invasion	L0	1.0 (reference)					
	L1	1.40 [0.88, 2.24], 0.154	0.74 [0.39, 1.41], 0.352	0.80 [0.60, 1.08], 0.146	0.99 [0.71, 1.38], 0.948	0.95 [0.74, 1.22], 0.692	0.92 [0.69, 1.23], 0.568
N-category	N0	N/A*				1.0 (reference)	
	N1	N/A*		1.0 (reference)		**1.89 [1.49, 2.39], <0.001**	**1.68 [1.23, 2.29], 0.001**
	N2	N/A*		1.10 [0.85, 1.42], 0.482	1.29 [0.95, 1.76], 0.107	**2.03 [1.55, 2.67], <0.001**	**2.21 [1.58, 3.08], <0.001**
	N3	N/A*		1.03 [0.73, 1.47], 0.858	0.60 [0.15, 2.46], 0.481	**1.85 [1.29, 2.66], 0.001**	1.02 [0.25, 4.18], 0.973
Margin status	R0	1.0 (reference)					
	R1	1.22 [0.44, 3.38], 0.700	1.01 [0.30, 3.39], 0.982	1.08 [0.66, 1.77], 0.761	0.74 [0.36, 1.54], 0.419	1.10 [0.71, 1.72], 0.666	0.81 [0.44, 1.50], 0.503
T-category	T1/T2	N/A*		1.0 (reference)			
	T3	1.0 (reference)		1.37 [0.88, 2.12], 0.159	**2.36 [1.09, 5.09], 0.029**	1.29 [0.84, 2.00], 0.244	**2.31 [1.07, 4.98], 0.032**
	T4	1.53 [0.95, 2.47], 0.081	**1.86 [1.08, 3.22], 0.026**	**1.66 [1.02, 2.71], 0.042**	**4.36 [1.98, 9.59], <0.001**	**1.66 [1.03, 2.65], 0.037**	**4.25 [1.95, 9.28], <0.001**
Venous invasion	V0	1.0 (reference)					
	V1	1.37 [0.67, 2.80], 0.389	1.42 [0.69, 2.95], 0.345	0.74 [0.48, 1.13], 0.165	1.18 [0.82, 1.69], 0.369	0.82 [0.57, 1.18], 0.278	1.20 [0.87, 1.65], 0.270

Numbers indicate hazard ratio followed by 95% confidence intervals in square brackets and *p* values (from a Wald test) after the comma. The corresponding univariable analysis is presented in Supplementary Table 3. Bold indicates statistically significant input variables (*p* < 0.05).

*N/A because stage II only contains N0 and T3 or T4 and stage III only contains N1 by definition (American Joint Committee on Cancer, AJCC).

### Understanding DLS predictions

Because the DLS was developed in a weakly supervised fashion without specifically being trained to predict known clinicopathologic features, we sought to understand what features were most highly associated with the DLS predictions. Specifically, we fit regression models to predict DLS scores using both the set of clinicopathologic features described above and a set of clustering-derived features (described below). Regression coefficients for individual features were used to evaluate the association between the DLS and individual features, while the adjusted coefficient of determination (*R*^2^) was used to measure the fraction of variance in DLS scores explained by each feature set.

### DLS association with clinicopathologic features

We first examined the association of the DLS with clinicopathologic features (Table 4). The features most significantly associated with the DLS risk score were the T and N categories. Specifically, cases with higher T and N categories also had higher DLS risk scores. Similar observations were made in a univariable correlation analysis (Supplementary Table 7A). Overall, the clinicopathologic features had an *R*^2^ of 0.18 (i.e., they explained only 18% of the variance in the DLS scores) in both validation sets, indicating that these clinicopathologic features leave a substantial proportion of the variance in DLS scores unexplained.

**Table 4:** Multivariable regression of case-level DLS score using clinicopathologic features as input.

Clinicopathologic feature	Validation Set 1			Validation Set 2		
	Coefficient	*p*	*R*^2^	Coefficient	*p*	*R*^2^
T3	**0.5454**	**<0.001**	0.18	0.1184	0.276	0.18
T4	**0.7775**	**<0.001**		**0.4032**	**<0.001**	
N1	**0.5496**	**<0.001**		**0.2912**	**<0.001**	
N2	**0.5942**	**<0.001**		**0.4752**	**<0.001**	
N3	**1.0311**	**<0.001**		0.3477	0.163	
R1	0.1108	0.427		**0.3365**	**0.011**	
L1	−0.1569	0.032		0.1063	0.074	
V1	0.2376	0.033		0.1332	0.054	
Grade 2	0.1032	0.467		0.0557	0.605	
Grade 3	**0.4342**	**0.004**		0.1800	0.112	
Grade X	0.5504	0.049		0.1968	0.287	
Sex (female)	−0.0091	0.862		0.0179	0.713	
Age at diagnosis	**−0.0670**	**0.002**		−0.0043	0.833	
Intercept	**−1.0471**	**<0.001**		**−1.4258**	**<0.001**	

For the overall model, *p* < 0.001 (*t* test). Each coefficient represents the relative increase of the DLS score associated with that variable. Bold indicates statistically significant input variables (*p* < 0.05).

### DLS association with clustering-derived features

Next, given the limited ability to exist clinicopathologic features to explain the variance in DLS scores, we generated a set of 200 human-interpretable histologic features by clustering embeddings from a deep-learning-based image-similarity model^25,26^. We then quantified the variance in DLS scores explained by the case-level quantitation of these clustering-derived features (as done above for clinicopathologic features). All 200 features combined demonstrated an *R*^2^ of 0.73 for the validation set 1 and an *R*^2^ of 0.80 for the validation set 2 (Table 5). A subset of ten of these features selected via forward stepwise selection achieved an *R*^2^ of 0.57 for the validation set 1 and an *R*^2^ of 0.61 for the validation set 2.

**Table 5:** Multivariable regression of case-level DLS score using clustering-derived features as input.

Feature #	Description	Validation Set 1			Validation Set 2		
		Coefficient	*p*	*R*^2^	Coefficient	*p*	*R*^2^
72	Small clusters of moderate to high-grade tumor cells intermixed with substantial adipose and a minor component of desmoplastic stroma	**0.2269**	**<0.001**	0.57	**0.2913**	**<0.001**	0.61
139	Low-intermediate grade tumor with predominant stroma of mature and intermediate desmoplasia	**0.1977**	**<0.001**		**0.1650**	**<0.001**	
23	Small clusters of high-grade tumor cells with predominant, mature desmoplasia and moderate TILs	**0.1096**	**<0.001**		**0.1931**	**<0.001**	
96	Small clusters of high-grade tumor cells, including single tumor cells, and a moderate amount of mature and intermediate desmoplasia	**0.1031**	**<0.001**		**0.1996**	**<0.001**	
146	Low-grade tumor with moderate differentiation and desmoplastic stroma with mature desmoplasia and occasional TILs	**−0.1248**	**<0.001**		**−0.2133**	**<0.001**	
122	Out of focus regions; predominantly low-grade tumor with tubule formation.	**−0.1323**	**<0.001**		−0.2867	0.187	
104	Low and Intermediate grade tumor with tubule formation and small, solid regions; Stroma with mature desmoplasia	**−0.1461**	**<0.001**		**−0.0505**	**<0.001**	
44	Intermediate grade tumor with irregular tubule formation; mature desmoplasia and focal areas of TILs	**−0.1510**	**<0.001**		**−0.1081**	**<0.001**	
101	Predominantly intermediate grade tumor with irregular tubule formation; minor component of mature, desmoplasia	**−0.2312**	**<0.001**		−0.0420	0.313	
144	Low-grade tumor with tubule formation and minor component of mixed stroma containing mature and intermediate desmoplasia with occasional, moderate TILs	**−0.3476**	**<0.001**		**−0.3794**	**<0.001**	
Intercept	N/A	**0.1256**	**0.002**		**0.1996**	**<0.001**	
Adding remaining 190 features		N/A	N/A	0.73	N/A	N/A	0.80

For the overall model, *p* < 0.001 (*t* test). Each coefficient represents the relative increase of the DLS score associated with that variable. Bold indicates statistically significant input variables (*p* < 0.05).

**Fig. 2:**
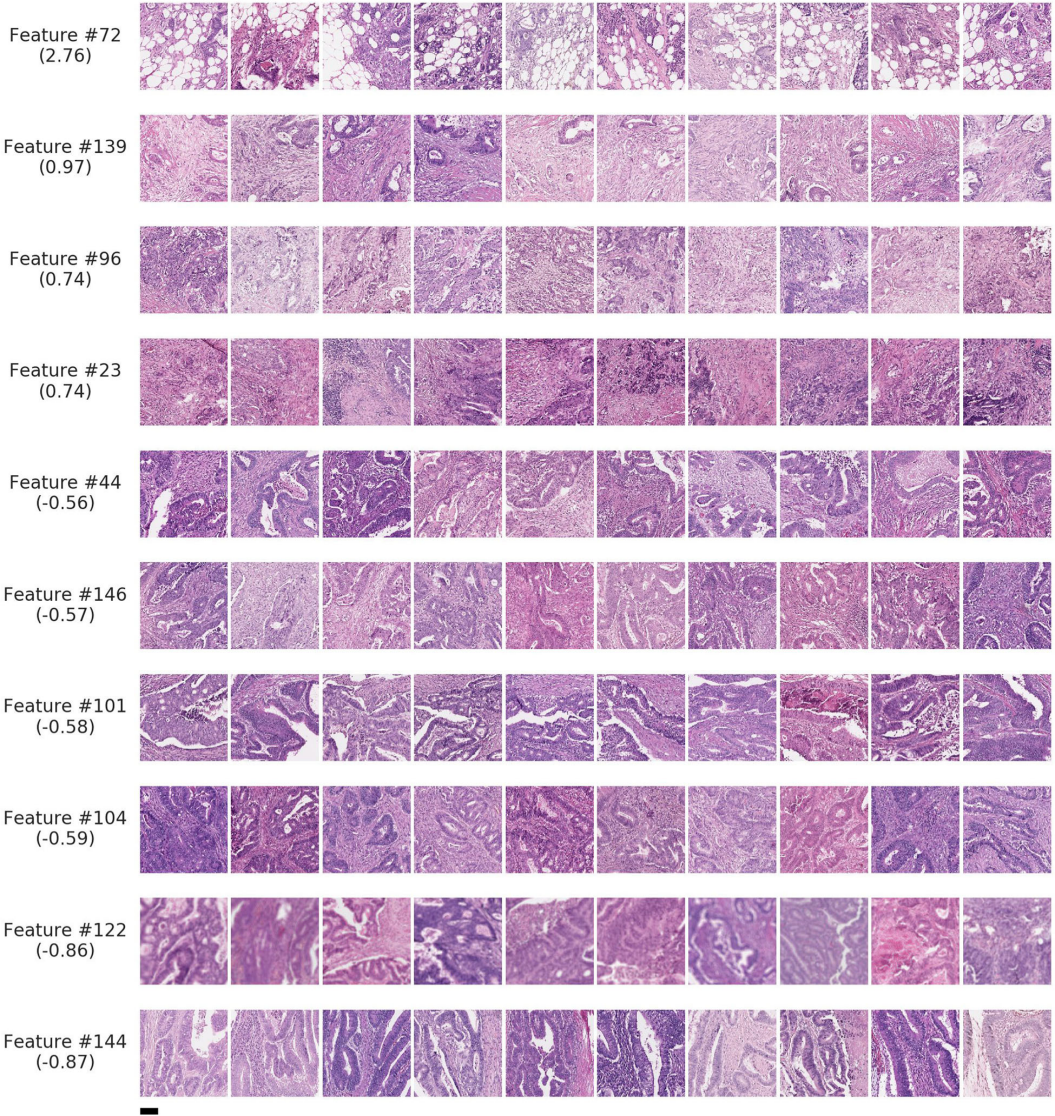
Representative patches for clustering-derived features associated with predictions of the deep learning system (DLS). Sample patches for a set of 10 clustering-derived features are shown. For each feature, the ten patches closest to the centroid were selected, after filtering to ensure they were from distinct cases (“Methods”). The case-level quantitation of these 4 high-risk and 6 low-risk features explains the majority of the variance in case-level DLS scores. Features are ranked according to the average DLS score, which is provided in parentheses. Scale bar indicates 0.1 mm.

**Fig. 3:**
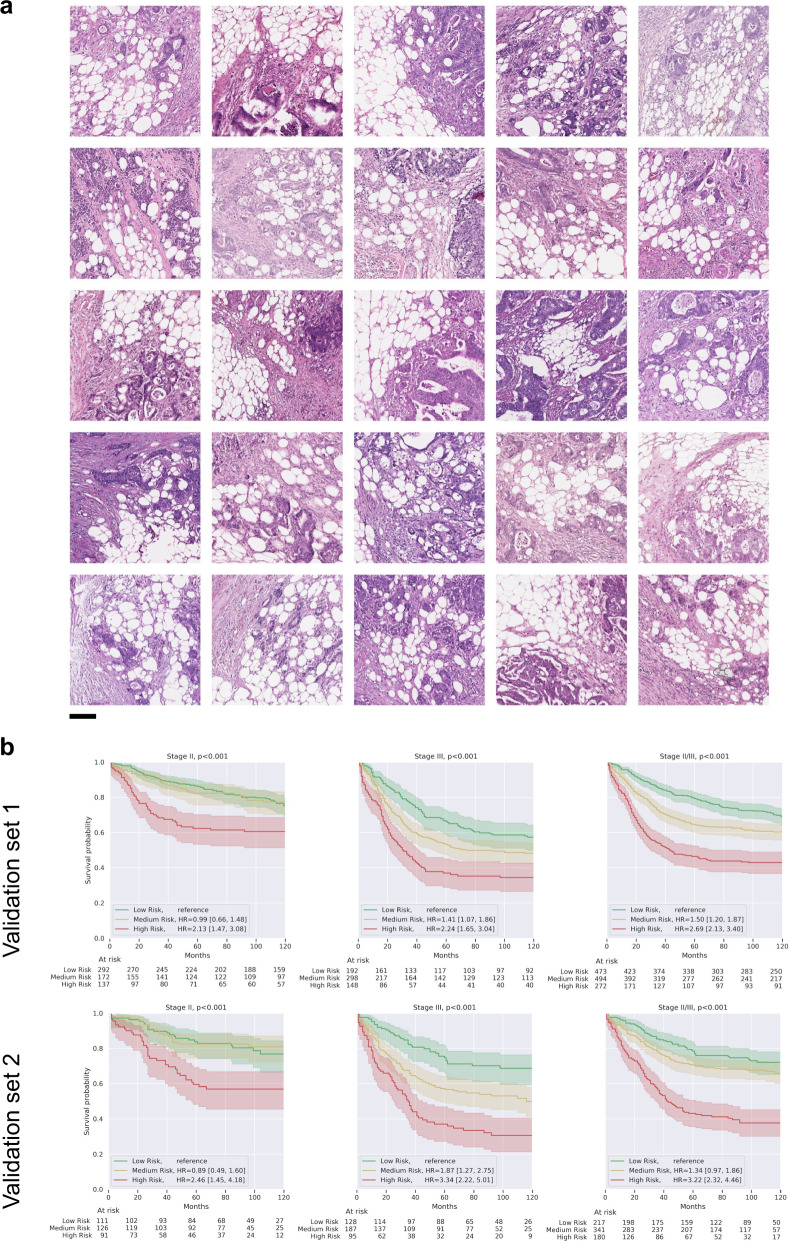
Visualizations and survival analysis of the clustering-derived feature with the highest DLS-predicted risk score (tumor-adipose feature, TAF). **a** Additional sample patches of the TAF cluster, each from a unique case. Scale bar indicates 0.1 mm. **b** Kaplan Meier curves on both validation sets for patients stratified by quantitation of TAF. These curves were generated following the same procedure as in Fig. 1. In stage II cases, the deviation in at-risk counts from the quartile marks for the low-risk and medium-risk groups are because many stage II cases did not contain any TAF.

For each of these top ten features, sample image patches exhibiting the feature (Fig. 2) were formally reviewed by three pathologists (Table 5). The feature with the highest regression coefficient was characterized by small, moderately-to-poorly differentiated tumor cell clusters adjacent to a substantial component of adipose tissue (cluster #72, Fig. 2, and Fig. 3a). In the remainder of this paper, we will reference this particular feature as the tumor-adipose feature (TAF). Another cluster with a high coefficient (cluster 139) was notable for predominant stroma consisting of intermediate and a mature desmoplastic reaction with a relatively small amount of low-to-intermediate grade tumor. In general, the features associated with higher risk DLS predictions involved intermediate to the high-grade tumor in small or solid clusters while the lower risk feature clusters typically contained lower grade tumor-forming glands and tubules and with high tumor to stroma ratio (Table 5, Figs. 2 and 3a). No remarkable findings were observed in regards to desmoplasia or tumor-infiltrating lymphocytes (TILs) across these ten feature clusters.

### DLS association with patch-level histoprognostic features

The analyses above were performed for case-level DLS scores and case-level quantitation of the clustering-derived features. To gain further insight into the DLS, we compared the average patch-level DLS score for a set of known histoprognostic features as well as the top ten clustering-derived features (Table 6 and Supplementary Fig. 6A). Known histoprognostic features were annotated by pathologists on a subset of validation set slides in order to provide patches for analysis (“Methods”). Among the known features, patches with lymphovascular invasion and perineural invasion had the highest average DLS scores (1.03 and 0.75, respectively), while patches from polyps had the lowest average score (−0.86). The TAF patches had the highest average score (2.76) both in the top 10 clusters and amongst all 200 clusters. This was also substantially higher than the other three high-risk features identified (#139, #96, and #23). The six features with negative average scores (relatively low risk), had scores ranging from −0.87 to −0.56. The relationship between the DLS score of each feature with the 5-year AUC for the quantitation of each feature is presented in Supplementary Fig. 6B.

**Table 6:** Average and interquartile range of DLS scores across patches for clustering-derived features and known histologic features.

Source of feature	Feature name	DLS score mean (95% CI)	DLS score interquartile range
Known features (manually annotated by pathologists; 87,325 patches across 161 slides)	Lymphovascular invasion	1.03 [0.33, 1.95]	[0.09, 1.82]
	Perineural invasion	0.75 [0.14, 1.28]	[−0.18, 1.68]
	Intratumoral budding	0.33 [0.00, 0.59]	[−0.63, 1.15]
	Peritumoral fibrosis	0.26 [0.02, 0.42]	[−0.73, 1.18]
	Peritumoral budding	0.10 [−0.10, 0.30]	[−0.96, 0.94]
	Other adenocarcinoma	−0.46 [−0.57, −0.36]	[−1.36, 0.25]
	Polyp	−0.86 [−1.26, −0.59]	[−1.57, -0.24]
Clustering-derived (from clusters identified by a deep learning-based visual similarity model; 2,568,691 patches across 9340 slides)	72	2.76 [2.59, 2.93]	[1.66, 3.74]
	139	0.97 [0.91, 1.02]	[0.40, 1.61]
	96	0.74 [0.69, 0.80]	[0.13, 1.42]
	23	0.74 [0.68, 0.78]	[0.08, 1.38]
	44	−0.56 [−0.61, −0.50]	[−1.14, −0.02]
	146	−0.57 [−0.62, −0.52]	[−1.19, 0.03]
	101	−0.58 [−0.62, −0.54]	[−1.13, −0.04]
	104	−0.59 [−0.64, −0.53]	[−1.18, −0.02]
	122	−0.86 [−1.04, −0.71]	[−1.34, −0.35]
	144	−0.87 [−0.91, −0.83]	[−1.38, −0.35]

Confidence intervals were computed via block bootstrapping.

### Tumor-adipose feature

The TAF finding was notable in several respects. First, across all clustering-derived features, TAF had the strongest association (R^2^) with the DLS scores and the highest patch-level DLS scores (2.76 vs. the next-highest at 0.97). Second, case-level TAF quantitation (Supplementary Fig. 7) was independently highly prognostic (Table 2, Fig. 3b, Supplementary Table 4B, Supplementary Table 6B, Supplementary Table 7B). Given these results, we evaluated whether it was possible for researchers and pathologists to accurately identify TAF, thus enabling future work to better understand its biological and prognostic significance. Briefly, three non-anatomic-pathologists and two anatomic pathologists were presented with a total of 200 image patches from tumor-containing regions. For each patch, participants were instructed to indicate if that patch contained TAF or not. Accuracies for the non-pathologists were 90.0%, 93.0%, and 95.5%, and accuracies for the pathologists were 87.0% and 90.5%. The interpathologist concordance was 93.5%.

## Discussion

In this study, we demonstrated the ability of a weakly supervised DLS to predict DSS in intermediate-stage colorectal cancer directly from unannotated, routine histopathology slides. We then developed a method for generating human-interpretable histologic features by clustering embeddings from a deep-learning-based image-similarity model. We used these clustering-derived features, which explained a large fraction of the variance in DLS predictions, to gain an understanding of the histologic features the DLS scored as high and low risk. We found that one particular clustering-derived feature, characterized by poorly differentiated tumor cell clusters adjacent to adipose tissue, was strongly associated with high DLS risk scores, independently associated with poor prognosis, and able to be reproducibly identified by pathologists.

We conducted a variety of statistical analyses that demonstrated the high prognostic performance of the DLS. First, the DLS provided significant risk stratification even within stage II and stage III cases. Furthermore, the difference in 5-year survival rates between high- and low-risk groups defined by the DLS was comparable to or greater than currently used prognostic factors such as obstruction, T-category, TIL, desmoplasia, lymphovascular invasion, and perineural invasion^11,27–31^. In multivariable analysis, the DLS added significant prognostic value to a set of nine clinicopathologic baseline features. These results held across two validation datasets, including a temporal validation set from a later time period. These findings represent a generalization of DLS performance, even to a cohort of cases with significant differences in baseline characteristics (Supplementary Table 1) as well as potential differences in treatment and technical aspects of tissue and slide preparation. Finally, the DLS performance was similar to that recently reported by Skrede et al.^4^ using a comparable weakly supervised approach, further validating that substantial risk stratification is achievable with this type of deep learning approach.

Given the demonstrated ability of the DLS to risk-stratify patients, there is a potential for the DLS to inform clinical decisions involving the use of adjuvant chemotherapy. Specifically, the DLS could help identify high-risk stage II patients most likely to benefit from therapy or inform decisions about therapy regimens for low-risk stage III patients in order to minimize overtreatment. Prospective studies to evaluate the impact of DLS-informed treatment decisions on patient outcomes are warranted, especially when combined with existing biomarkers that may provide complementary prognostic value.

Explainability is an important aspect of building the trust and transparency necessary for the adoption of such model-informed clinical decision-making. This is especially true for weakly supervised prognostic models which learn to associate histologic features in unannotated whole-slide histopathology images without any human supervision. Although some insights have been derived from characterizing saliency heatmaps or example patches with extreme risk scores^21^, researchers’ ability to systematically characterize the histologic features learned by their model and evaluate the extent to which these features actually explain the model predictions remains limited.

While prior work has described weakly-supervised prognostic models for colorectal cancer with comparable performance to our DLS^4^, an important advance offered by our study is the development of a computational method for generating human-interpretable “clustering-derived” features that can explain the DLS risk scores. We showed that while a set of nine clinicopathologic features explained only a small fraction of variance in DLS scores (less than 20%, Table 4), a set of 10 clustering-derived features, which could be understood, described, and reproducibly identified by pathologists, explained the majority of variance in DLS scores (about 60%, Table 5). Finally, the complete set of 200 features explained another 15–20% of the variance in the DLS. This means approximately 20% of the variance remained unexplained, suggesting some features remained unappreciated by our method and avenues for future work.

Although some of the features learned by weakly supervised prognostic models may be well-known, there is also the possibility of learning previously unappreciated prognostic features. The clustering-derived feature most strongly associated with high DLS risk scores and poor prognosis was notable for its distinctive histomorphological appearance, including moderately to poorly differentiated tumor cells in close proximity to adipocytes, thus termed “Tumor Adipose Feature” (TAF). One initial interpretation might be that this feature represents invasion into the subserosa (T3 of TNM staging) or beyond (T4), and thus that the model may have learned a representation of the T-category, which has known prognostic significance^1^. However, both the DLS prediction and TAF quantitation remain significantly associated with survival even within T3 cases (Supplementary Table 6), suggesting prognostic value independent of T-category.

A hypothesis that could explain the independent prognostic value of TAF is submucosal adipose tissue as a prognostic factor itself, potentially associated with inflammatory bowel disease or obesity^32,33^. In regards to obesity, there is some evidence to suggest that body-mass index, visceral fat, and subcutaneous fat may be associated with adverse outcomes in metastatic colorectal cancer^34^. More speculatively, this finding may be consistent with an adverse role for cancer-associated adipocytes in colorectal cancer, as has been described in other cancer types^35,36^. Finally, there are notable morphologic similarities between TAF and irregular tumor growth at the invasive edge, potentially representing an association with “infiltrative” vs. “pushing” configurations of the tumor border^37,38^. Finally, although the TAF is visually distinct, is highly associated with case-level DLS risk predictions, and represents the feature with the highest risk score, other clusters also appear independently prognostic. Further work is warranted to better understand the biological significance of TAF and other clustering-derived features.

Our study has some limitations. First, as a retrospective study, treatment pathways present an important confounding factor that is difficult to control for, including potential differences in neoadjuvant and adjuvant therapy. Though treatment guidelines within stage II and within stage III colorectal cancer cohorts are fairly uniform, at least some variability in treatment likely exists. Progression-free survival may be an endpoint that is less susceptible to treatment confounding but was unfortunately not available at the scale required for this study. Second, while the non-random temporal validation set demonstrates generalization in the face of significant changes in case characteristics over time (Supplementary Table 1), validation in geographically diverse cohorts would be needed to further support the generalization of the DLS to other cohorts containing complete, routine clinical cases. Unfortunately, such geographically diverse data with the necessary imaging and clinical data were not available for this study. A further limitation is that we were not able to evaluate the association between the DLS and several known prognosis factors such as tumor budding, the number of lymph nodes examined, tumor location, obstruction, microsatellite instability, TIL, molecular profile (e.g., BRAF and KRAS), desmoplasia, or histologic subtypes^11,30,31,39,40^. While obvious associations with TILs, desmoplasia, or subtype were not observed in our analysis of clustering-derived features, the association of the DLS scores with these factors will need to be examined in future work. Though used in our analysis, the lymphovascular invasion was not formally re-evaluated for the purposes of this study and thus may not be exhaustively recorded. While we were able to show that individual patches containing TAF can be reproducibly identified, suggesting that the feature is readily learnable, further work is required to validate the prognostic value of pathologists’ case-level quantitation of TAF. Doing so will require the development of guidelines to ensure consistent scoring across pathologists. While the use of a clustering algorithm facilitated the identification of TAF, the clusters themselves are based on image similarity rather than specific histopathological concepts. Thus, in building on the methods and findings here, pathologist-guided refinement of algorithm-derived feature clusters may lead to even more prognostic and well-defined features. Finally, the cluster analysis provided valuable insights into the features that could explain the variance in DLS scores, but there may be additional important features that were not identified by these specific clusters. For example, generating clusters using embeddings from different machine learning models^25^ could potentially help identify additional features that further explain DLS predictions.

To conclude, the present work demonstrates the application of deep learning methods to learn and describe histomorphologic features with prognostic value for colorectal cancer, without pre-specification of features. The prognostic predictions of the DLS provided significant risk stratification in both stage II and stage III cases, even after adjusting for a number of clinicopathologic features including T category, N category, and tumor grade. Individual histologic features associated with risk predictions by the DLS were also characterized, providing a framework for future efforts in explaining weakly supervised models in histopathology. Finally, this analysis enabled the description and reproducible identification of a visually distinctive machine-learned feature with independent prognostic significance. This ability to learn from machine learning represents an important first step in allowing experts to further study new concepts discovered using weakly supervised deep learning models.

## Methods

### Data cohorts

This study utilized archived formalin-fixed paraffin-embedded, hematoxylin and eosin-stained pathology slides from the Institute of Pathology and the BioBank at the Medical University of Graz^41^. Institutional Review Board approval for this retrospective study using de-identified slides was obtained from the Medical University of Graz^42,43^ (Protocol nos. 30–184 ex 17/18). All available slides in archived stage II and stage III colorectal cancer resection cases between 1984 and 2013 were retrieved, de-identified, and scanned using a Leica Aperio AT2 scanner at 20X magnification (0.5 μm/pixel). The complete set of digitized whole slide images (WSIs) consisted of 6,437 cases and 114,561 slides. Additional de-identified clinical and pathological information corresponding to each case was extracted from pathology reports^44,45^ along with data from Statistik Austria. This information included pathologic TNM staging, tumor grade (G), resection margin status (R), sex, and age at diagnosis. When indicated in the report, the presence of lymphatic invasion (L) and venous invasion (V) were also extracted. DSS was inferred from the International Classification of Diseases (ICD) code available for the cause of death and only colorectal cancer-associated ICD codes were considered disease-specific (C18, C19, C20, C21, C26, and C97), with other types of cancer excluded.

All 114,561 slides underwent manual review by pathologists to identify the stain and tissue type. Immunohistochemistry-stained slides and non-colorectal specimens such as lymph node, small intestine, and other tissue types, were excluded. In addition, cases with low tumor content, death within 30 days of surgical resection, and secondary tumor resections were also excluded, leaving 43,780 slides from 5629 cases (Supplementary Fig. 1). These slides were partitioned into two cohorts. All cases from 1984 to 2007 were assigned to the first cohort, which was randomly subdivided into a training set, a tuning set, and the first validation set in a 2:1:1 ratio. To further evaluate the performance of the model and assess temporal generalizability, all cases from 2008 to 2013 were assigned to the second validation set. This division of years was used to ensure 5 years of follow-up were available for all cases, and that validation set 2 contained an arbitrarily determined 5 years' worth of cases. The validation set 1 contains 1239 cases with 9340 slides while validation set 2 contains 738 cases with 7140 slides (Table 1). The distributions of clinical metadata in the validation sets are described in Supplementary Table 1, and the Kaplan–Meier curves for all splits are shown in Supplementary Fig. 8.

### DLS overview

The DLS consisted of two separate models applied sequentially. First, a tumor segmentation model was applied on each whole-slide image (WSI) to generate a region of interest (ROI) mask. A prognostic model was then trained and evaluated to predict case-level DSS using image patches sampled from these ROI masks (Supplementary Fig. 2).

### Tumor segmentation model

In order to identify tumor-containing regions at scale, we first developed a model for colorectal adenocarcinoma detection using an approach similar to that previously described^46^. Briefly, a convolutional neural network (CNN) was developed in a patch-based supervised learning approach using WSIs from pathologist-annotated colorectal slides. These annotations involved pixel-level outlines of colorectal adenocarcinoma, normal colorectal epithelium, atypical epithelium, necrosis, and an “other” category comprised of entities within tumor-containing regions such as fibrosis, ulceration, large areas of stroma within the tumor, and areas with evidence of treatment effect. Regions such as normal non-epithelial tissue (e.g., muscle and submucosa) were not annotated. A sample annotated slide is provided in Supplementary Fig. 3.

The 265 annotated slides were split into the train, tune, and test split in a ratio of 3:1:1. After reviewing notes provided by the annotator, 21 slides were dropped either due to slide quality issues or incomplete annotations. This resulted in 149 slides for training, 51 slides for tuning, and 44 slides for testing (all within the training split). A CNN based on the Inception-v3^47^ architecture with reduced parameters (depth_multipler=0.1) was trained to distinguish between adenocarcinoma and all other classes on a per patch basis. Details on model architecture and hyper-parameter tuning are in Supplementary Table 8. The model achieved an AUC of 98.50. This colorectal adenocarcinoma detection model was used to generate ROI masks. Only patches from within the ROI masks were used to train and evaluate the prognostic model.

### Region of the interest mask generation

The tumor model was used to generate binary ROI masks for all slides. Running the tumor model with a stride of 64 (at magnification 20×, 0.5 μm per pixel) resulted in tumor probability heatmaps of resolution 32 μm per “superpixel”. To generate binary ROI masks from the continuous tumor probability output of the tumor model, a threshold *t* was selected to binarize the tumor model output for each patch. Next, denoising was performed by computing the connected components of positive regions and removing components with fewer than eight superpixels. Finally, to include tumor-proximal regions in addition to tumor when training the survival model, the tumor-positive regions from the tumor model were dilated with a circular filter of radius *r*. For optimizing the selection of *t* and *r*, ROI masks were generated for three different values of the probability threshold *t* and the dilation radius *r* when tuning the prognostic model. The thresholds evaluated during tuning corresponded to recall of 95%, 90%, and 75% on the tune split (Supplementary Table 9). The values used for the dilation radius *r* were 0, 4, and 16 superpixels. The threshold *t* and dilation radius *r* were selected to optimize DLS performance on the tune split of the entire development set. During inference, we aligned the ROI masks to the output resolution of the prognostic model (patch size of 256 pixels across at 5× magnification, or 512 μm). Only image patches where at least half of the patch was contained in the ROI mask were used for prognostication.

### Prognostic model neural network architecture and survival loss

The neural network architecture for the prognostic model was designed to predict a case-level risk score given a set of image patches sampled from the tumor containing regions in a case and was previously described^48^. The architecture consisted of several CNN modules with shared weights for extracting dense feature vectors from each input patch, an average pooling layer for merging the set of patch-level feature vectors into a single case-level feature vector, and a final Cox regression layer for computing a scalar case-level risk score (see Supplementary Fig. 2C). The CNNs consisted of depth-wise separable convolution layers, similar to the design of MobileNet^48,49^. This type of convolution layer has fewer parameters than standard convolution layers, which reduces computation and helps avoid overfitting. The filter size in each layer and the number of layers were tuned via a random grid-search^50^ (Supplementary Table 10).

For the weakly supervised survival prediction tasks, the location of informative patches in each WSI is not known. Our approach of randomly sampling *n* patches within each slide helped ensure informative patches were selected during training. If each patch has a certain probability of being informative, the probability of not sampling any informative patches decreases exponentially with the increase of *n*. This approach also generalizes to different numbers of slides per case, enabling use in real-world datasets that may contain many slides per case (average of 18 slides/case in our study).

The loss function during training was the Cox partial likelihood^51^, which was selected based on a preliminary experiment on the tune set where it performed the best (by a small margin) amongst the three survival loss functions (Supplementary Fig. 9). By contrast, in our prior work with different cohorts, different inclusion criteria, and only one slide per case^48^, the censored cross-entropy loss function performed better, indicating value in further work to better understand the optimal loss function. The Cox partial likelihood is formulated as follows:

1 $$\max \mathop {\prod }\limits_{i:O_i = 1} \frac{{e^{f(X_i)}}}{{\mathop {\sum }\nolimits_{j:T_j \ge T_i} e^{f(X_j)}}}$$

where for the *i*th case, *T*_*i*_ is the event time or time of last follow-up, *O*_*i*_ is an indicator variable for whether the event is observed, *X*_*i*_ is the set of WSIs. The function *f* represents the prognostic model, and *f*(*X*_*i*_) is the scalar case-level risk score. In our implementation, we used Breslow’s approximation^52^ for handling tied event times due to its simplicity of implementation. During training, we approximated the full loss at each training step by evaluating it over the examples in the training batch.

### The prognostic model training procedure

The prognostic model was trained on both stage II and stage III cases. Training examples consisted of sets of 16 image patches per case sampled randomly across regions of interest produced by the ROI model. Images were first normalized to a standard color distribution based on the color statistics in the training set^46^ and then augmented by color and orientation perturbations described previously^46^. Numerical optimization of network parameters was done using the Adam optimizer^53^. Hyperparameters governing ROI mask generation, patch extraction, model architecture and optimizer were tuned by selecting the best performing configuration across 100 random configurations from the full hyperparameter search space (Supplementary Table 10). Models were trained for 2 million steps in a distributed fashion, using 50 workers with 16 CPU processors each.

### Prognostic model evaluation procedure

Each model was evaluated every 10,000 steps on the tuning set using a sample of 1024 patches per case. The best checkpoint for each model was selected by taking the maximum after applying a rolling average with a window size of 10. The best checkpoints for five models that achieved the highest c-index on the tuning set were ensembled to form the final prognostic model. To generate a case-level prognostic risk score, the ensembled prognostic model was run exhaustively over all non-overlapping patches within the ROI mask.

### Evaluating DLS performance

We used three evaluation metrics to assess the prognostic ability of the DLS for DSS: 5-year survival AUC, hazard ratio, and c-index. All analyses were done for stage II and stage III independently, and for the two stages combined. The 5-year AUC was used because every case in both validation sets had at least 5 years of follow-up. The 5-year AUC was used because every case in both validation sets had at least 5 years of follow-up. These analyses (as well as the data splits and inclusion/exclusion criteria) were pre-specified and documented prior to running the model on the validation sets. Multivariable hazard-ratio analyses for Stage II and Stage III were pre-specified as the primary analyses. In the two validation sets, 10% of examples were censored prior to 5 years due to non-disease-specific death; these examples were excluded for the purposes of 5-year DSS AUC computation, but incorporated as right-censored for hazards ratio and c-index computation. The 5-year AUC for the clinicopathological variables and the combination of these clinicopathological variables with the DLS was computed using the sklearn.metrics.roc_auc_score function in the Python sklearn package (v0.23.2).

To compute the hazard ratio for the DLS as well as the clinicopathological variables, Cox proportional hazards regression models^54^ were used. The case-level DLS scores and age were treated as numeric variables. DLS predictions were rescaled to have zero mean and unit variance. Age was centered at the mean age scaled down by a factor of 10, such that the hazard ratio for age corresponds to the risk increase per decade of age. All other variables were coded as categorical (dummy/indicator) variables. Survival times were discretized into months for all analyses.

Cox regression models were also used to calculate c-indices^55^ for the DLS, the clinicopathologic features alone (baseline model), and for the DLS combined with these variables (combined model). These Cox models were fit on the tune set and applied to both validation sets. C-indices were computed using the lifelines.utils.concordance_index() function in the Python Lifelines package (v0.24.6). Confidence intervals for the c-index were generated via paired bootstrap resampling with 9999 samples.

For Kaplan–Meier analysis, cases were stratified into low- and high-risk groups using thresholds determined using cases from 2002 to 2007 on the tune set, to account for the temporal shift in case characteristics shown in Supplementary Table 1. The low-risk threshold is the 25th percentile of the tune set risk scores, while the high-risk threshold is the 75th percentile of the tune set risk scores. Different thresholds were selected for stage II cases, stage III cases, and the combination of stage II and stage III cases. The Python Lifelines package (version 0.24.6)^56^ was used for Kaplan–Meier analysis and Cox regression analyses, using the lifelines. KaplanMeierFitter and lifelines.CoxPHFitter classes. The REMARK checklist for reporting is provided as Supplementary Table 11.

### Understanding DLS predictions

The following analyses were conducted in an exploratory manner after the DLS was applied to the validation sets. All annotations and histologic reviews were performed with the raters blinded to both the DLS’s predictions and the outcomes of the relevant case.

### DLS association with clinicopathologic features

The association of the DLS with case-level clinicopathologic features was evaluated via multivariable linear regression (Table 4). The case-level DLS scores were standardized to have zero mean and unit variance. All clinicopathologic features except age (at the time of diagnosis) were coded as indicator variables. Age was centered at the mean age and scaled down by a factor of 10, such that the coefficient for age corresponds to the risk increase per decade of age. The proportion of variance in DLS scores explained by these features was evaluated using the adjusted coefficient of determination (*R*^2^).

### DLS association with clustering-derived features

We next studied the association of the DLS with histologic features derived from clustering (Table 5). To obtain these histologic features, we leveraged a previously-described image-similarity deep learning model^25,26^ that was trained to distinguish between similar and non-similar natural (non-histopathology) images. This model was used to generate patch-level embeddings that captured visual similarity. Embeddings for a sample of 100,000 tumor-containing training set image patches were clustered using the *k*-means algorithm as implemented in the Python sklearn package (v0.21.3). The total number of clusters (k) was chosen based on the fit on the tune set (described next) when using the best subset of ten features (described next). Values of *k* explored were: 10, 25, 50, 100, 200, 300, 400, and 500. *K* = 200 clusters were found to be optimal. The centroids of these 200 clusters, which were fit on the sample of patches from the training set, were used to assign each tumor-containing patch in both validation sets to a cluster. For each case, the percentage of patches belonging to each cluster was computed.

Next, the association of the DLS with these features was also evaluated via multivariable linear regression in a similar manner to the procedure for clinicopathologic features above. The clustering-derived features were scaled to range from 0 to 100, indicating the percentage of tumors in the case belonging to each feature. A subset of 10 features was selected for more in-depth characterization from the full set of 200 features using forward stepwise selection with the objective of maximizing the *R*^2^.

To provide morphological descriptions for the subset of 10 features, 15 patches per feature were presented independently to two pathologists for review (a subset of 10 per feature is shown in Fig. 2). The selected patches were those that were closest to each feature’s centroid (and filtered to ensure that for each feature, each patch was sampled from a different case). The pathologists were blinded to any additional information about the feature and provided histopathological review via a structured form. The presence of a tumor, stroma, adipose, and TILs was scored semi-quantitatively as absent, low, medium, or high. The tumor was graded as low, intermediate, or high grade, and fibrosis (if present) was graded as mature, intermediate, or immature^57^. Additional free-text descriptions of tumor and stroma for each cluster were also provided by each pathologist.

### DLS association with patch-level histoprognostic features

To evaluate the association of DLS predictions with known histoprognostic features, we annotated 161 slides for several known features previously reported to be associated with adverse prognosis in colorectal cancer. The slides used for this purpose were randomly selected from 161 cases in the validation set 2. Annotated features included lymphovascular invasion, perineural invasion, intratumoral budding, peritumoral budding, and peritumoral fibrosis. When present, polyps were also annotated to provide another histologic class for comparison. Board-certified pathologists (without gastrointestinal subspecialty training, median pathologist experience: 6.5 years post-training, range 3–17 years) were asked to exhaustively annotate the tumor-containing regions of each slide for these features.

For each histoprognostic feature, the average patch-level DLS score among all patches annotated for that feature was computed. For comparison, we also computed the average patch-level DLS score among all patches for each clustering-derived feature. For both analyses, 95% confidence intervals for the average DLS score were computed via blocked bootstrapping at the slide-level.

### Tumor-adipose feature

To understand if people could accurately identify TAF, we extracted both TAF-containing and non-TAF-containing image patches from tumor-containing regions (based on the tumor segmentation model). Each patch was 256 × 256 pixels at 5× magnification (0.5 mm^2^). Participants have first presented 50 TAF patches (as determined by the clustering algorithm) as learning material (see Supplementary Data 1). Of these, 25 (Fig. 3a) were closest to the centroid and thus the most representative of the cluster-derived feature. Another 25 patches were randomly sampled from the cluster. These randomly sampled patches potentially included examples without the pathologist-identified tumor adipose feature and were included to provide examples of the diversity of patches assigned to the cluster. Within each set of 25, each patch came from a distinct case. The participants (two anatomic pathologists: I.F-A. and T.B. and three non-anatomic-pathologists: E.W., D.F.S., and Y.L.) reviewed the above material and then completed a separate practice round of indicating whether they perceived each of 50 additional patches to be TAF or not (see Supplementary Data 2). Clustering algorithm labels were subsequently provided as feedback. Finally, we prepared an independent set of 200 patches, of which 100 were randomly sampled from all patches classified by the clustering algorithm as TAF, while the remaining 100 were randomly sampled from all patches not classified as TAF (see Supplementary Data 3). The participants again indicated whether each patch was TAF or not. To avoid biasing this study of the cluster-derived feature, these patches were not otherwise filtered or reviewed by a pathologist to fit any annotator’s mental concept of TAF. As an additional exploratory analysis, we also generated TAF patches by finding cluster centroids using the validation set 2 instead of the validation set 1, with similar results (Supplementary Fig. 10).

### Model inference speed

The inference timings per case are 11 ± 7 min (±standard deviation) for a single machine with 16 cores; 13 ± 8 s for 50 such machines in a cloud environment; and 8 ± 5 s for a commercially-available accelerator, Google Cloud Tensor Processing Unit (v2). These timings range from being comparable to significantly faster than slide preparation and digitization, which can take a few minutes per slide (multiplied by about ten slides per case on average; See Table 1).

### Reporting summary

Further information on research design is available in the Nature Research Reporting Summary linked to this article.

## Supplementary information

Supplementary Data 1

Supplementary Data 2

Supplementary Data 3

Supplementary Information

Reporting Summary

## Data Availability

This study utilized archived anonymized pathology slides, clinicopathologic variables, and outcomes from the Institute of Pathology and the Biobank at the Medical University of Graz. Interested researchers should contact K.Z. to inquire about access to Biobank Graz data; requests for non-commercial academic use will be considered and require ethics review prior to access.

## Code availability

The deep learning framework (TensorFlow) used in this study is available at https://www.tensorflow.org/; the custom deep learning architecture, custom loss function, sample training code, and statistical analysis code is provided at https://github.com/Google-Health/google-health/tree/master/colorectal-survival and leverages standard depth-wise separable convolutions available from Keras as SeparableConv2D: https://github.com/tensorflow/tensorflow/blob/v2.1.0/tensorflow/python/keras/layers/convolutional.py.
