# Supplementary figures and images for: Interpretable survival prediction for colorectal cancer using deep learning
Source: NPJ Digit Med. 2021 Apr 19;4:71. doi: 10.1038/s41746-021-00427-2 (PMC8055695; doi:10.1038/s41746-021-00427-2)

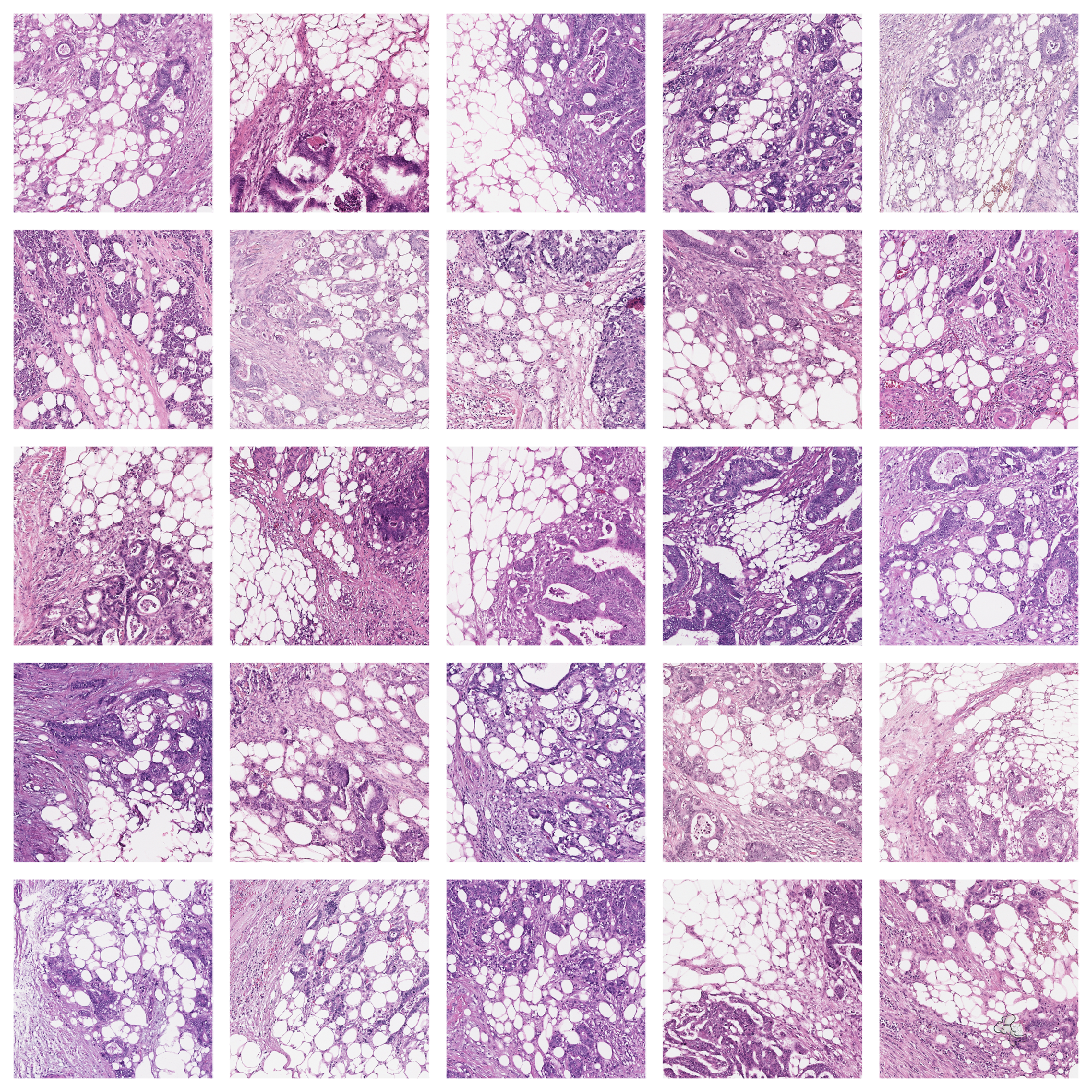

Supplement: Supplementary file 1 — Supplementary Data 1 [file 41746_2021_427_MOESM1_ESM.zip › TAF_examples_closest_to_centroid.png]

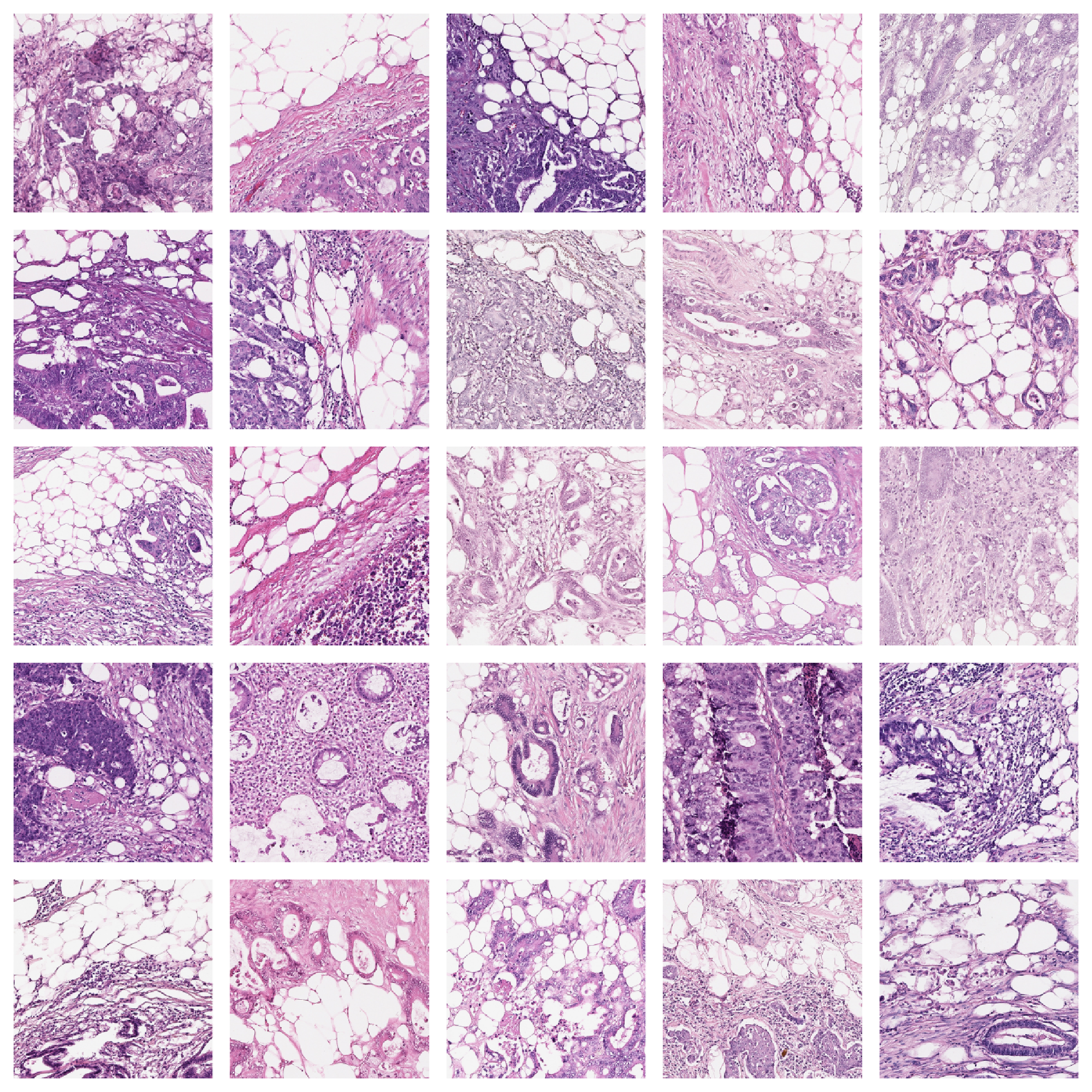

Supplement: Supplementary file 1 — Supplementary Data 1 [file 41746_2021_427_MOESM1_ESM.zip › TAF_examples_random_sampling.png]

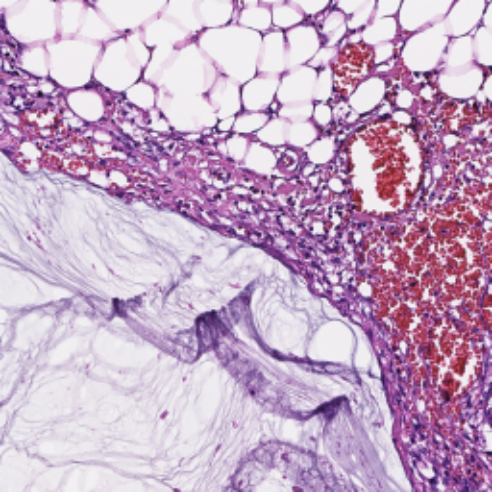

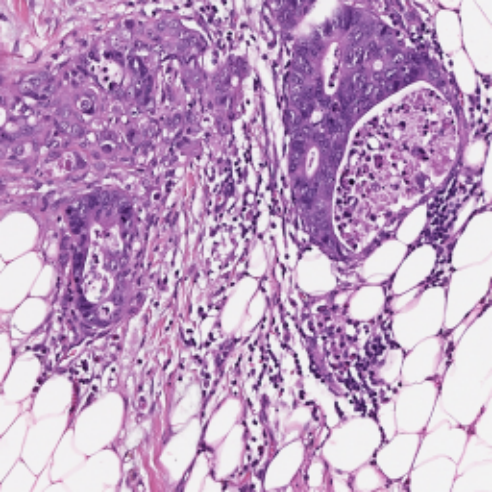

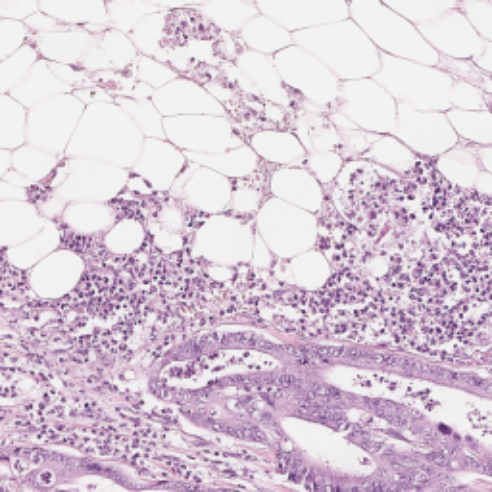

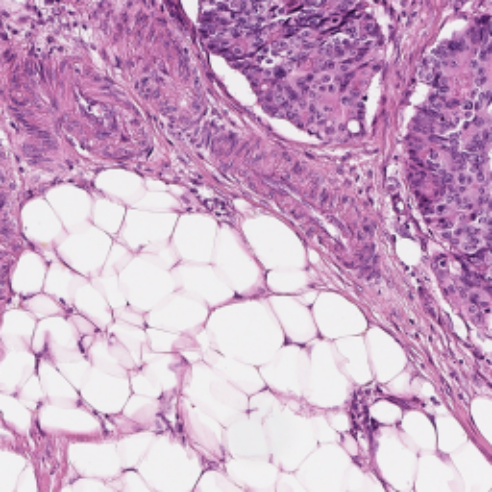

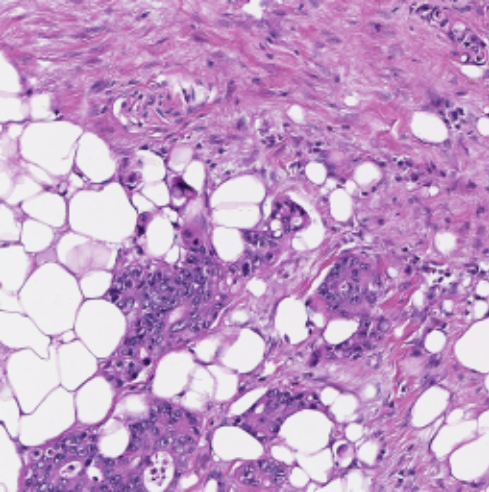

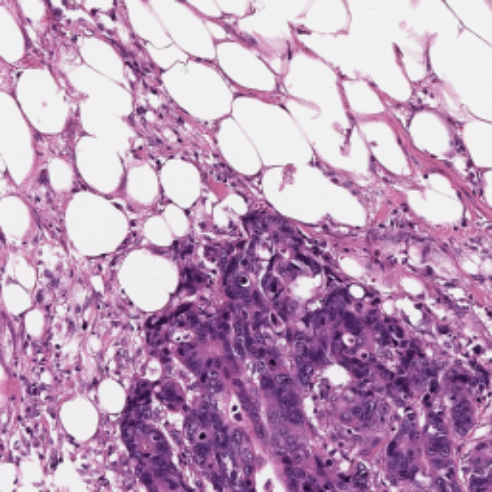

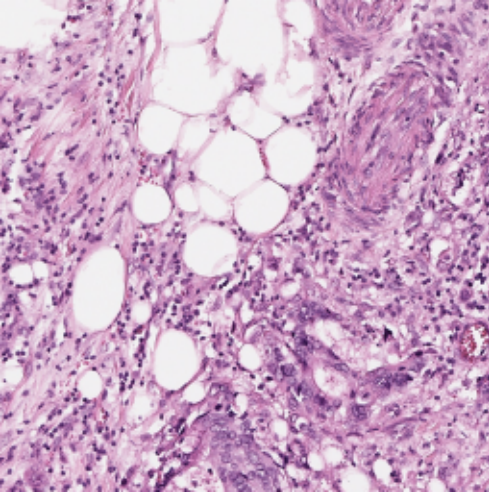

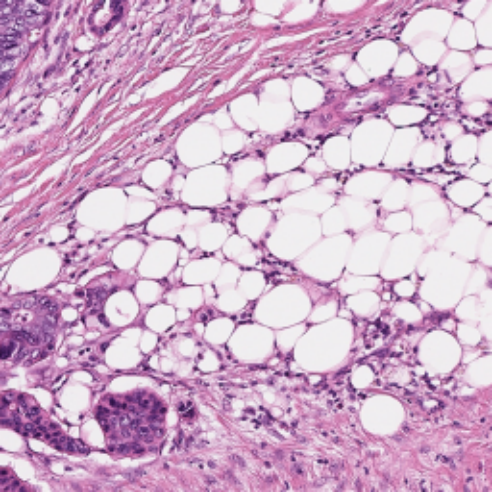

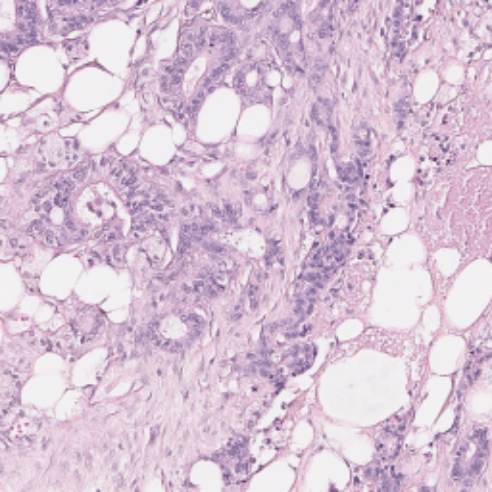

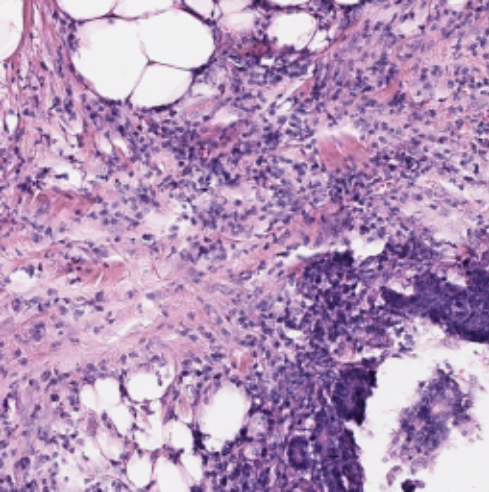

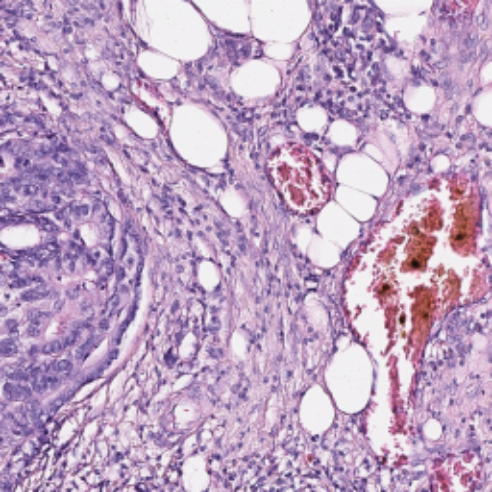

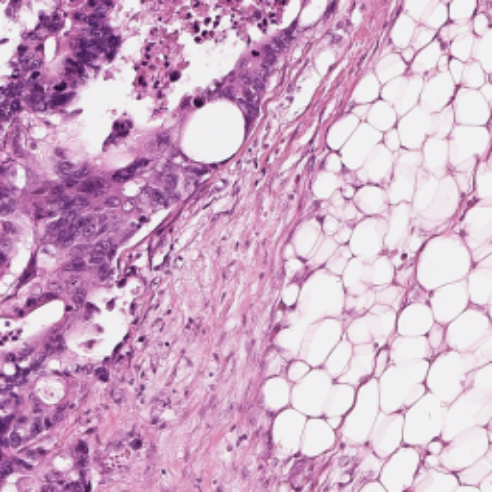

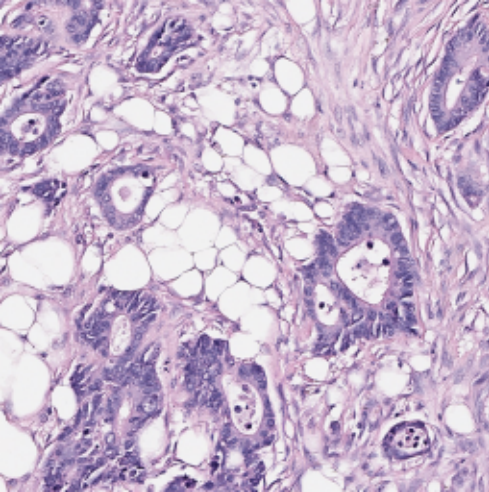

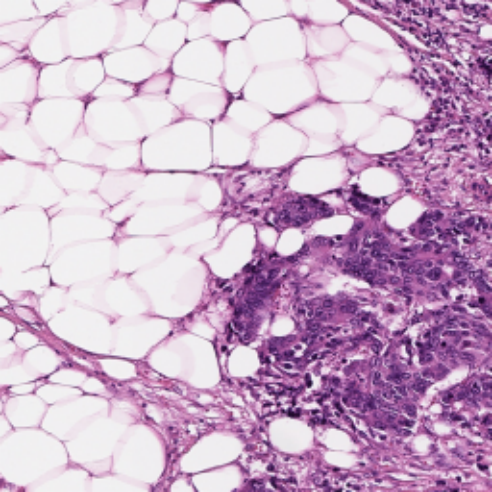

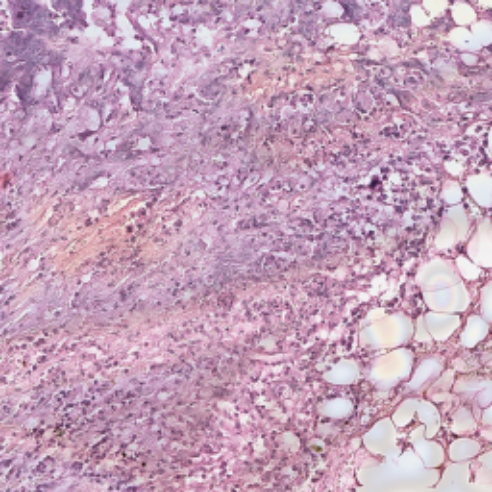

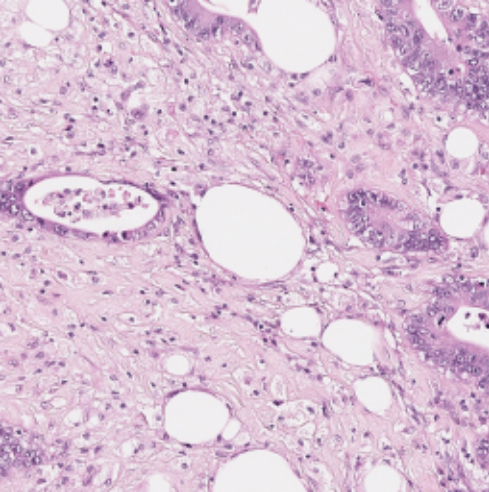

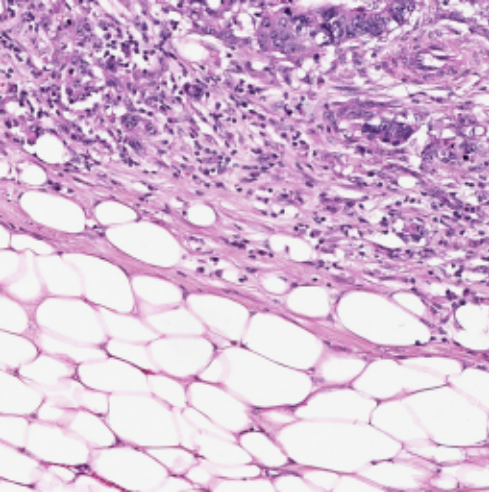

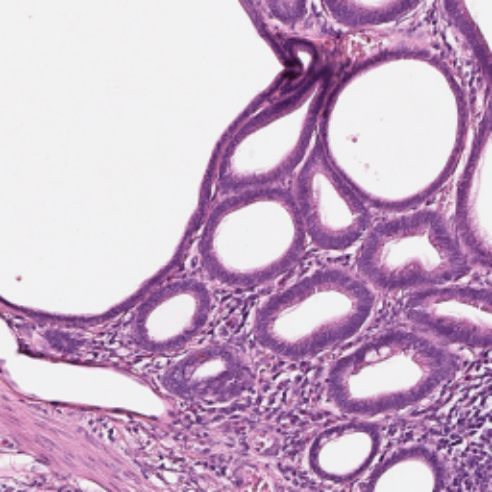

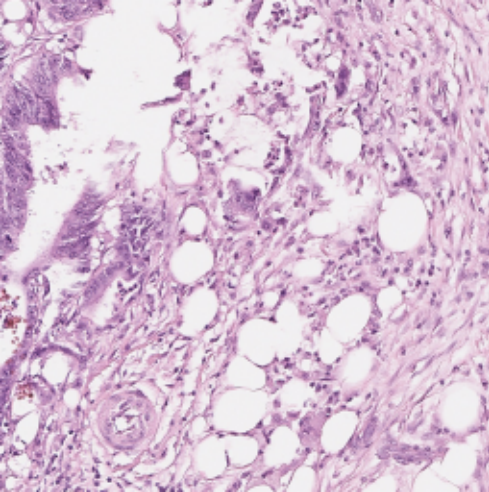

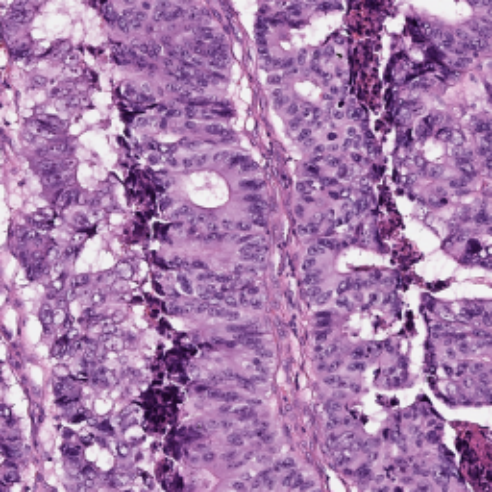

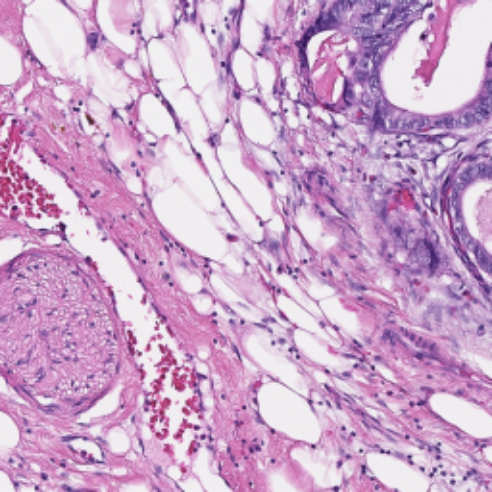

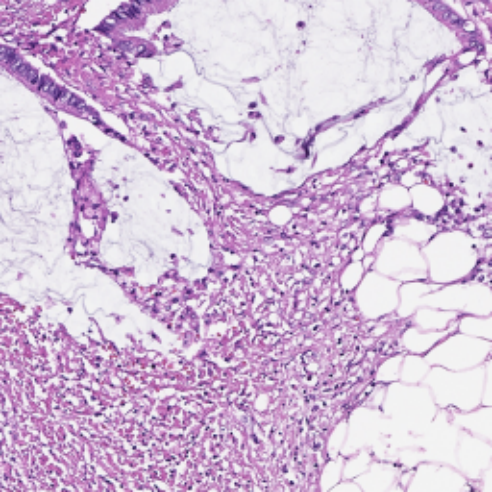

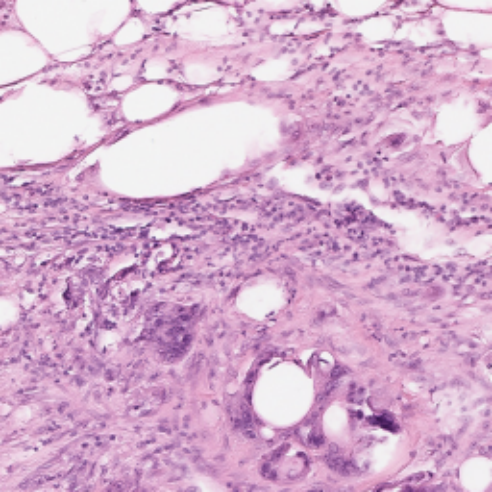

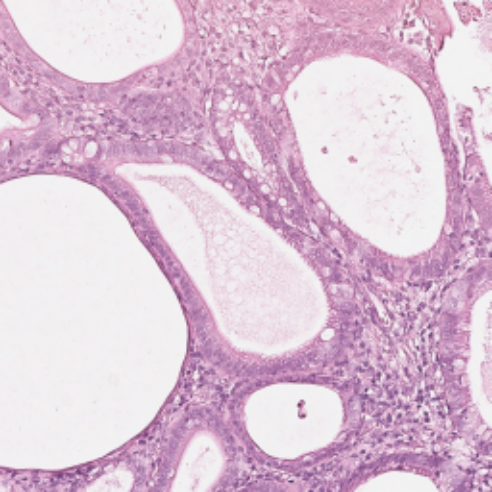

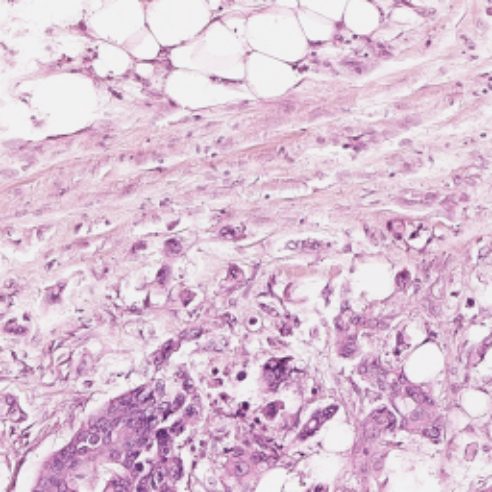

Supplement: Supplementary file 2 — Supplementary Data 2 [file 41746_2021_427_MOESM2_ESM.zip › practice_patches_TAF.pdf]

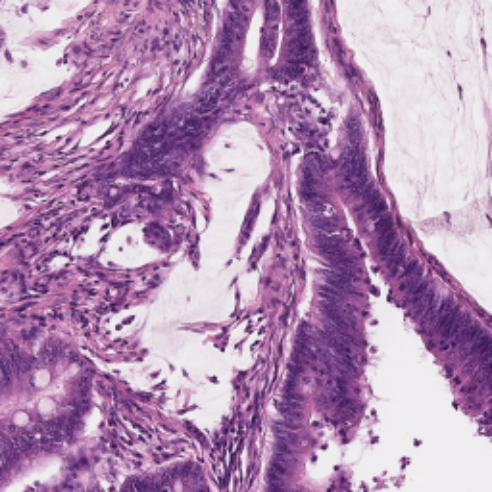

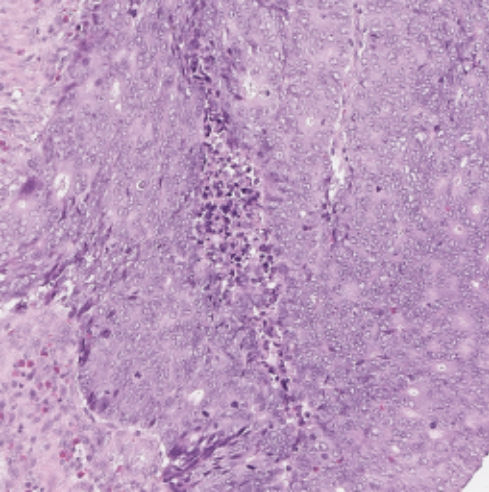

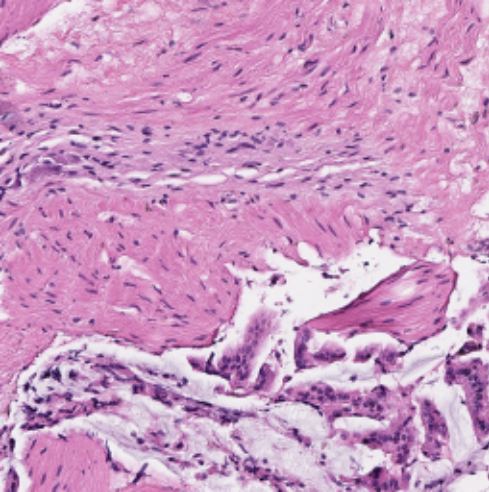

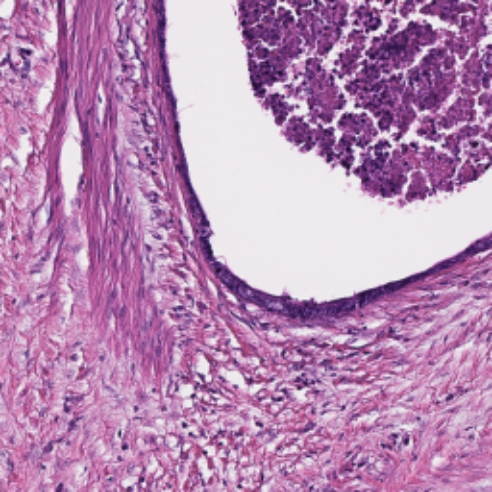

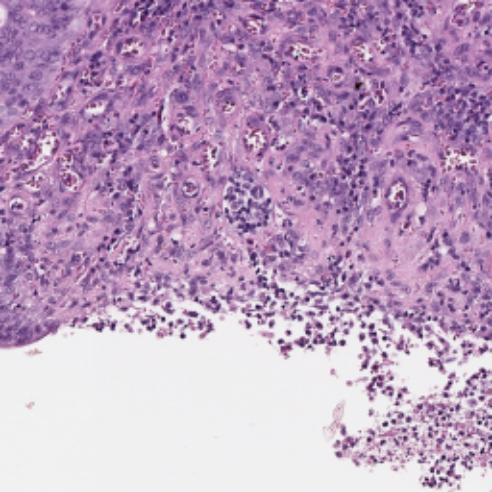

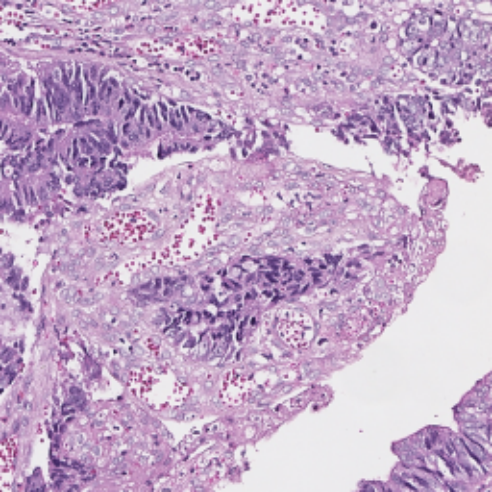

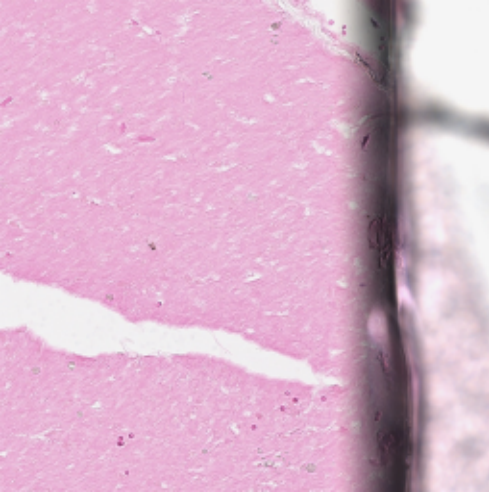

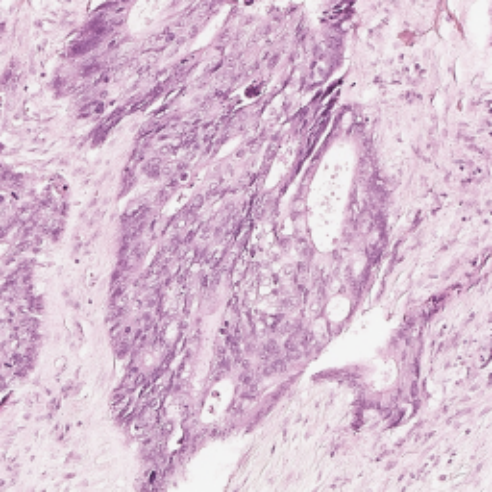

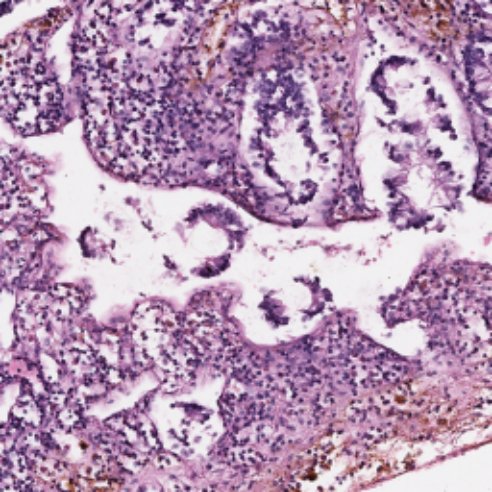

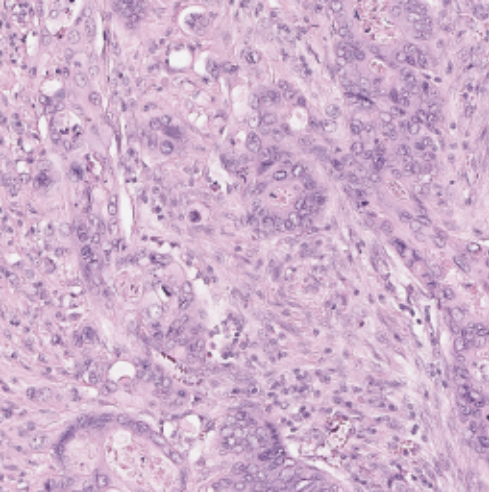

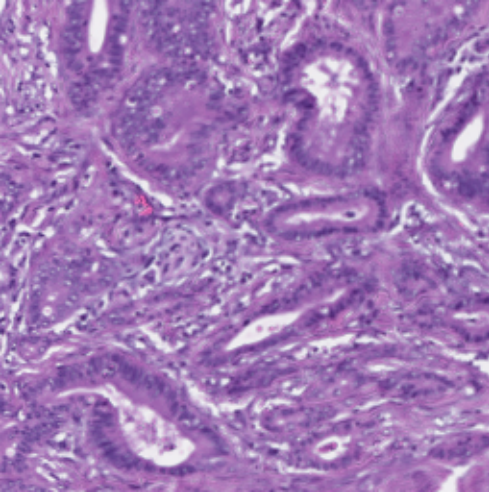

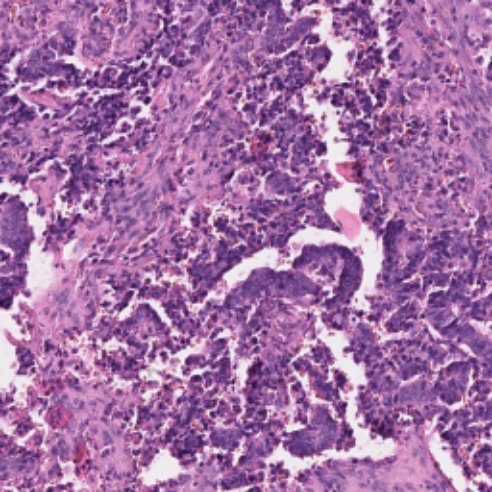

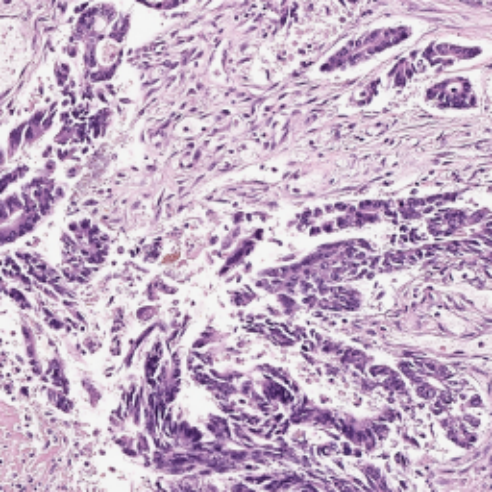

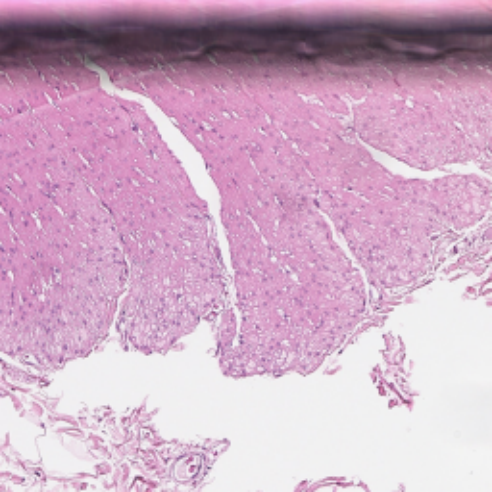

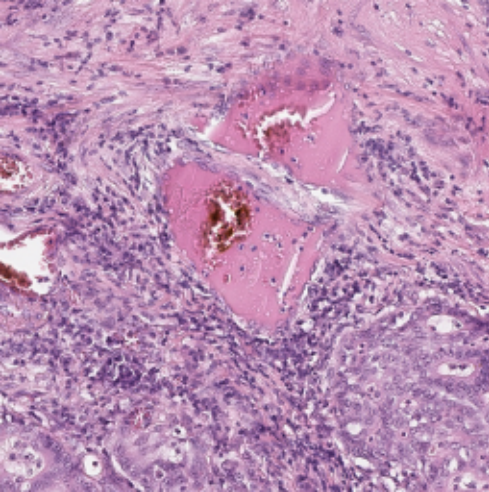

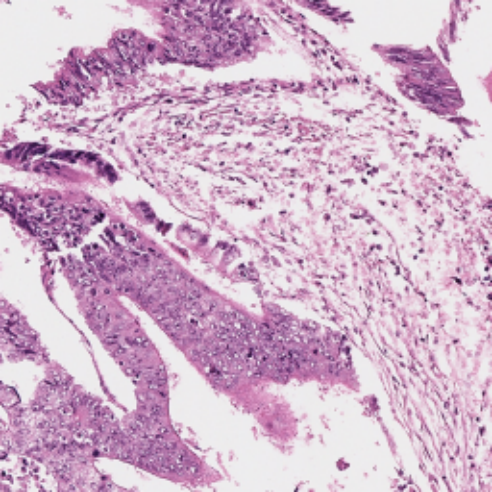

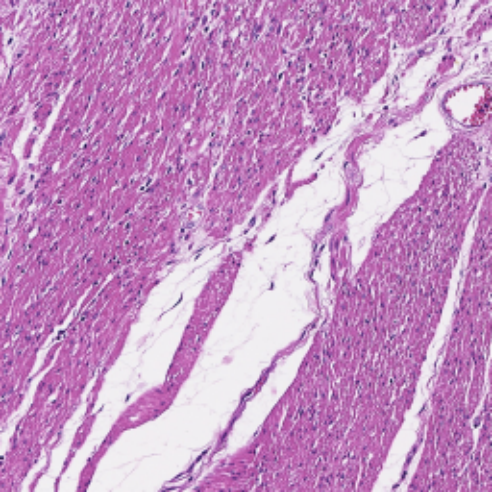

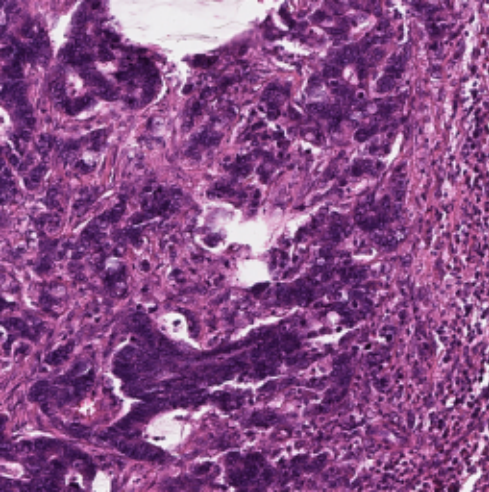

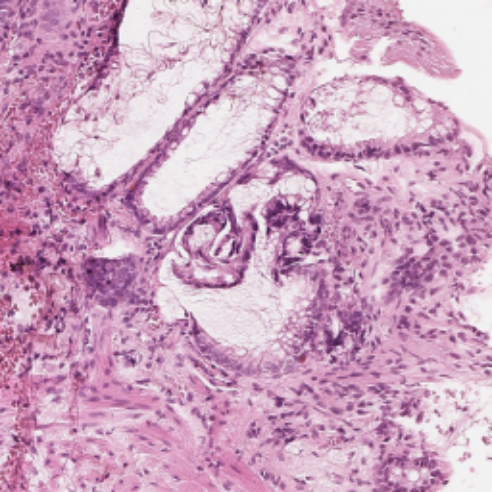

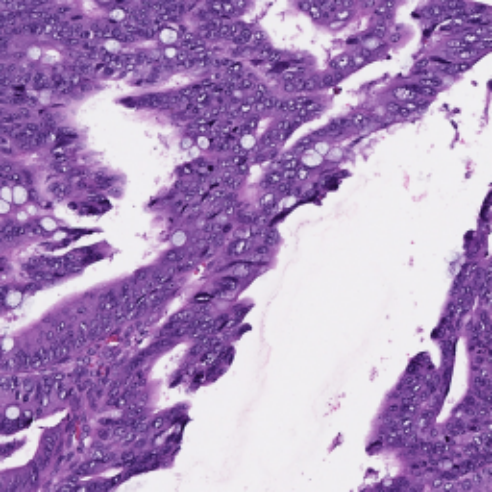

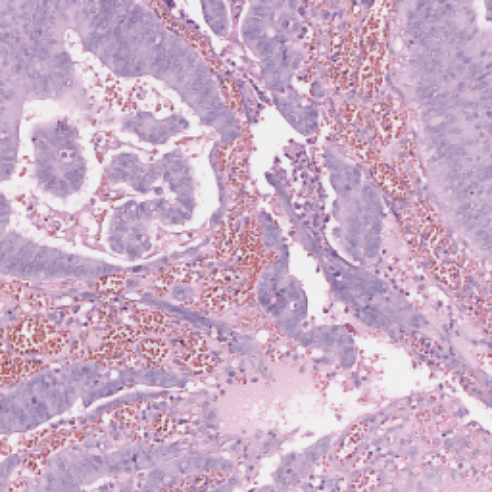

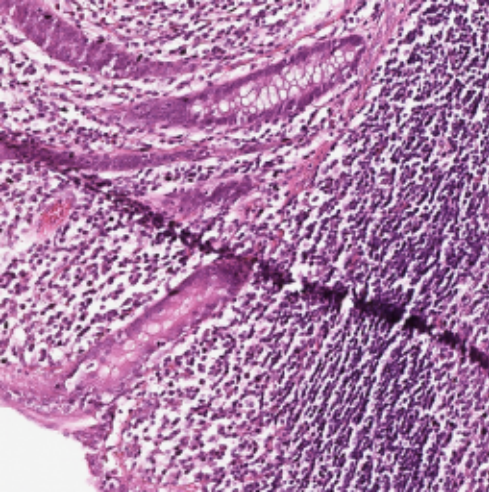

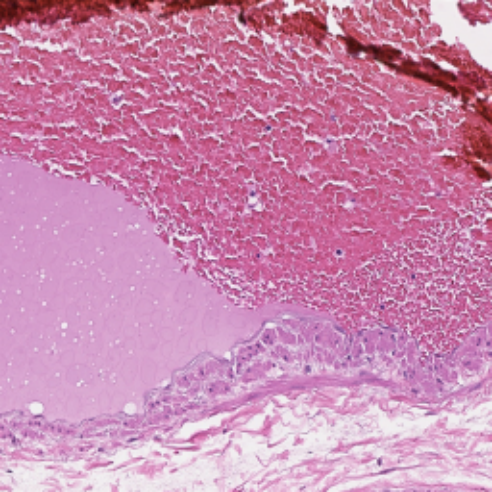

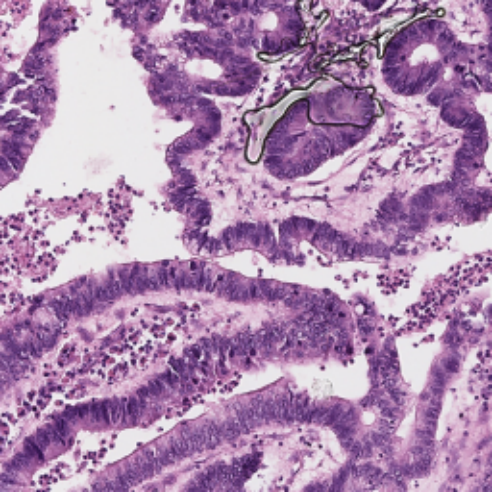

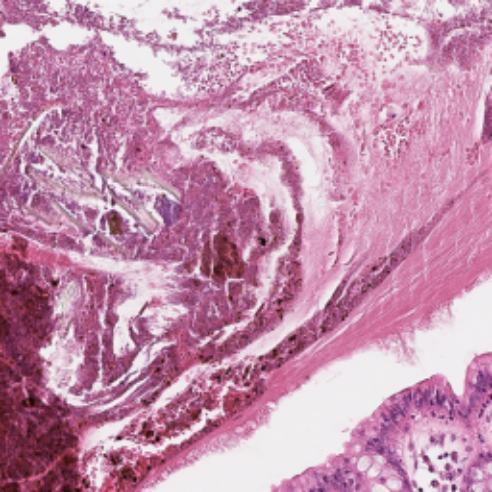

Supplement: Supplementary file 2 — Supplementary Data 2 [file 41746_2021_427_MOESM2_ESM.zip › practice_patches_non-TAF.pdf]

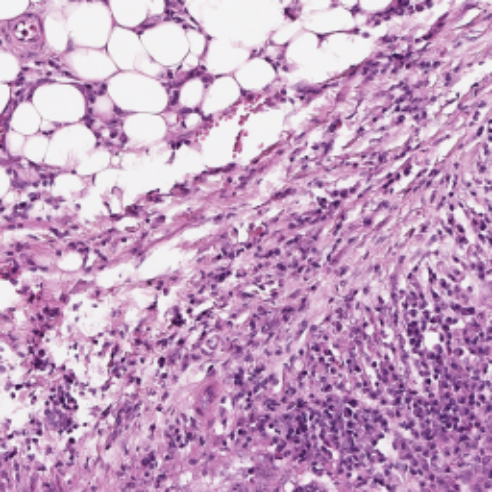

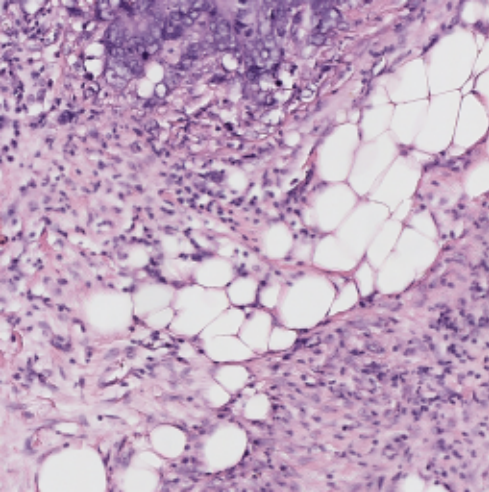

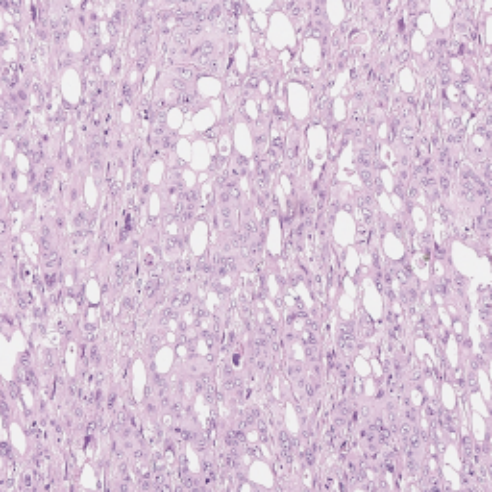

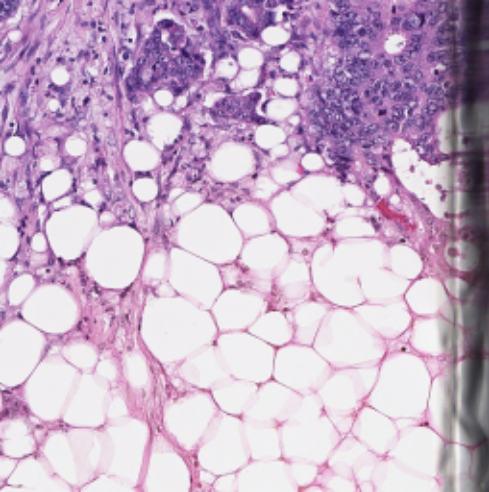

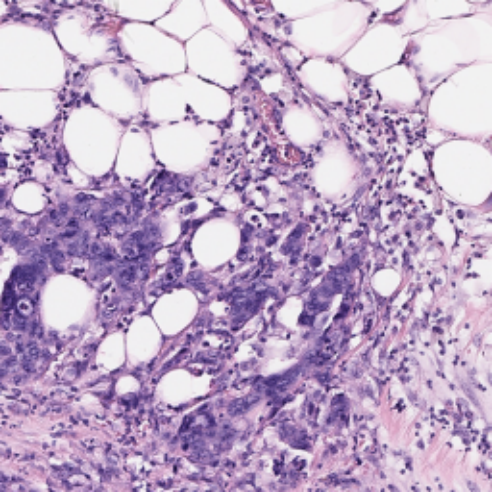

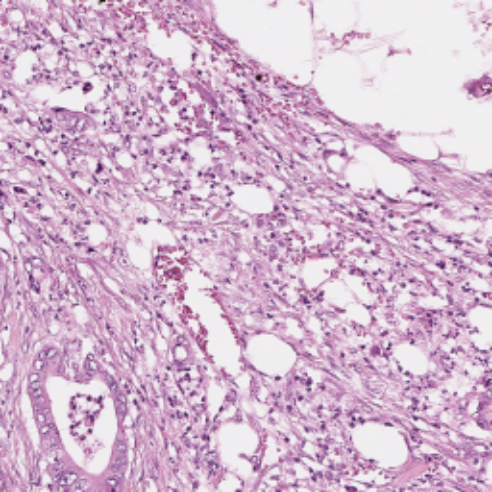

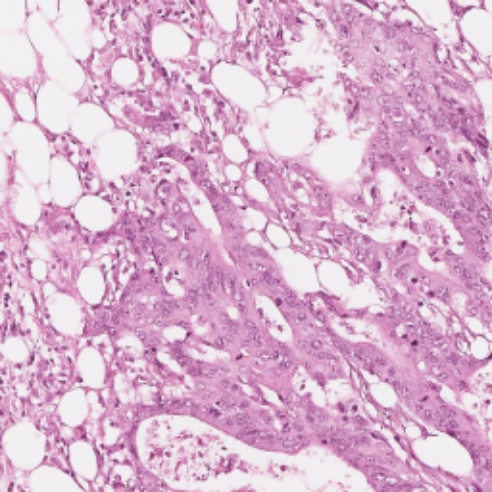

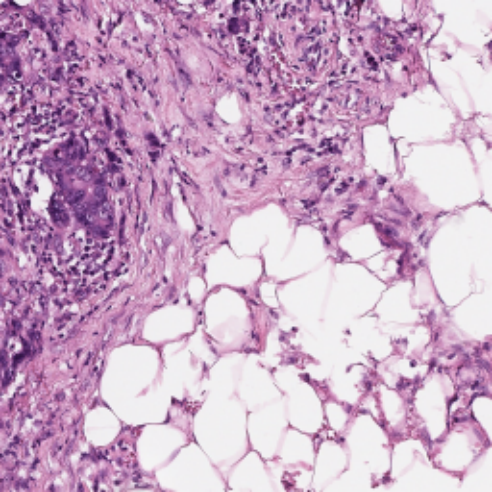

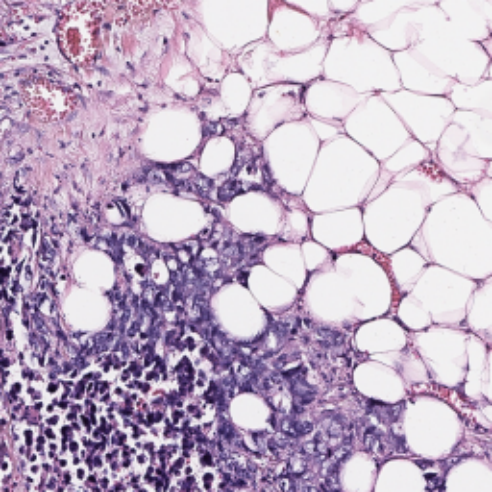

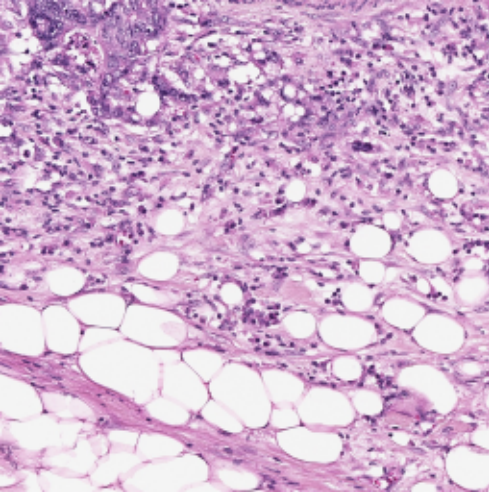

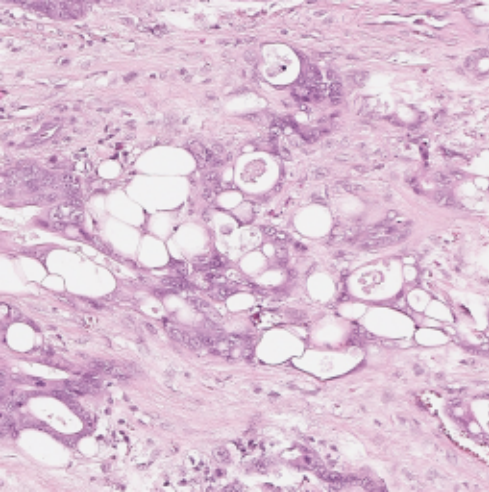

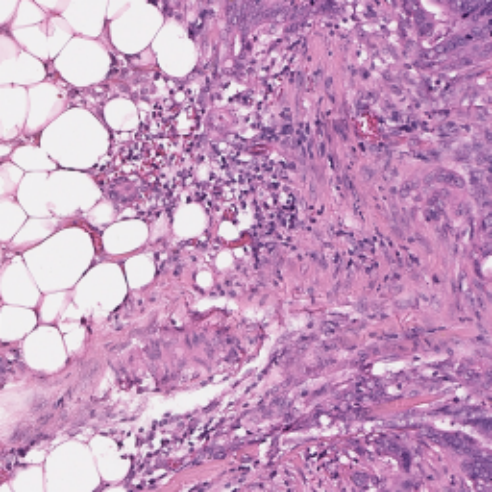

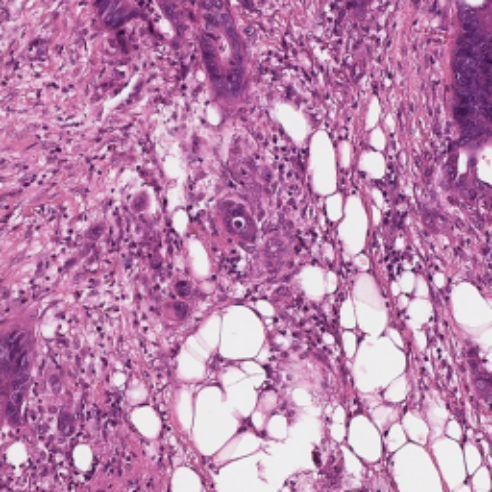

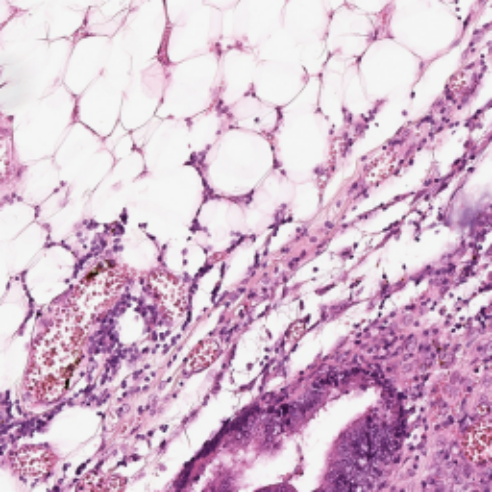

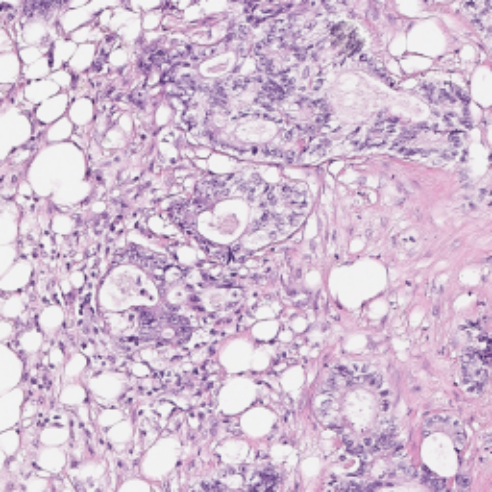

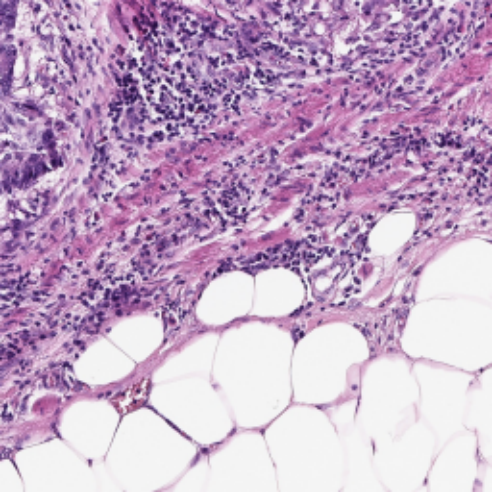

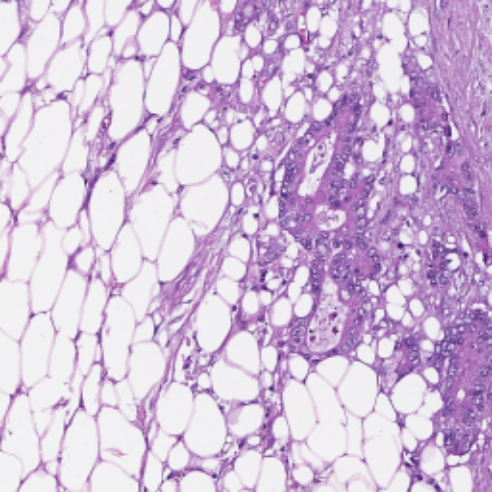

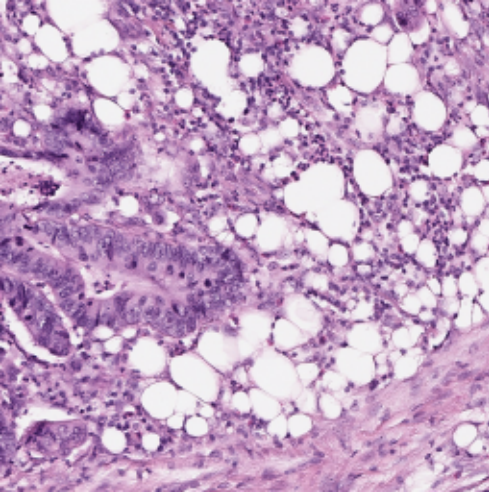

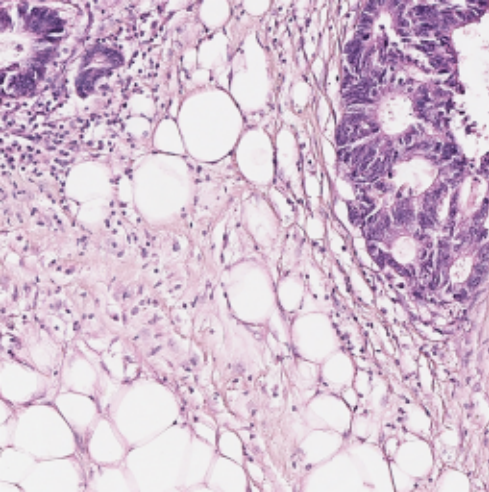

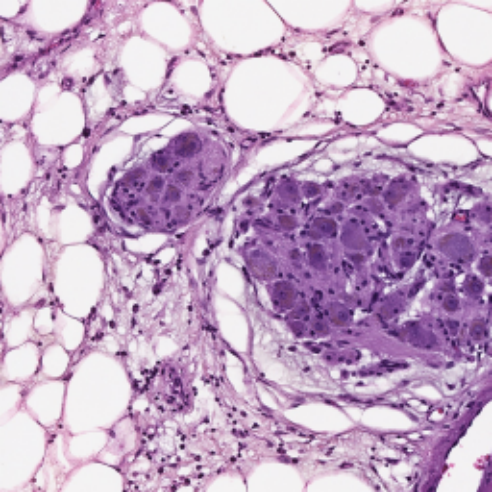

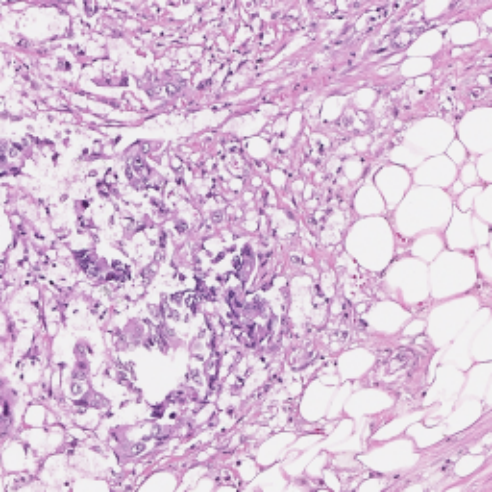

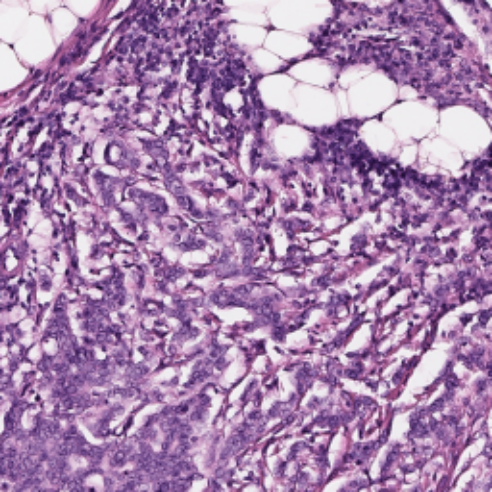

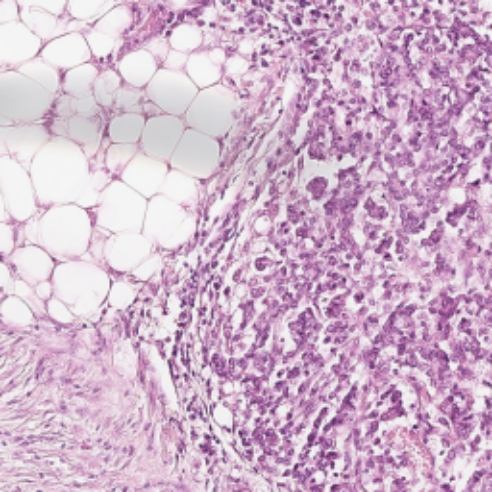

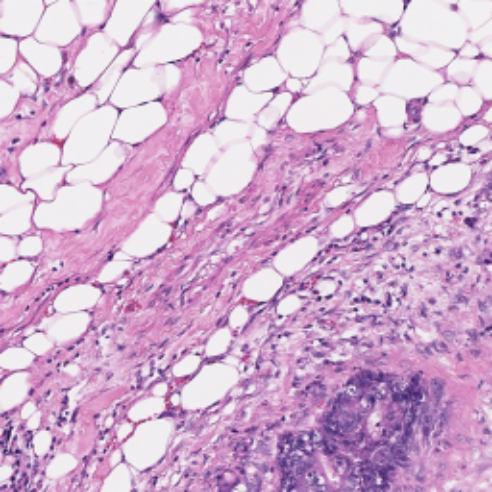

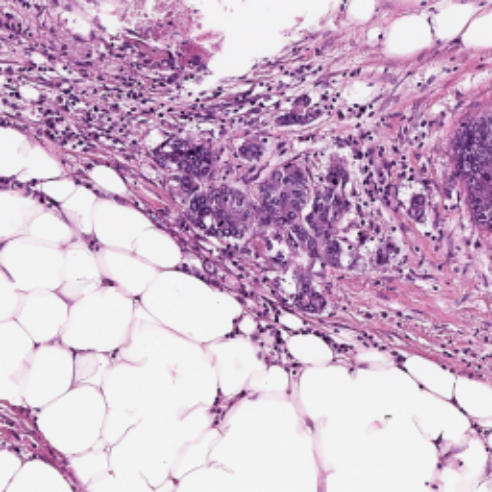

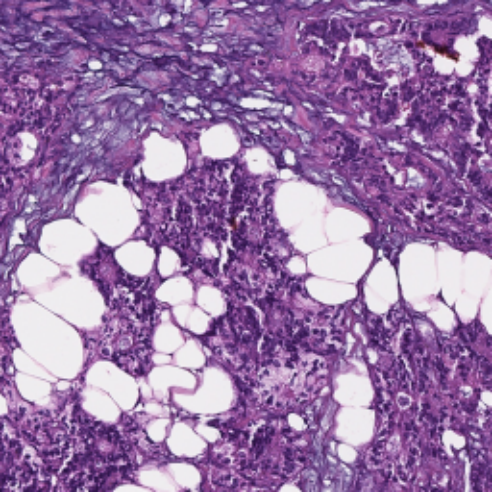

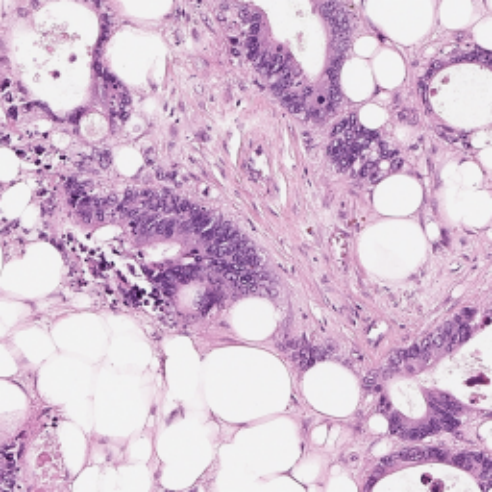

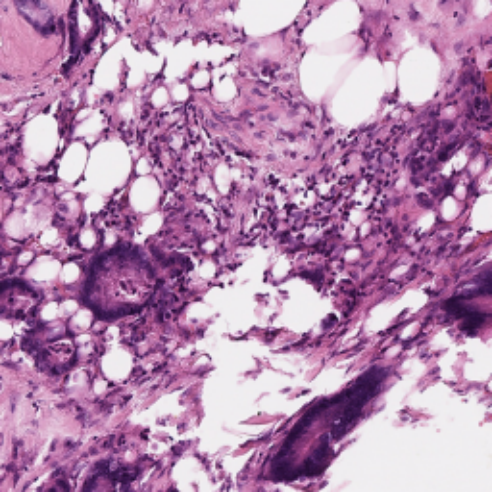

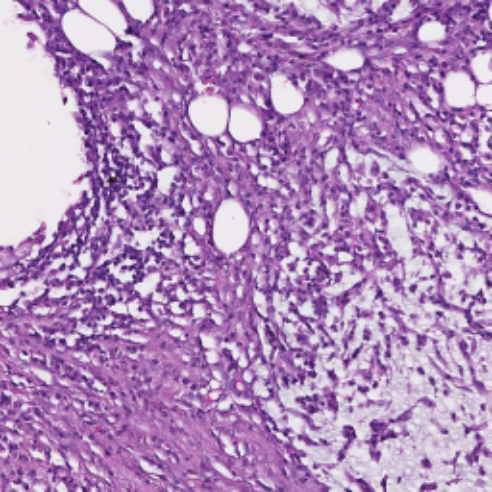

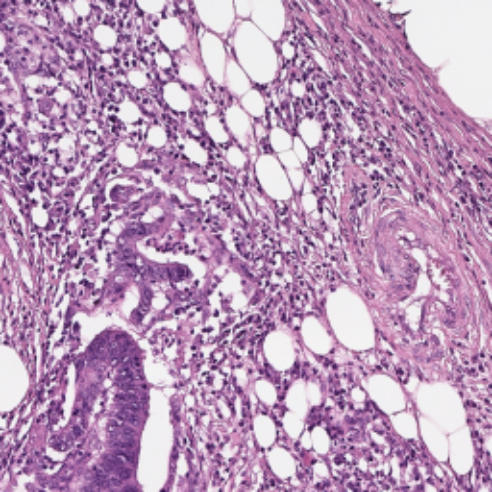

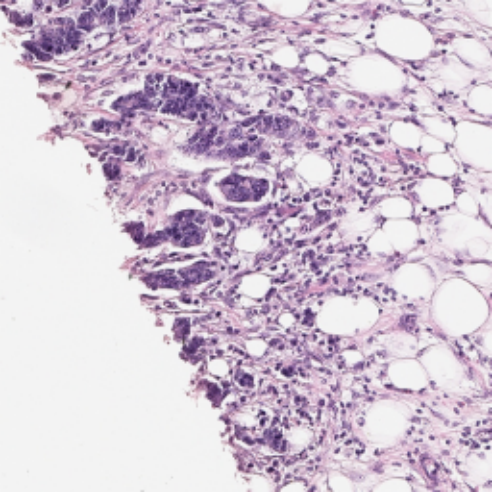

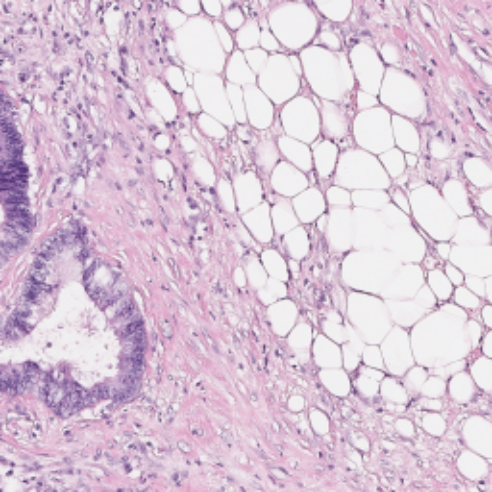

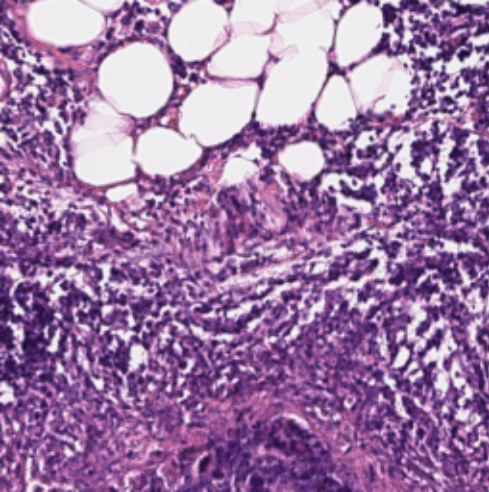

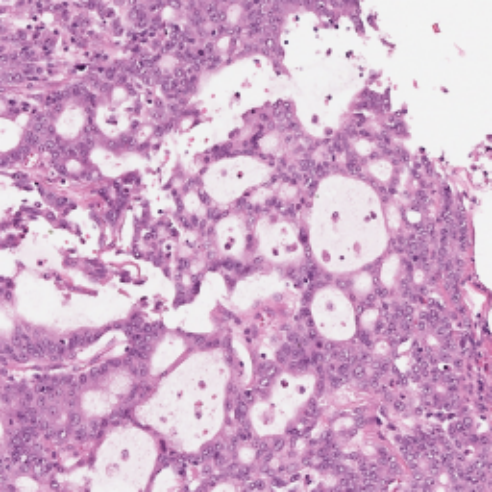

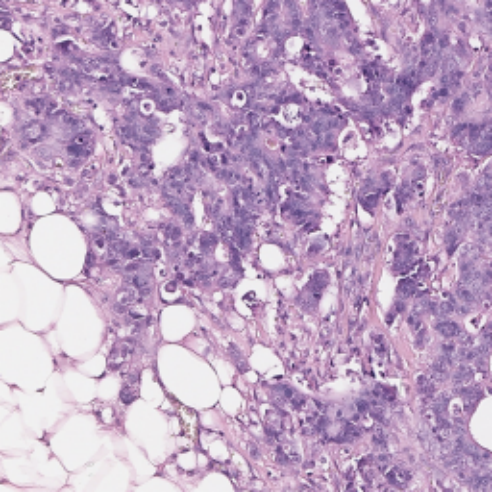

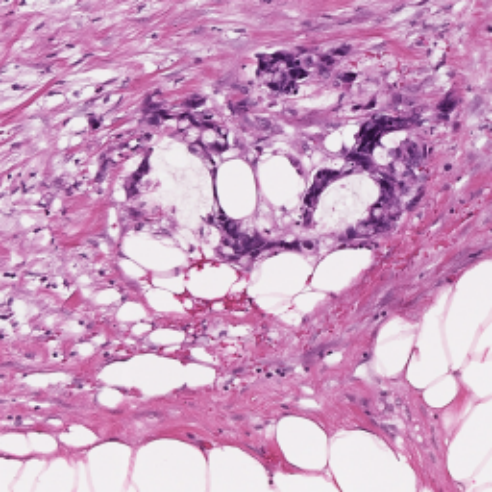

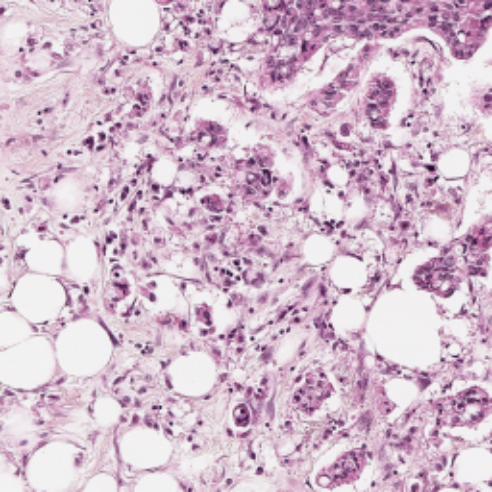

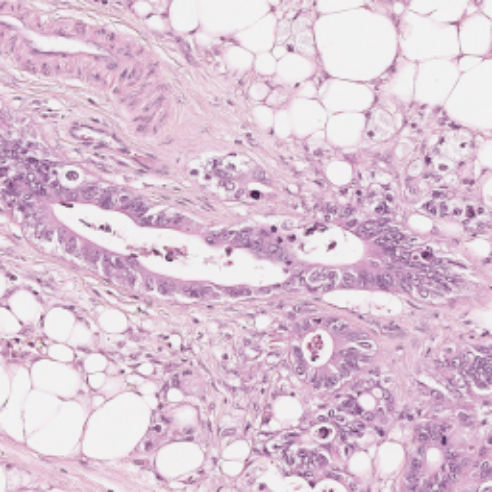

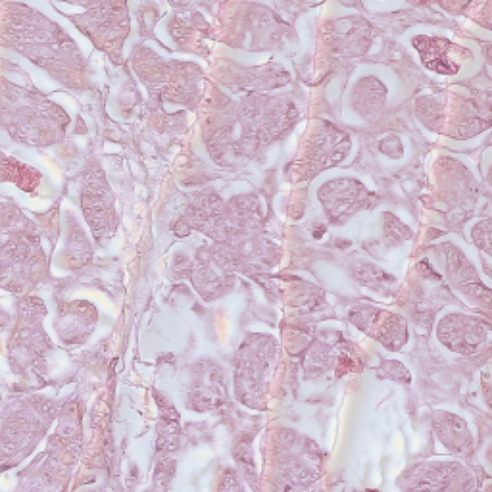

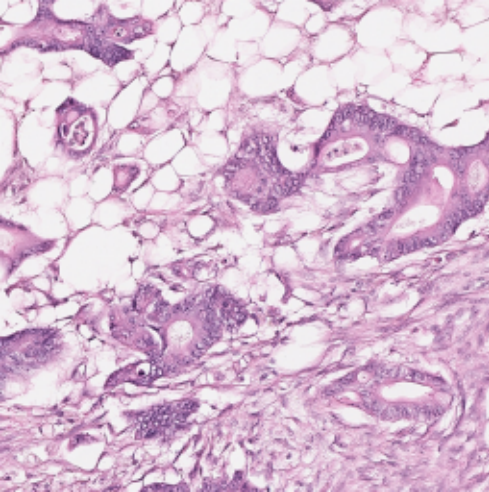

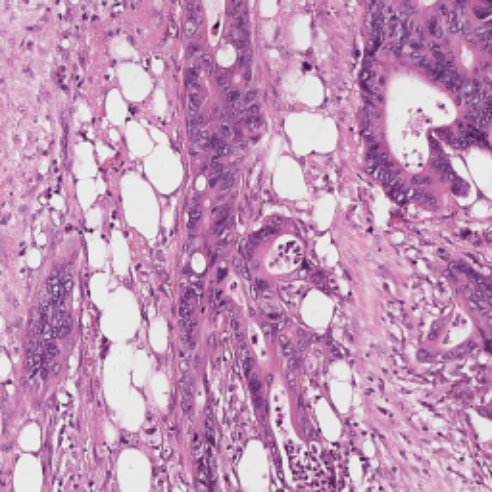

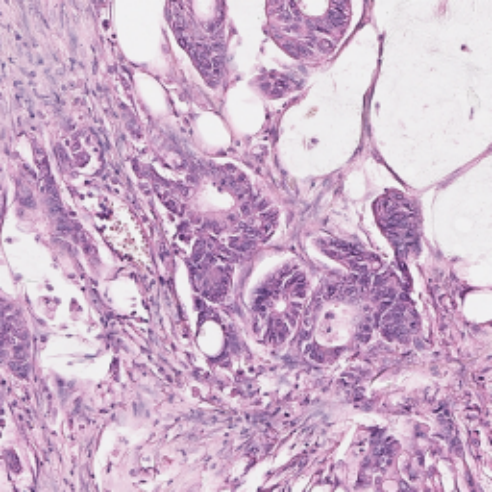

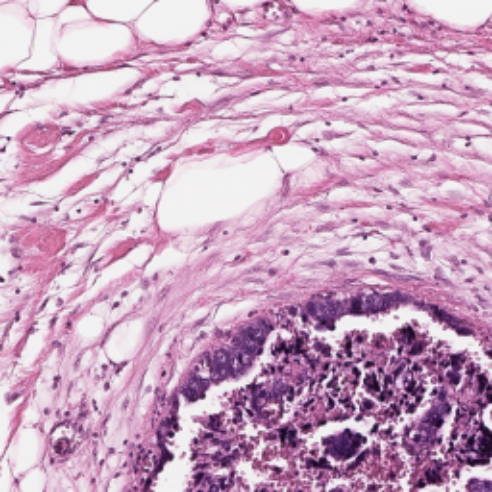

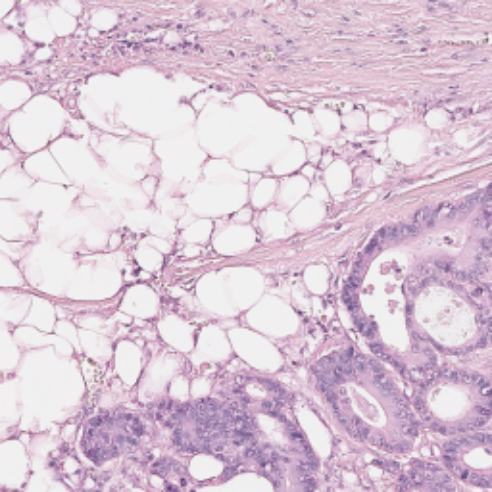

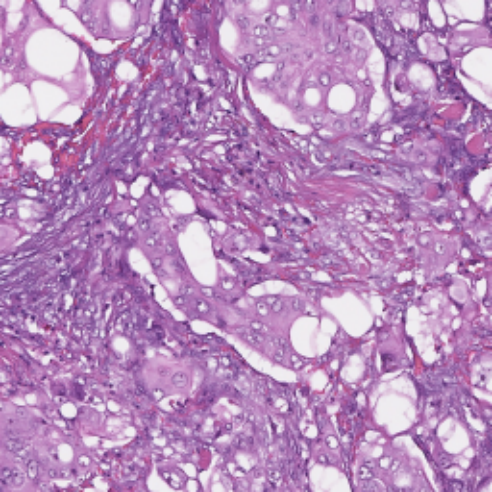

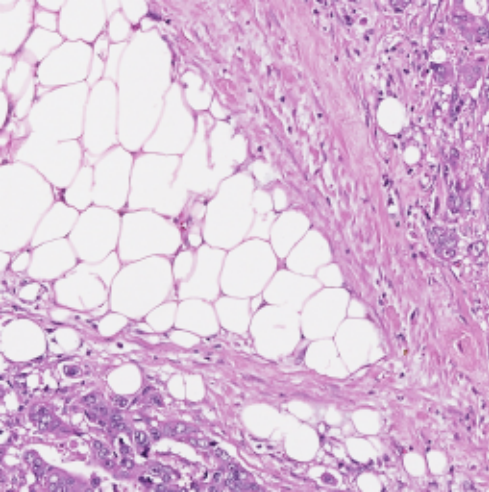

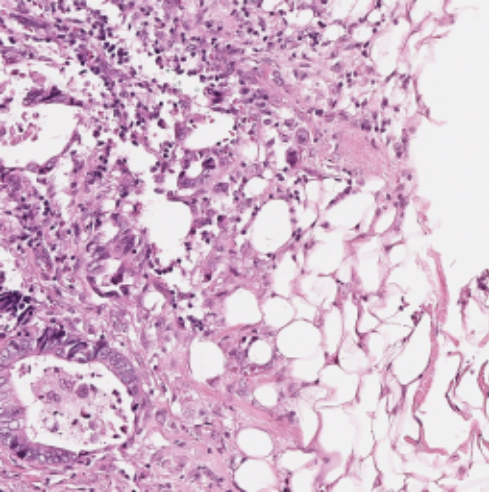

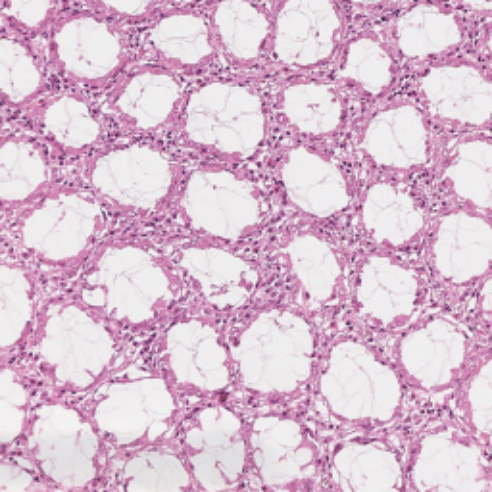

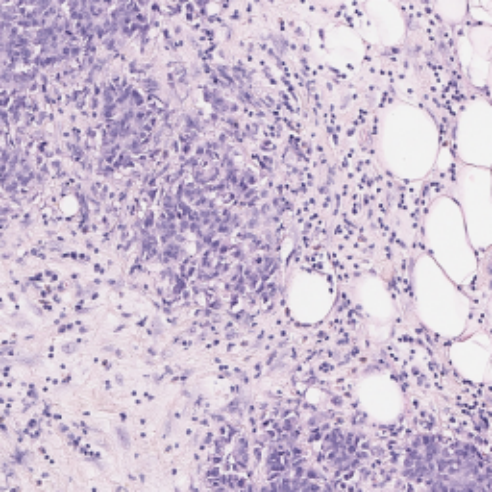

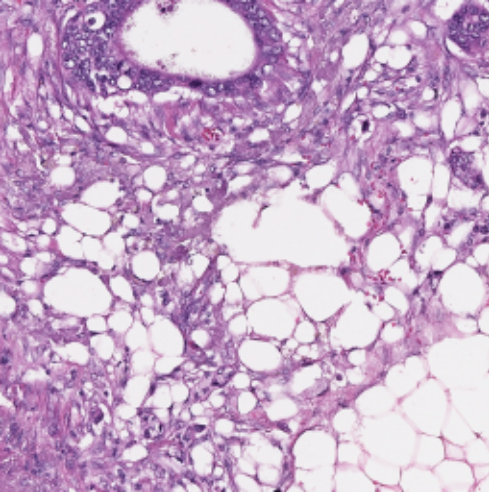

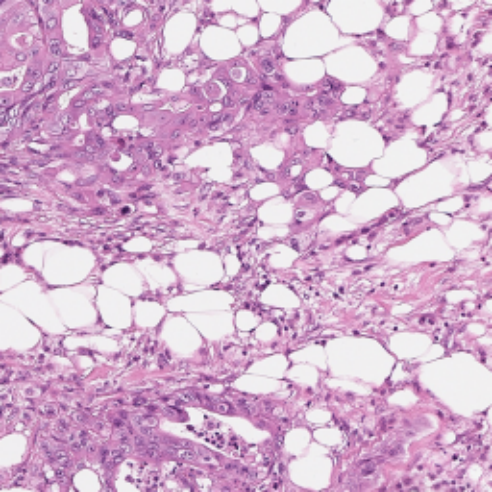

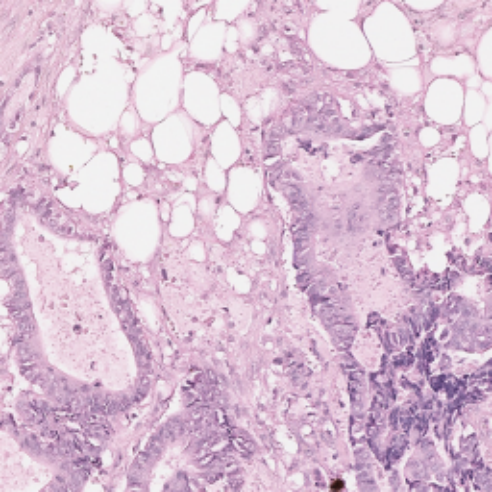

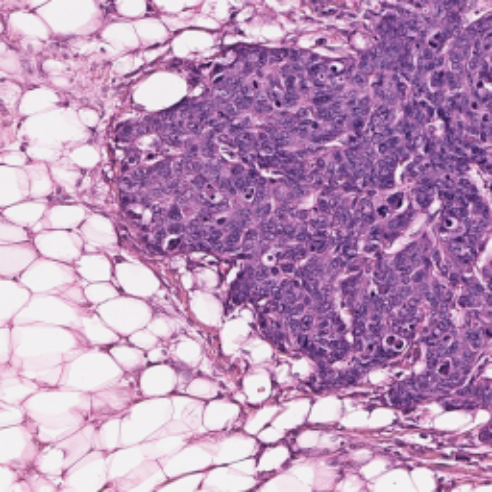

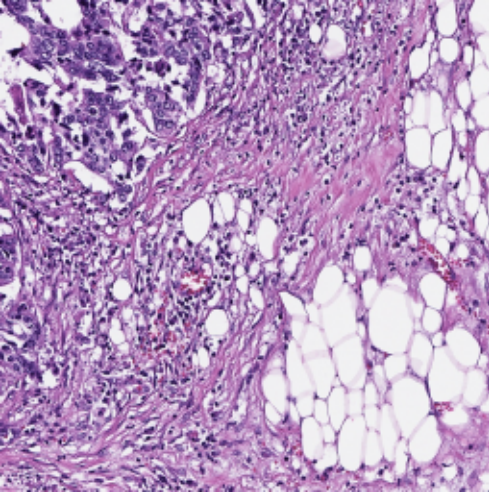

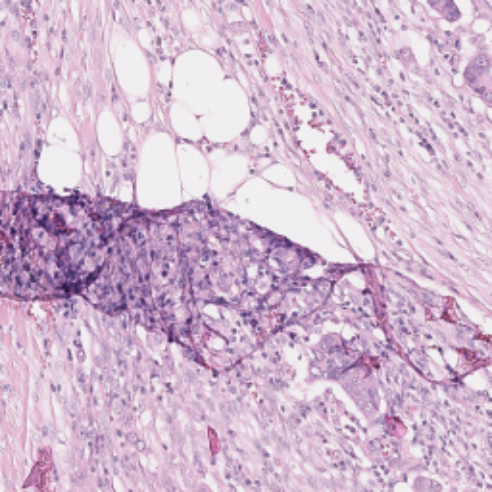

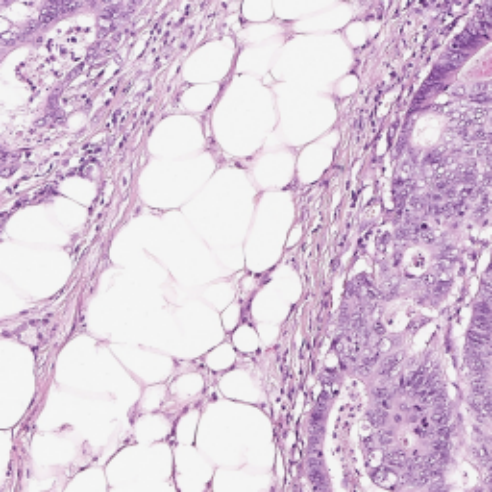

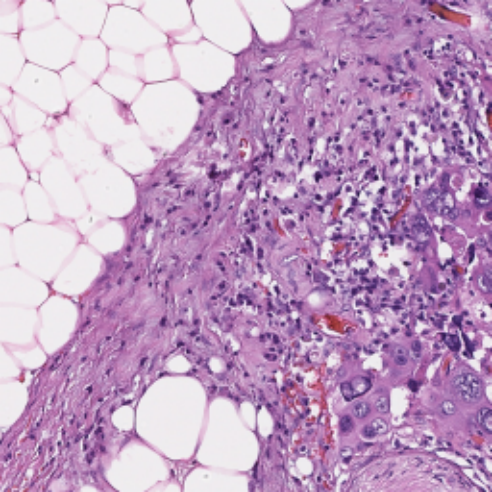

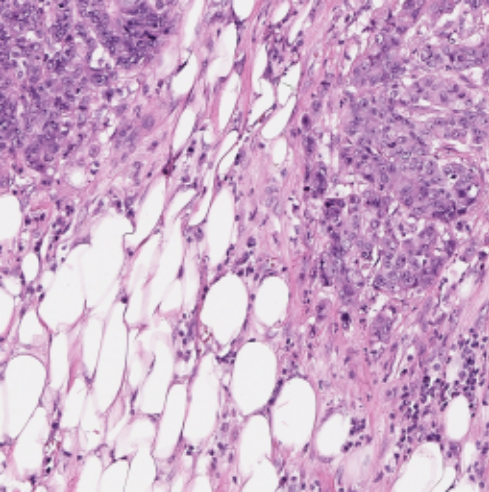

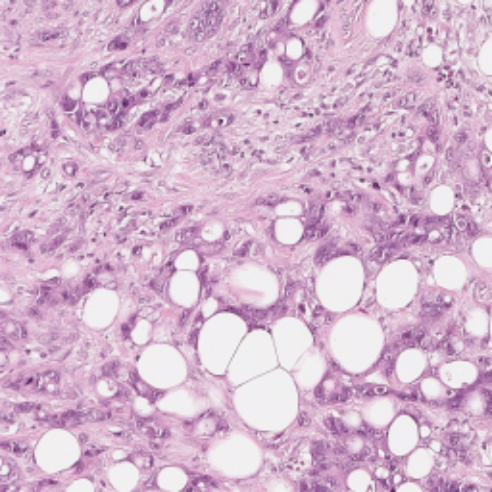

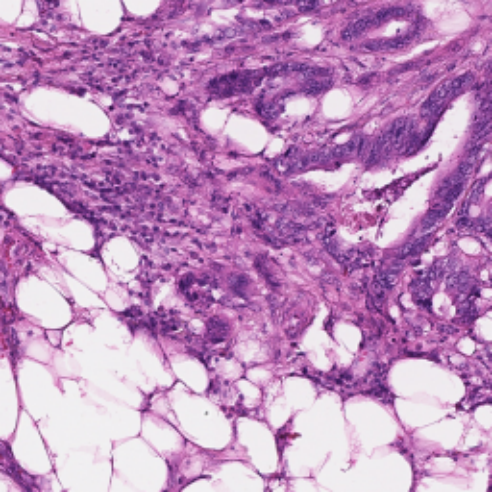

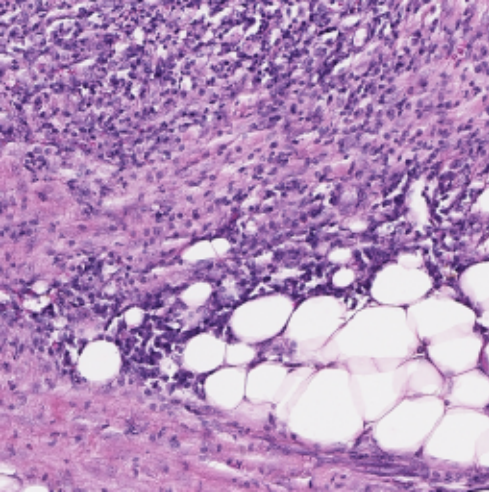

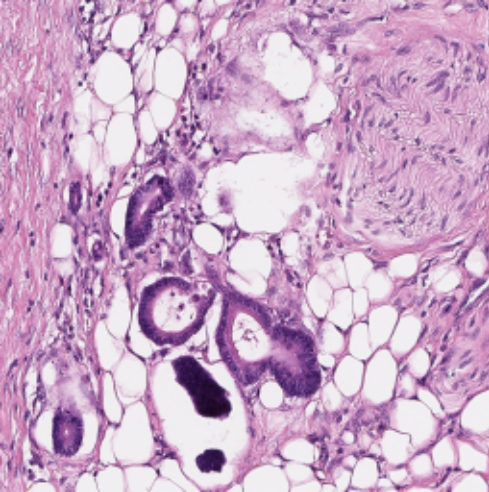

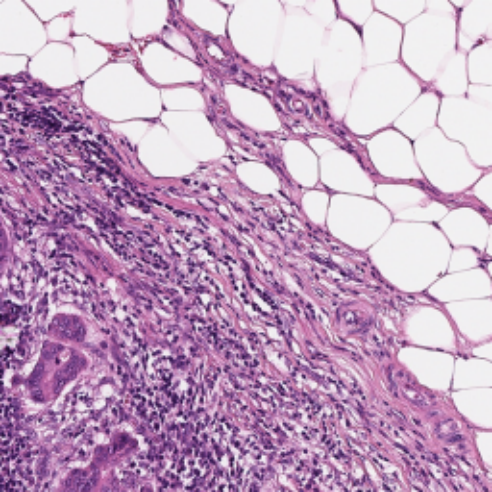

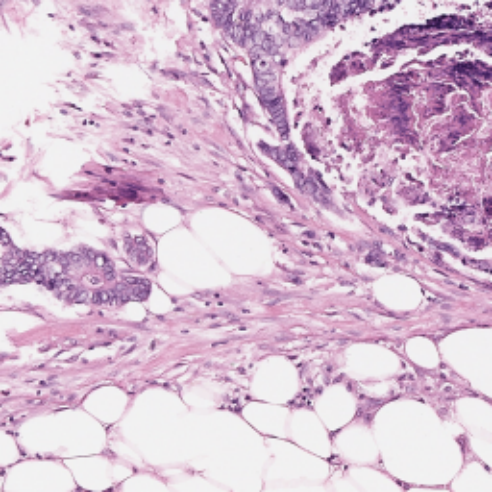

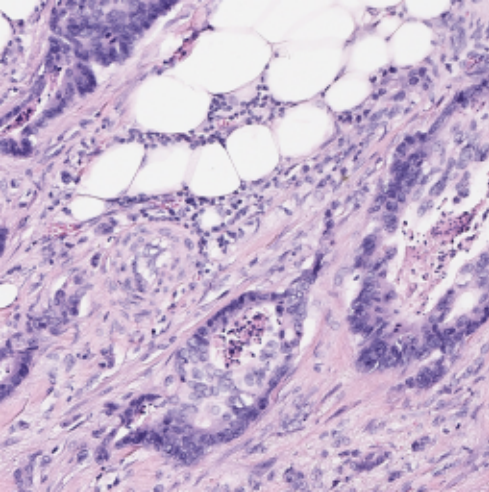

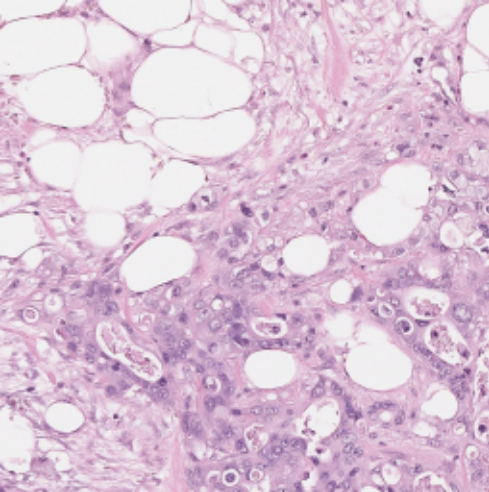

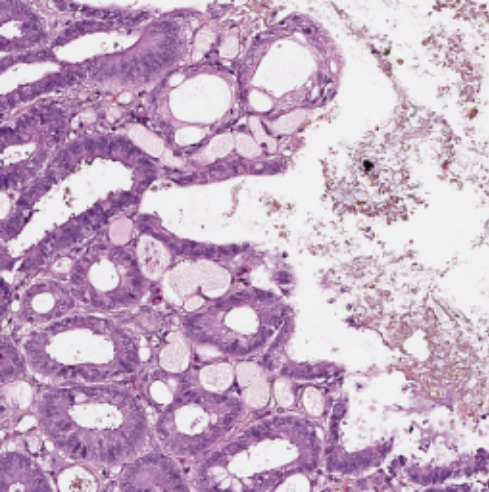

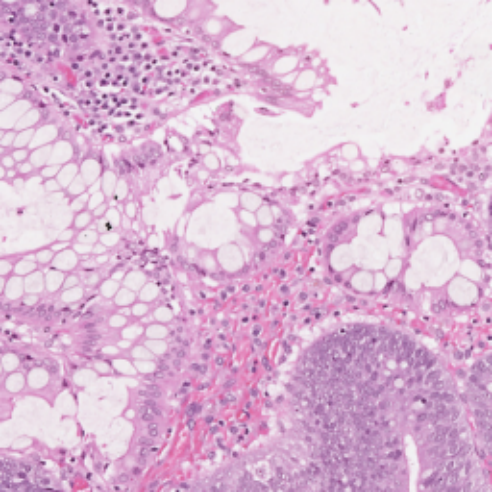

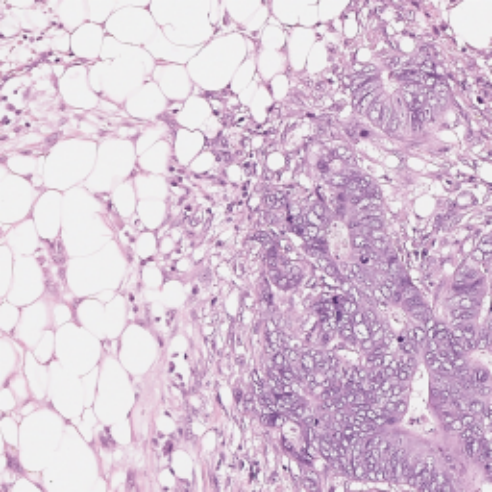

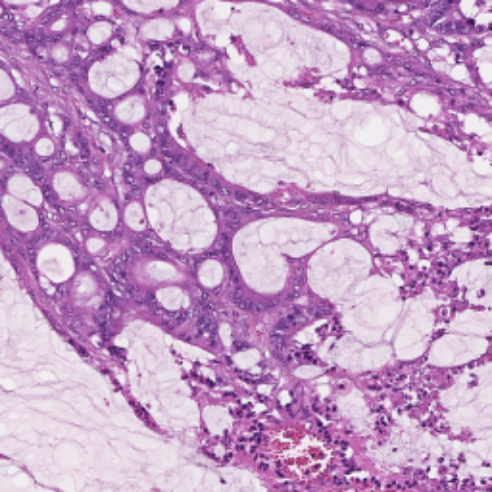

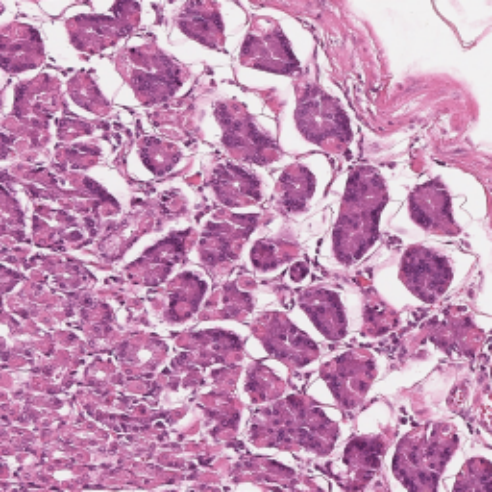

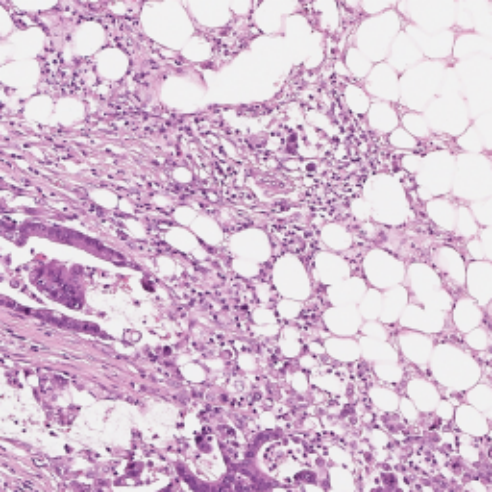

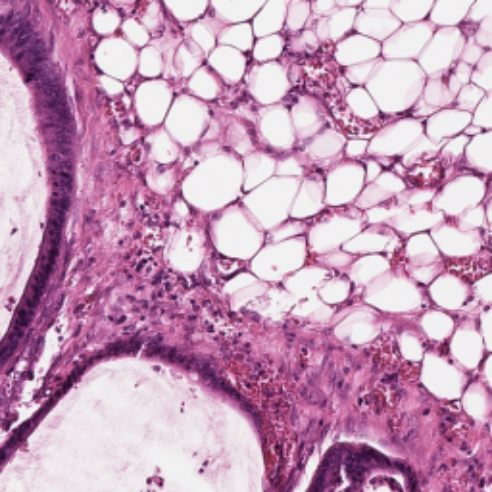

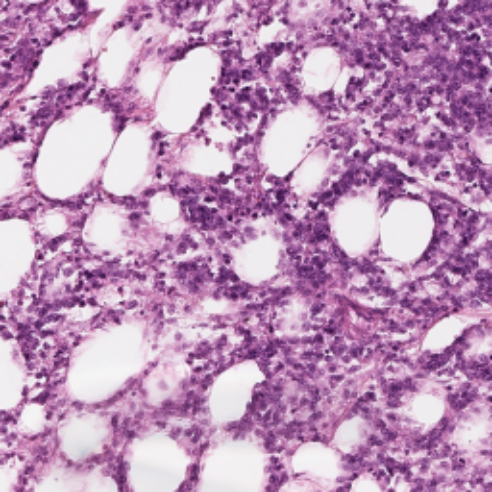

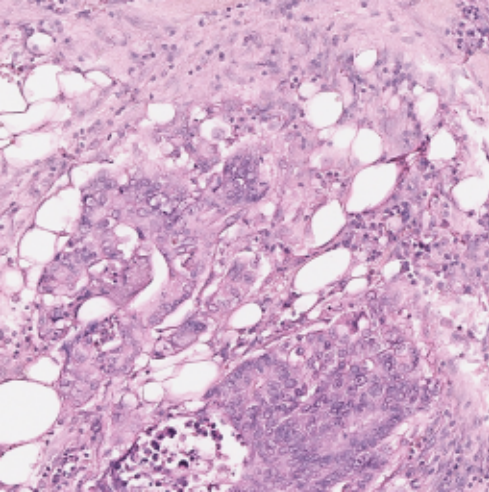

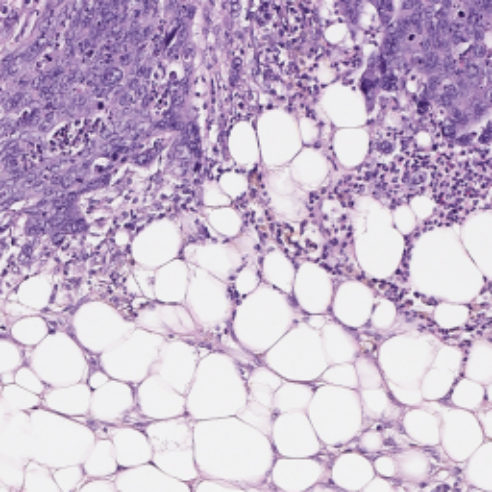

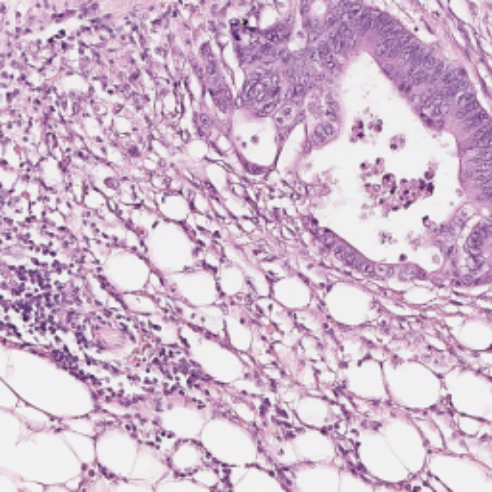

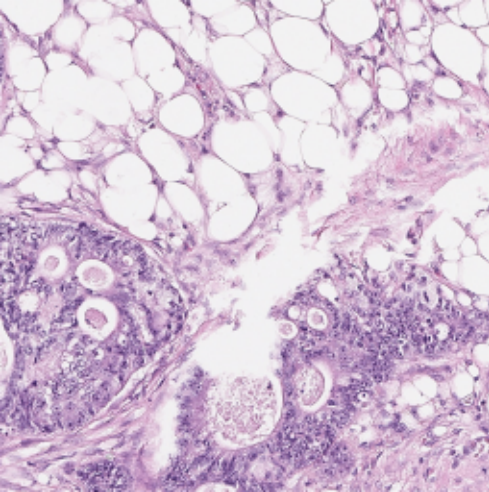

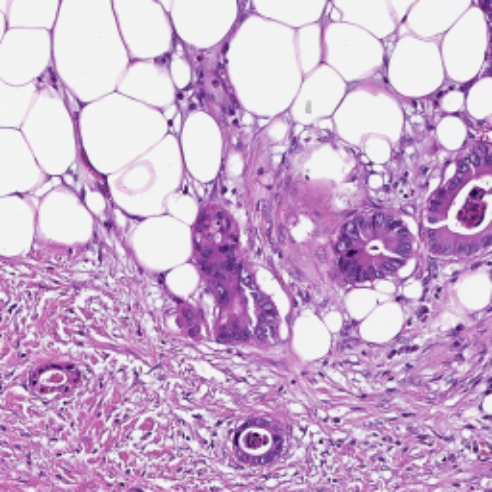

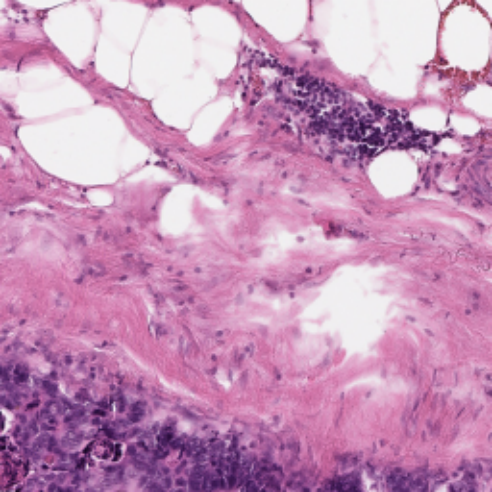

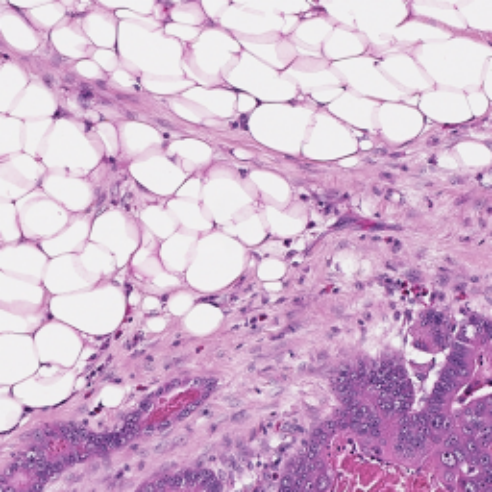

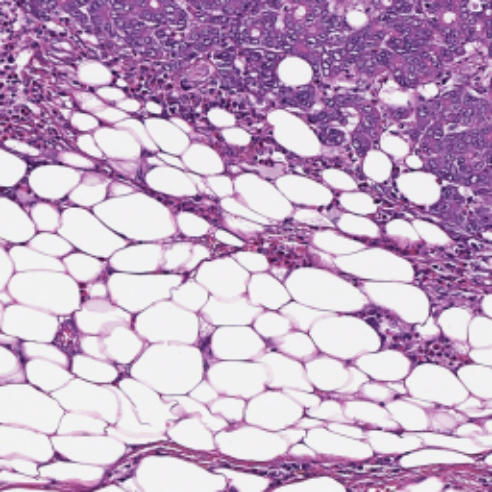

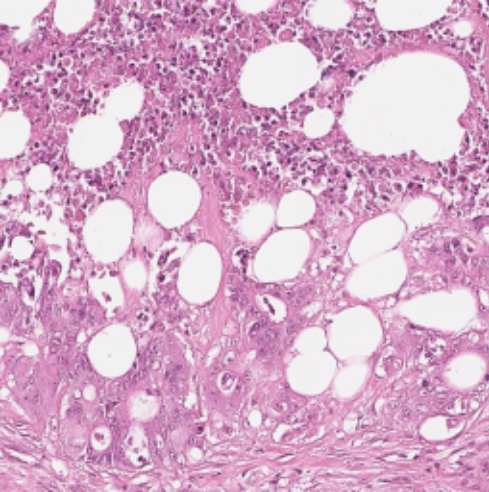

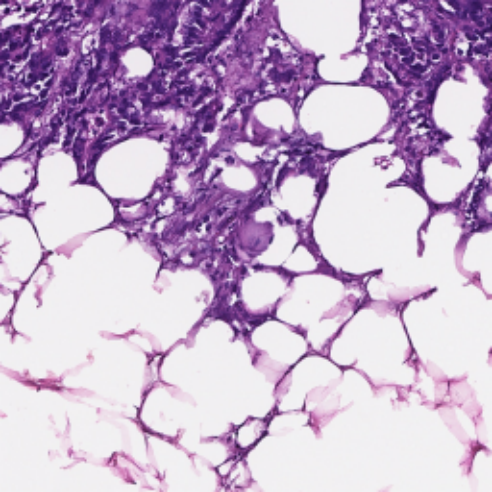

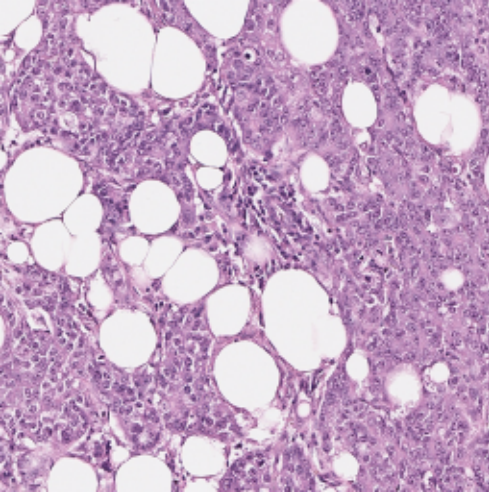

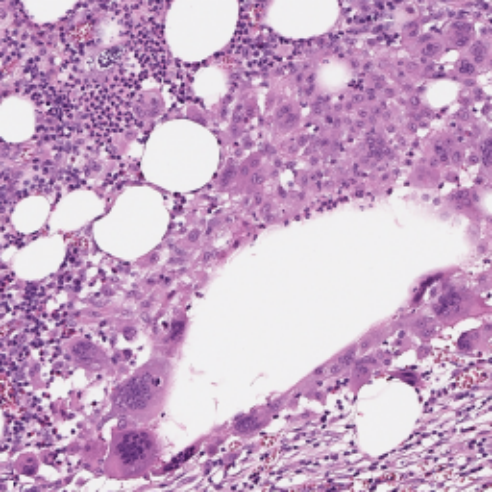

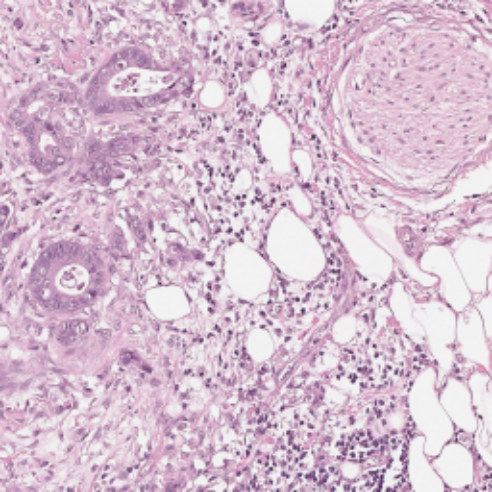

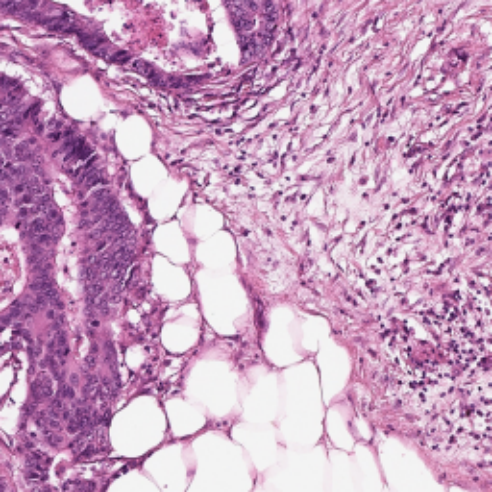

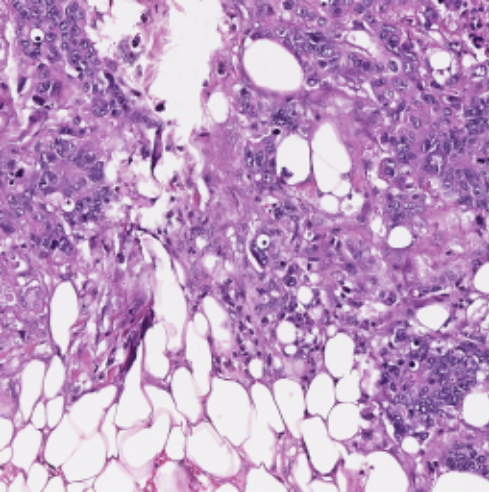

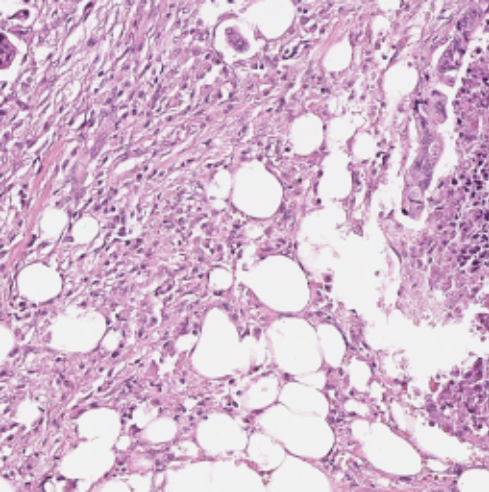

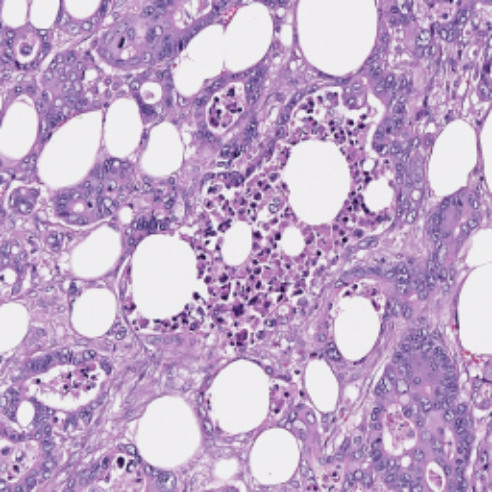

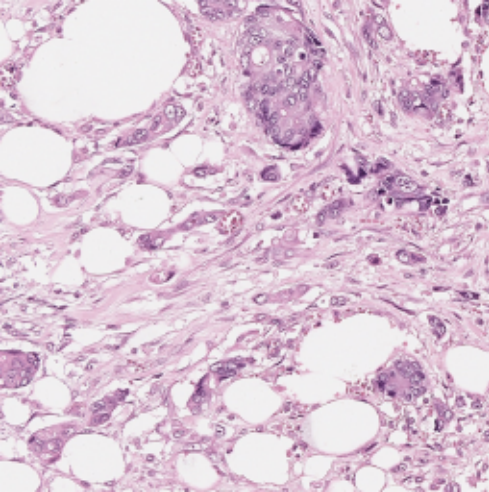

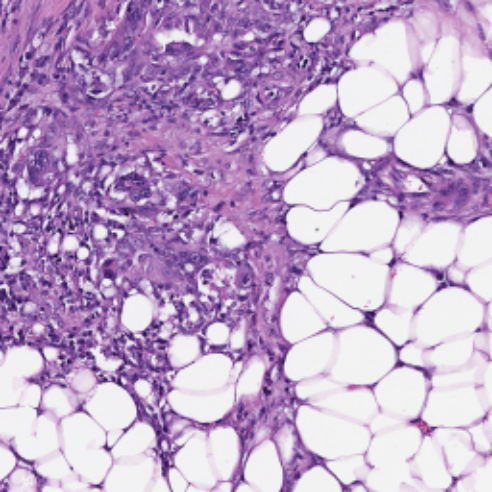

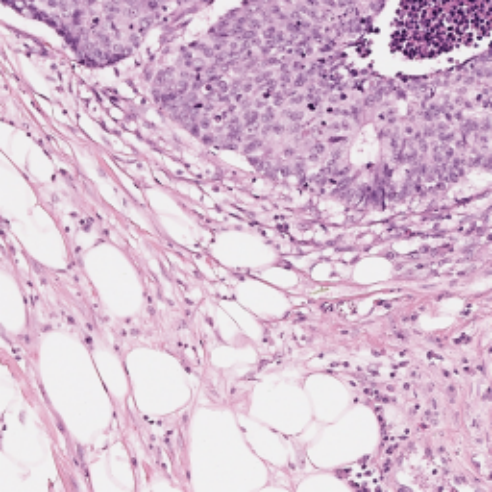

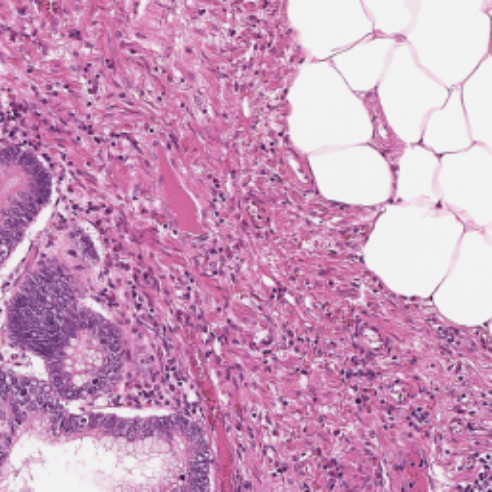

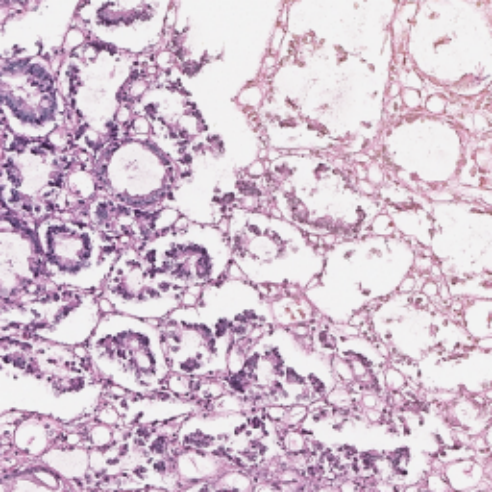

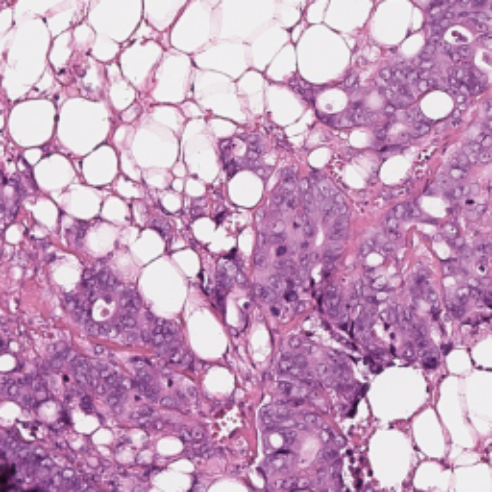

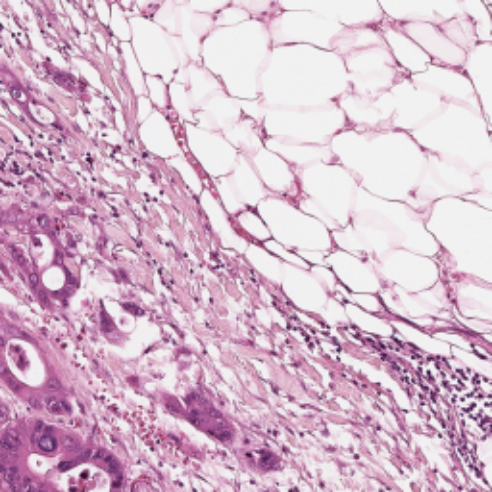

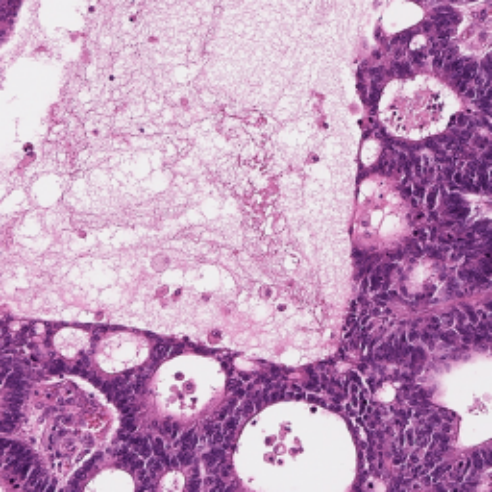

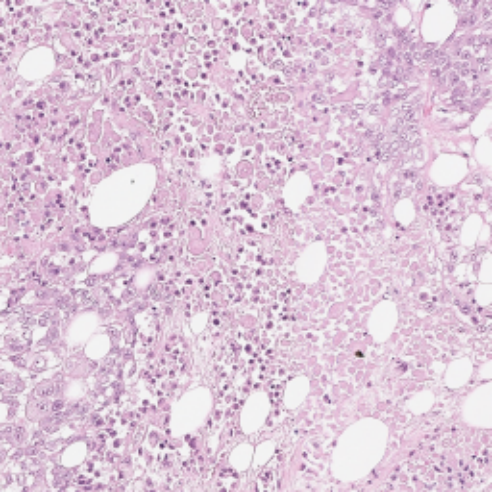

Supplement: Supplementary file 3 — Supplementary Data 3 [file 41746_2021_427_MOESM3_ESM.zip › assessment_patches_TAF.pdf]

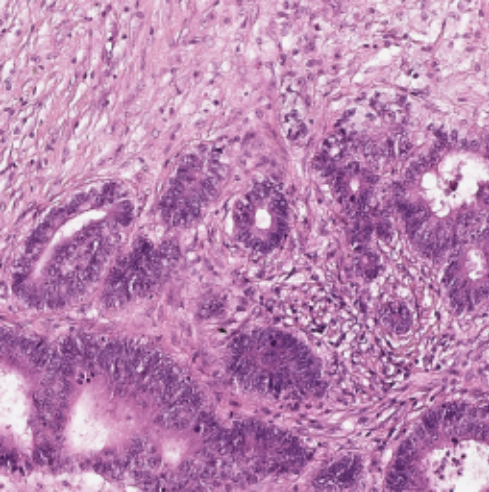

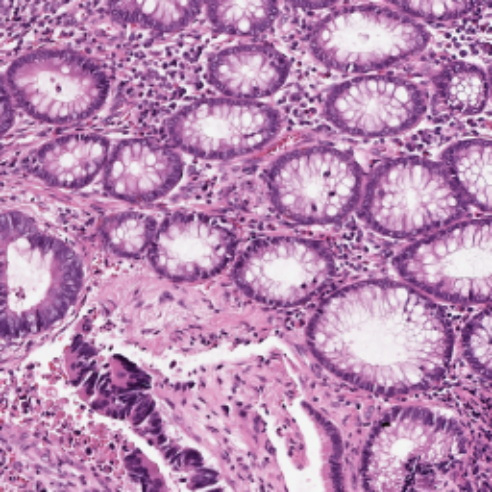

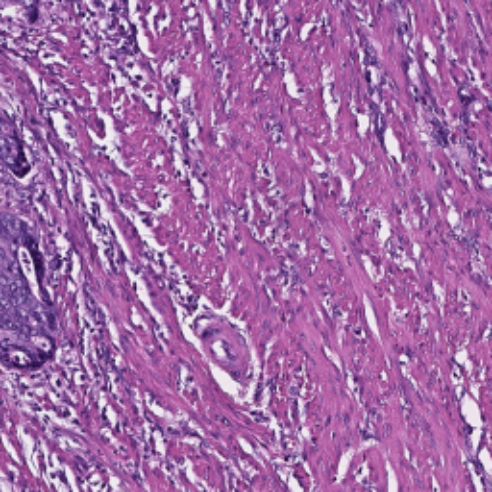

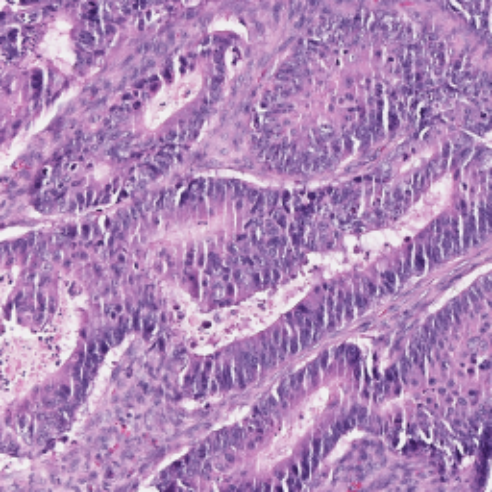

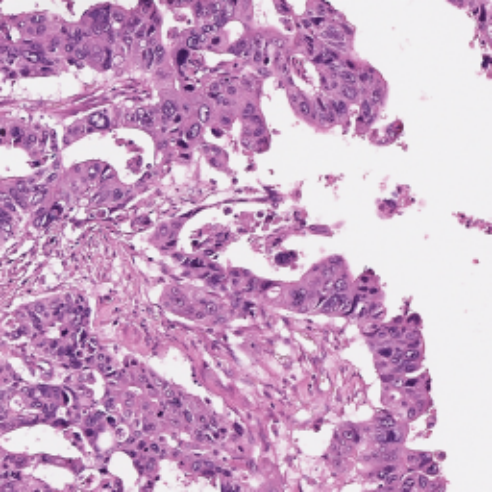

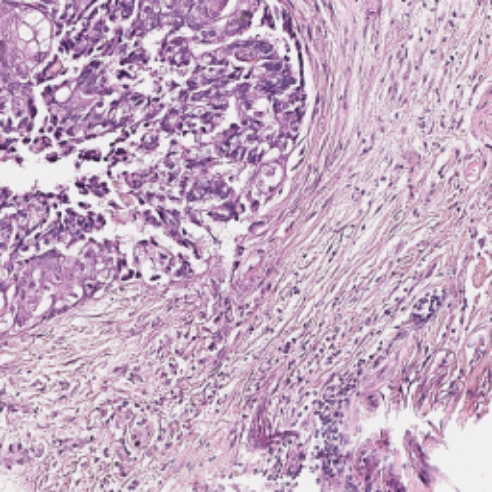

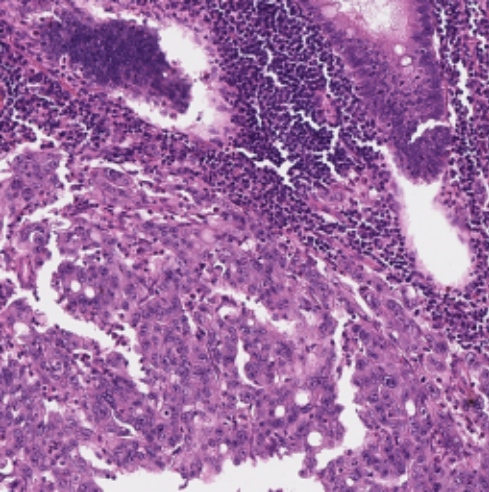

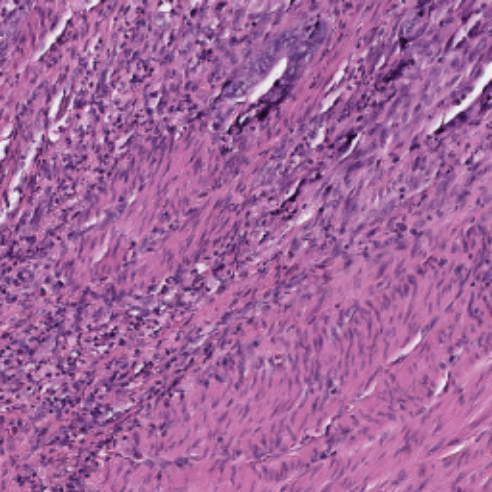

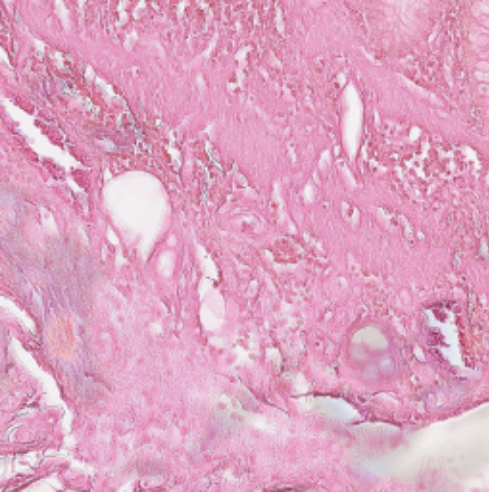

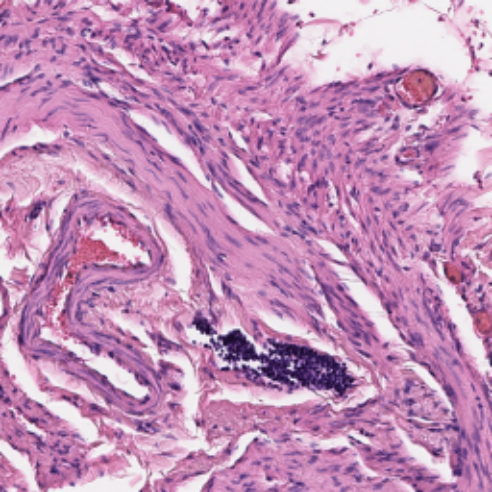

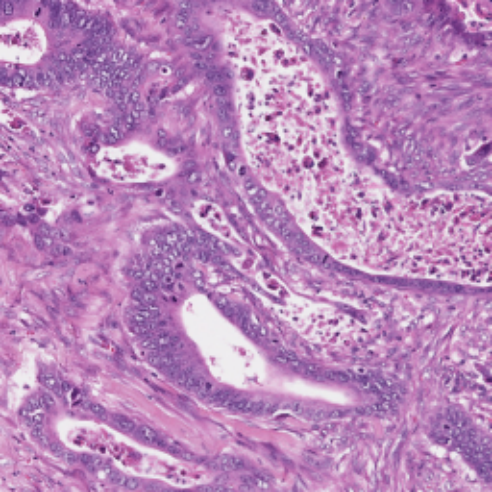

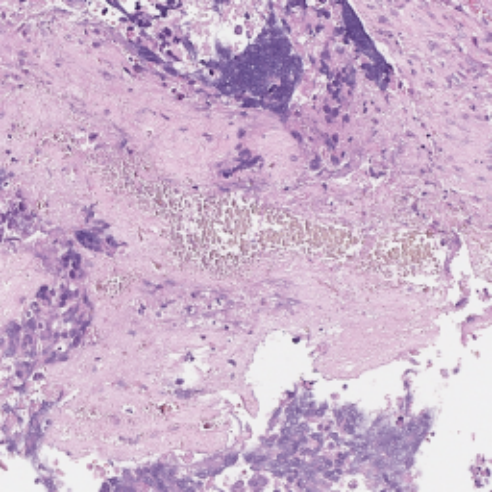

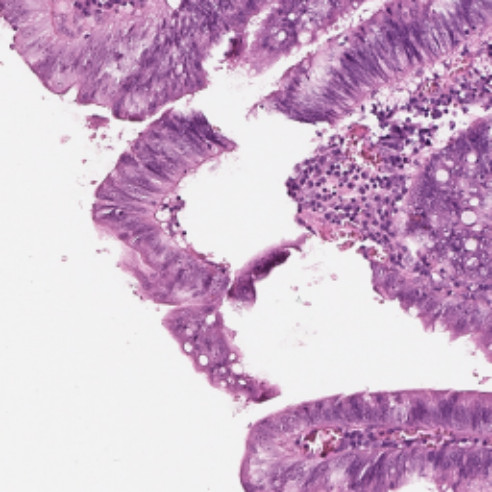

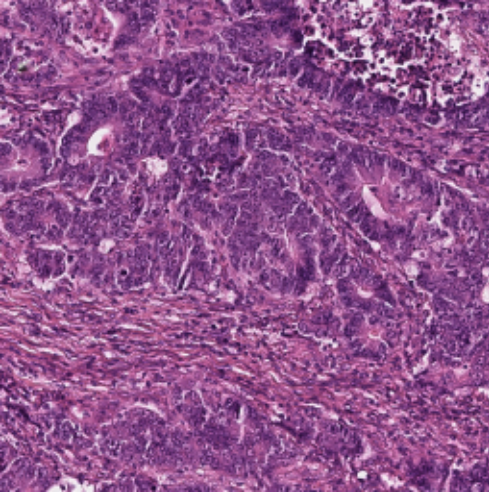

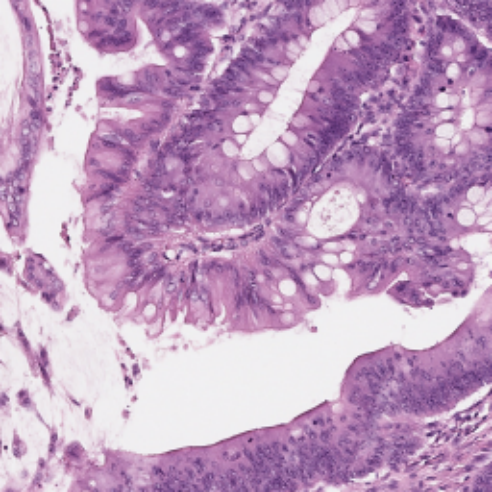

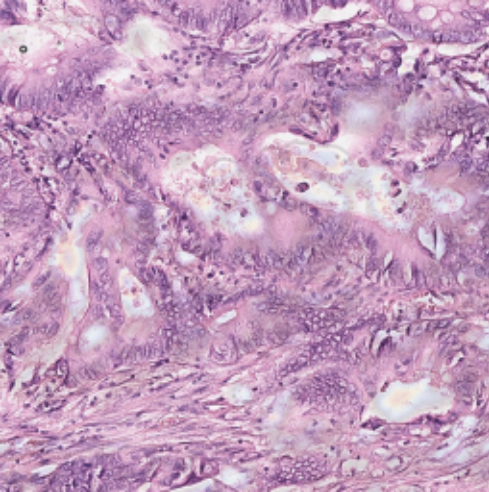

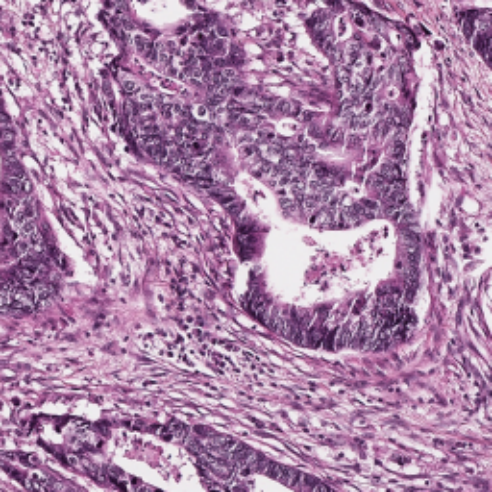

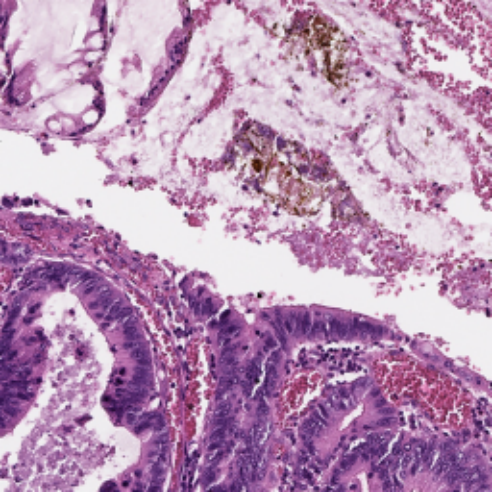

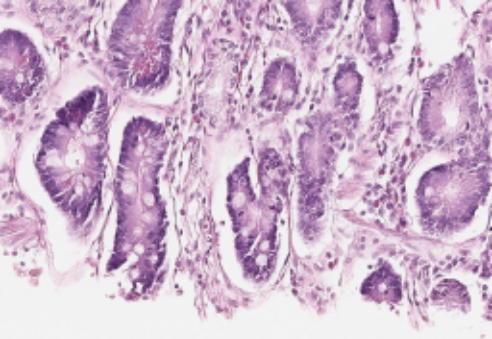

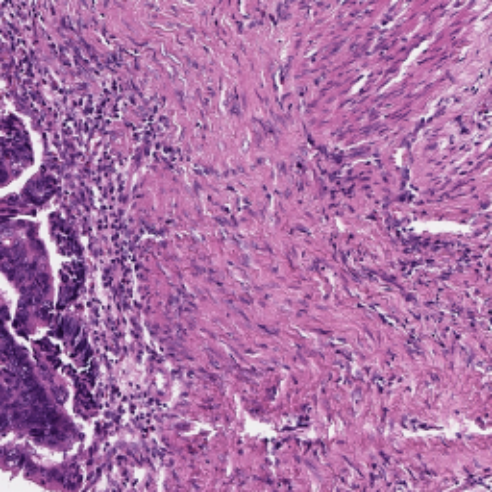

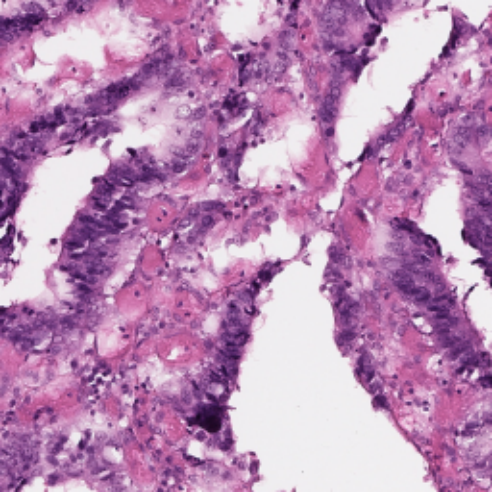

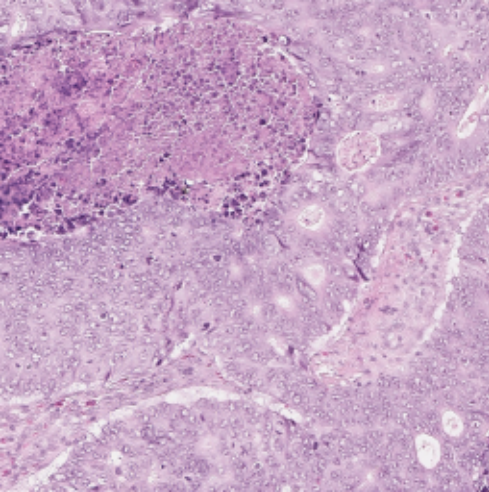

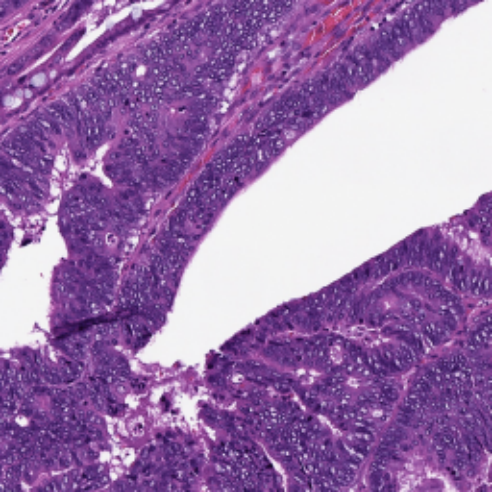

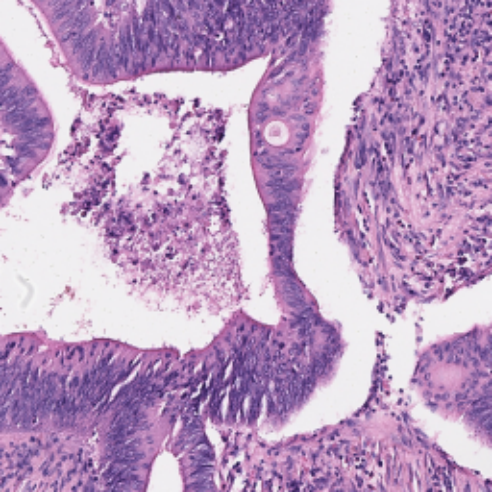

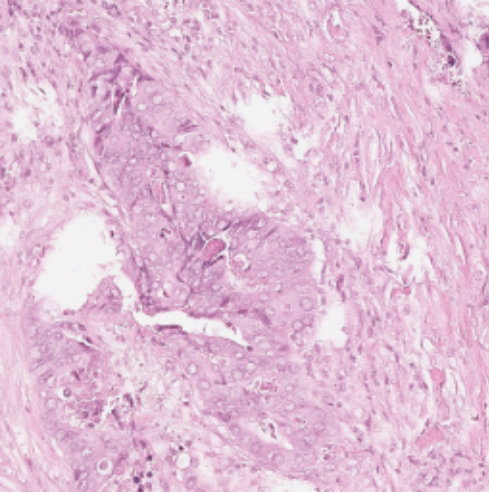

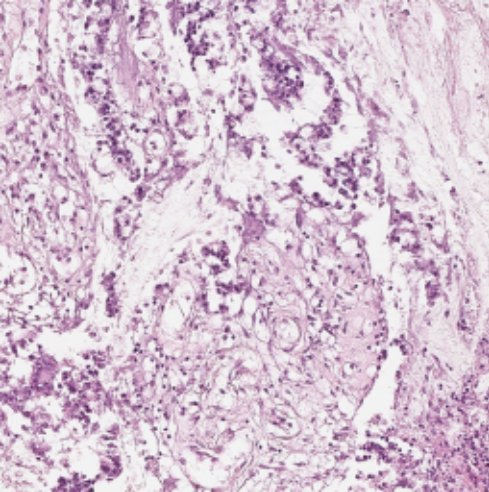

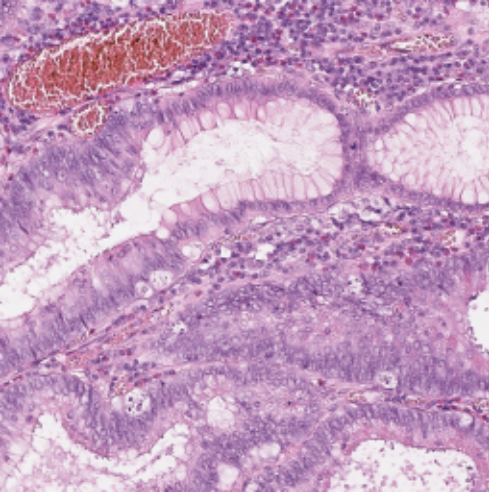

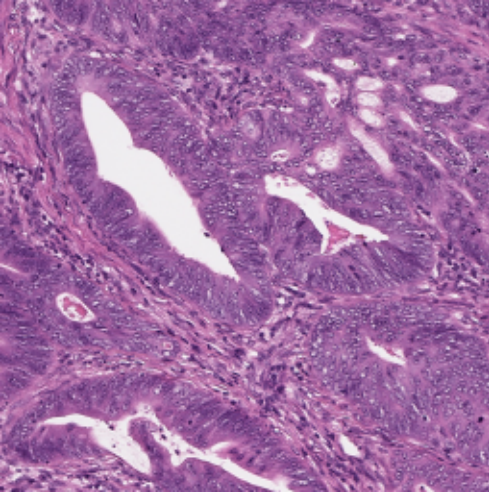

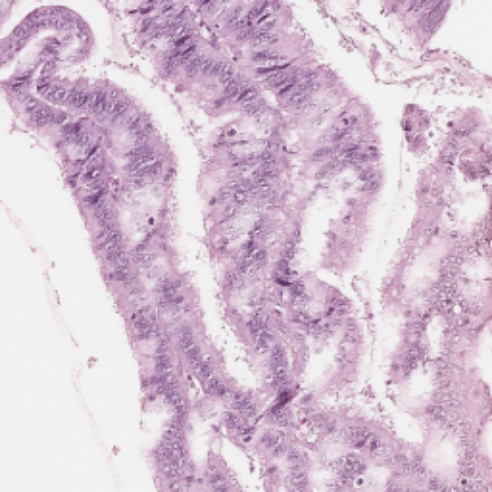

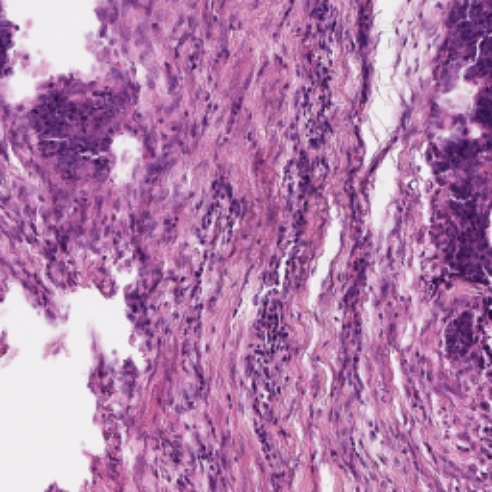

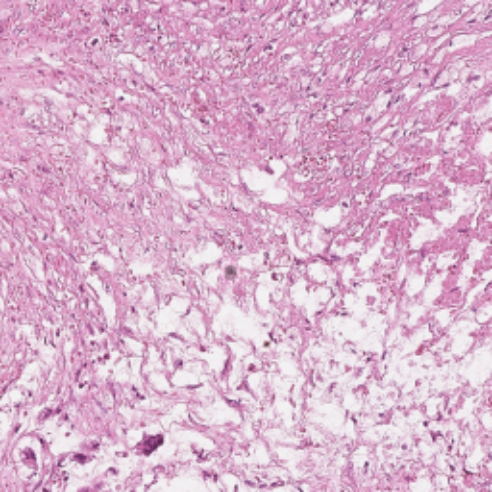

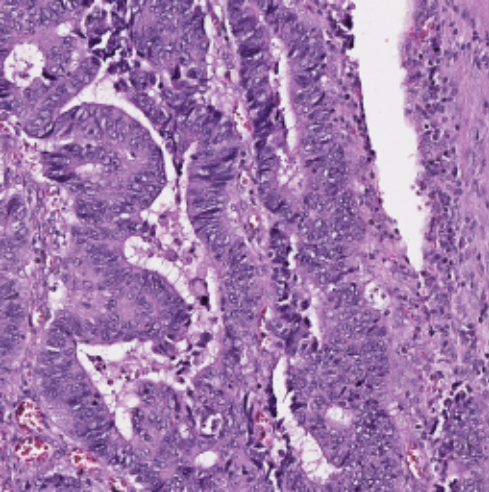

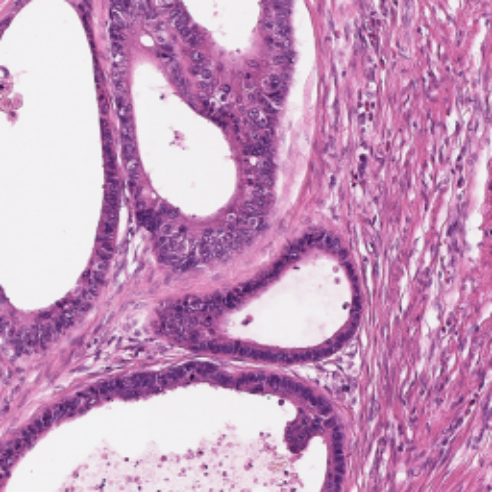

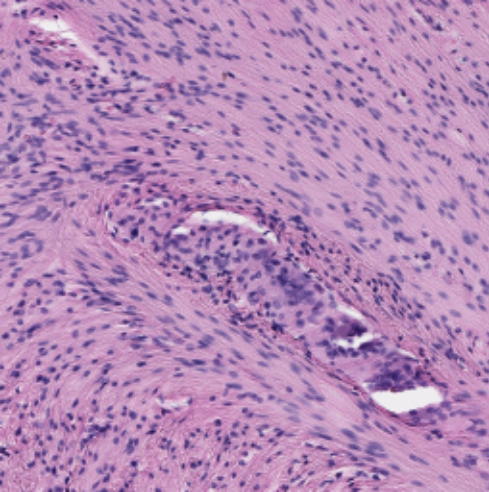

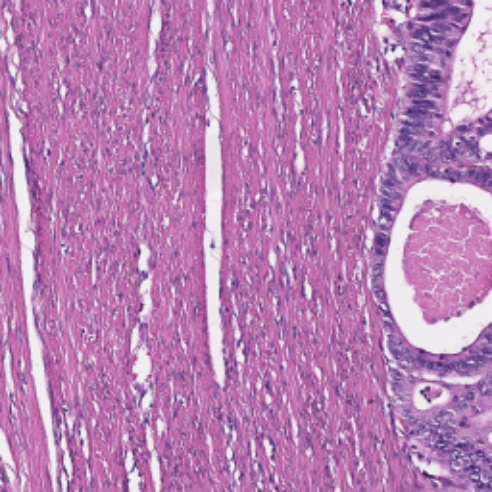

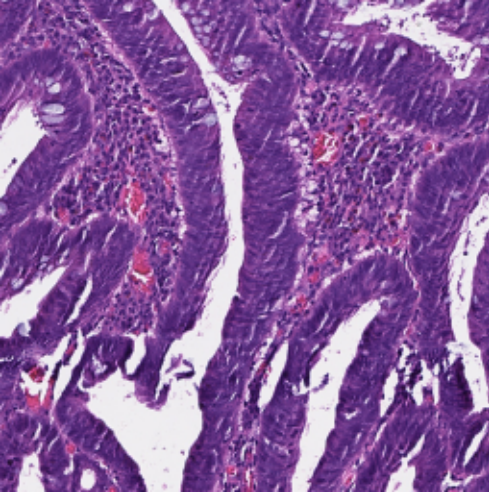

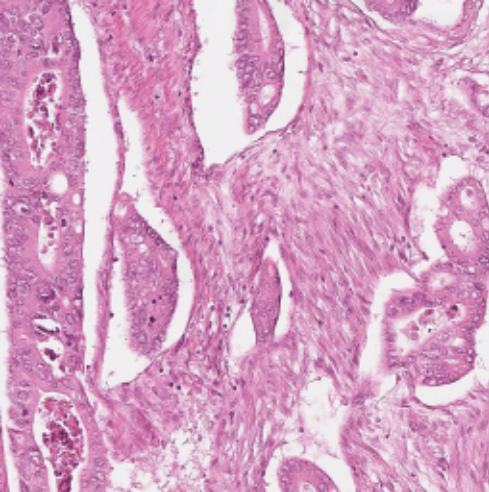

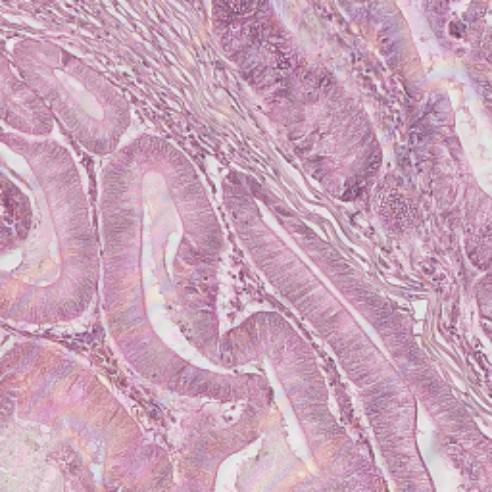

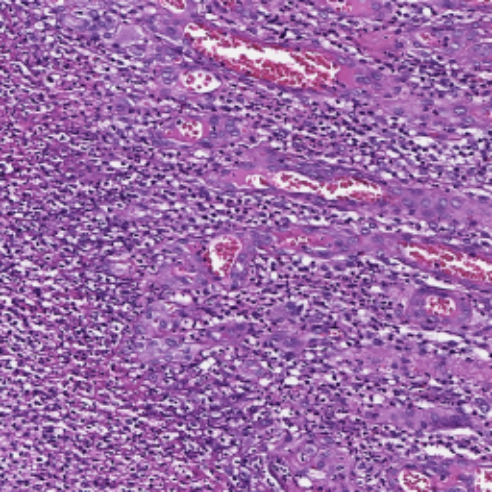

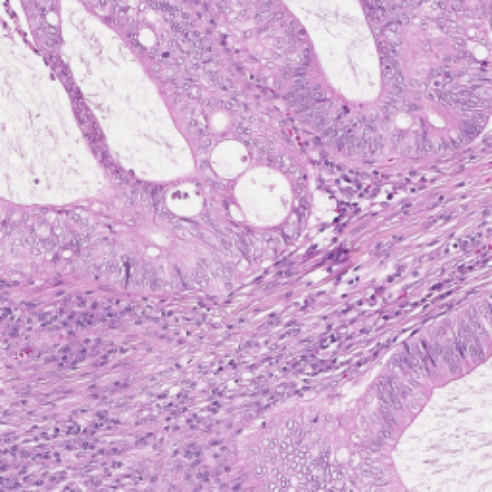

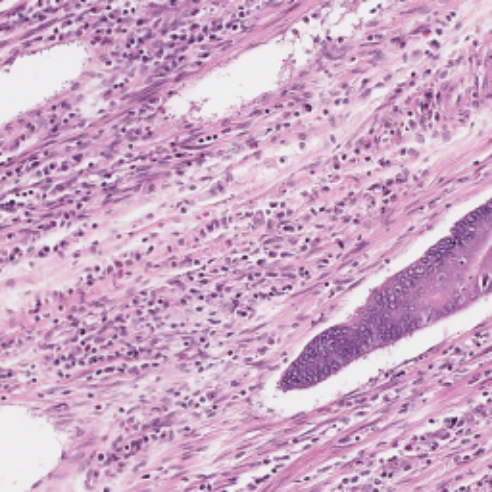

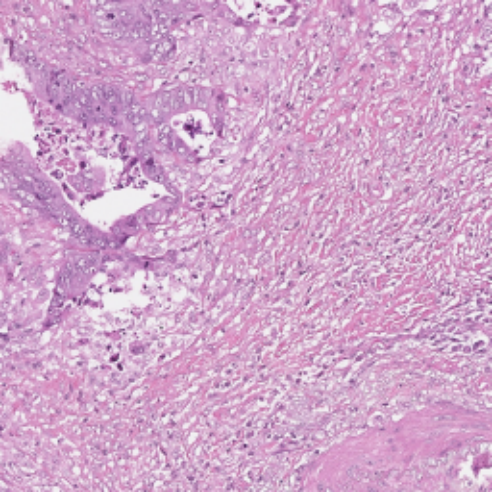

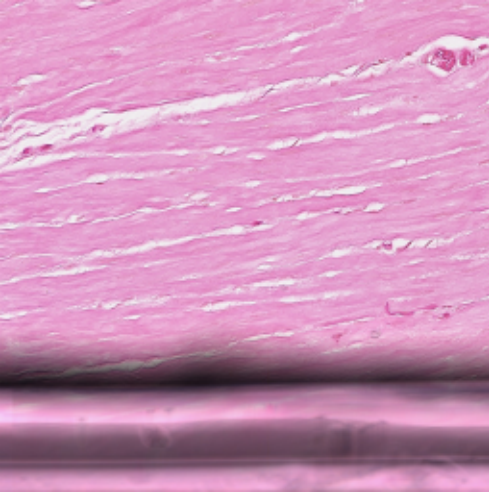

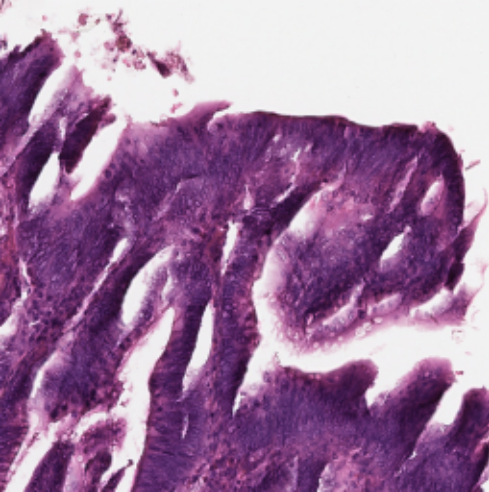

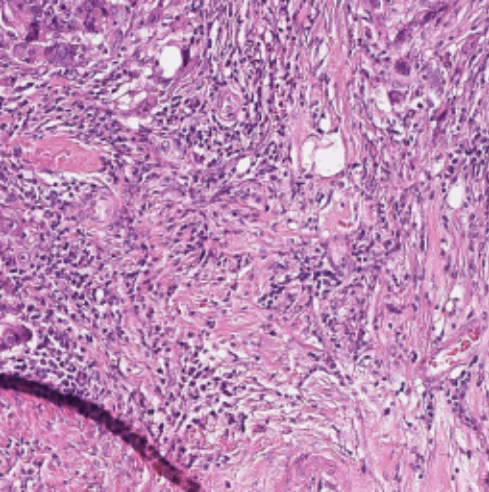

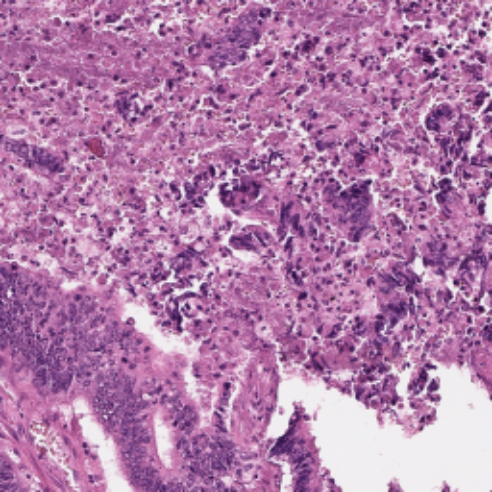

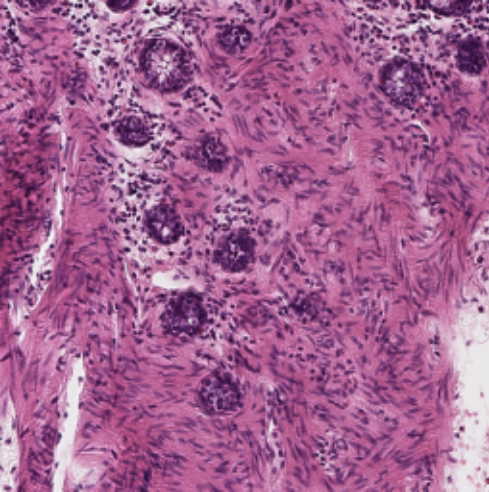

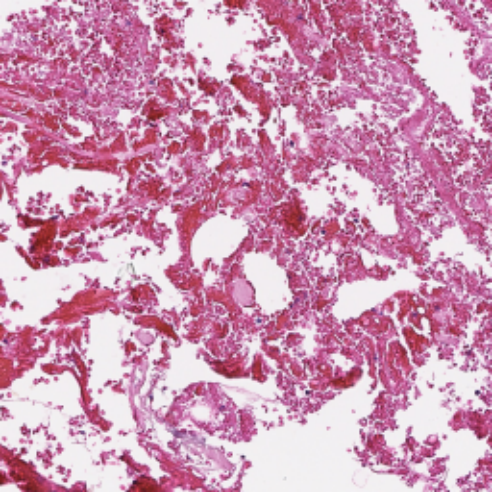

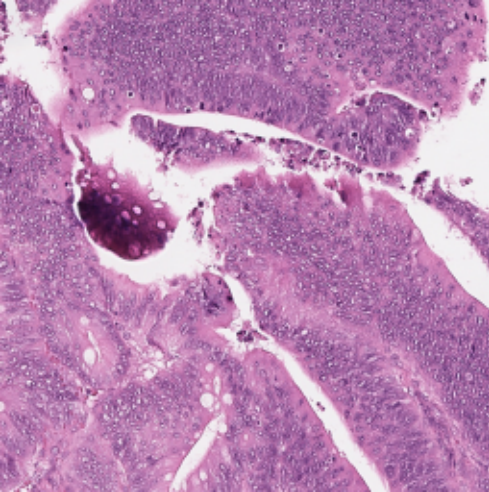

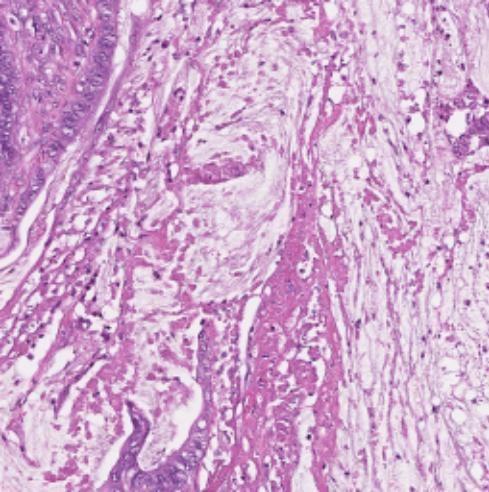

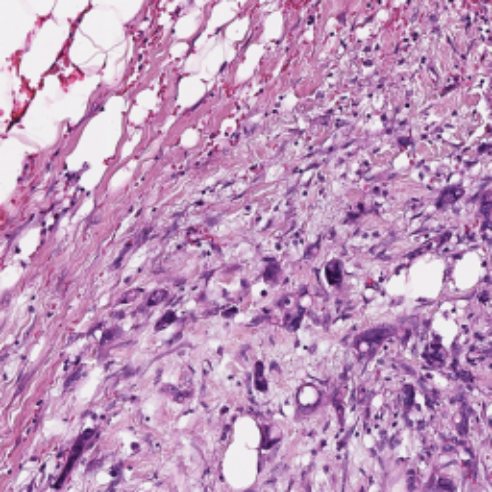

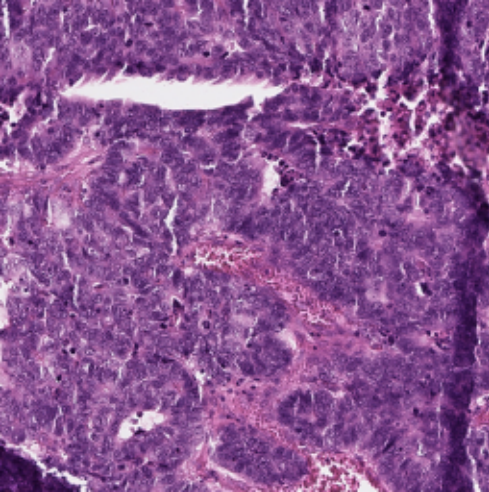

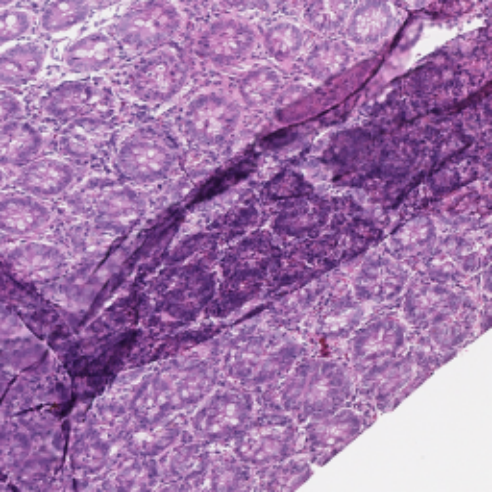

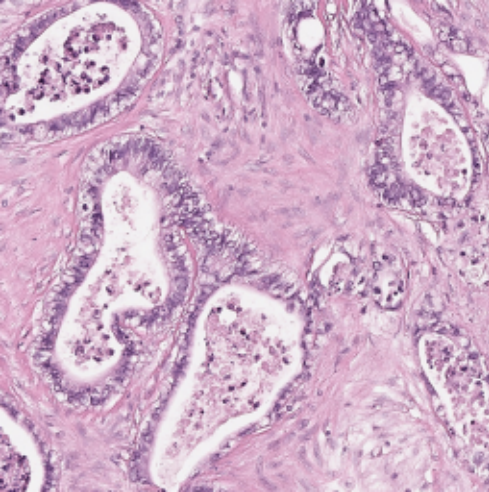

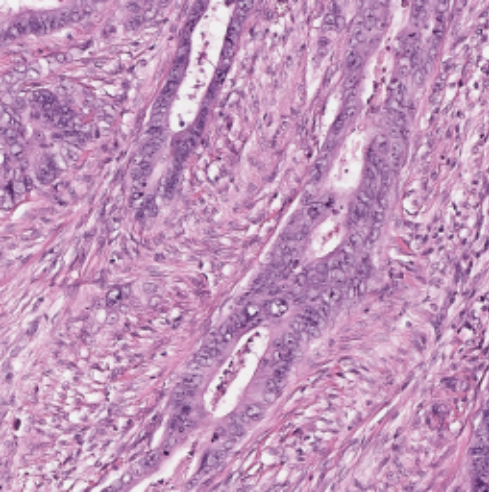

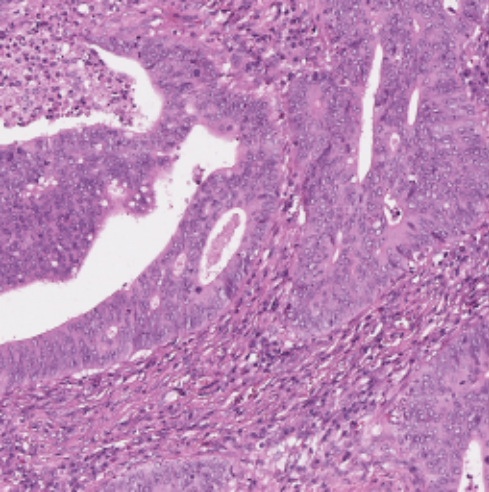

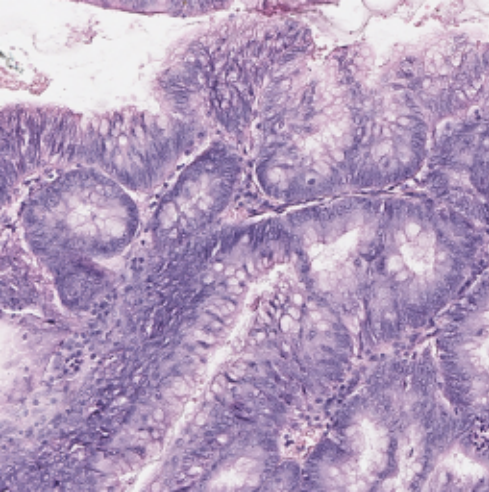

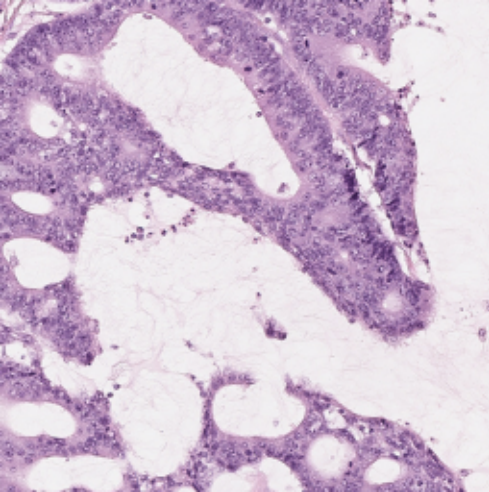

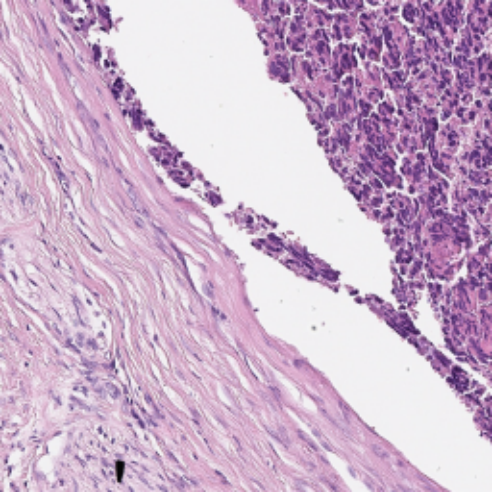

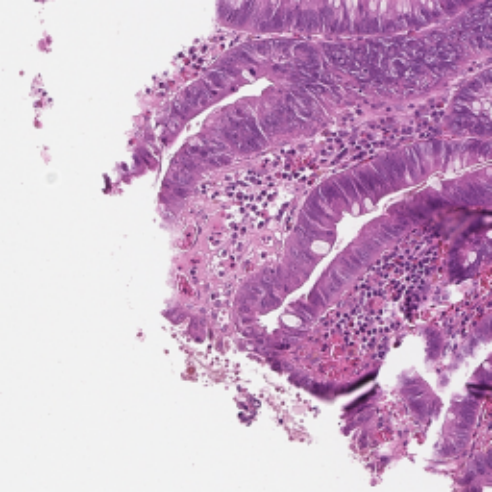

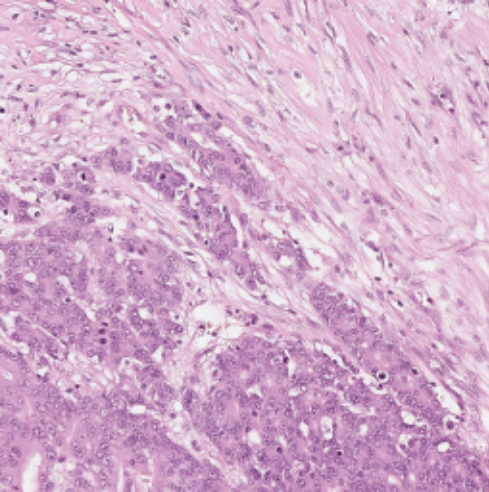

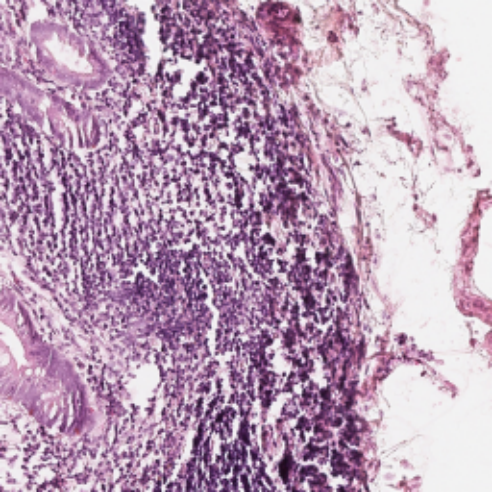

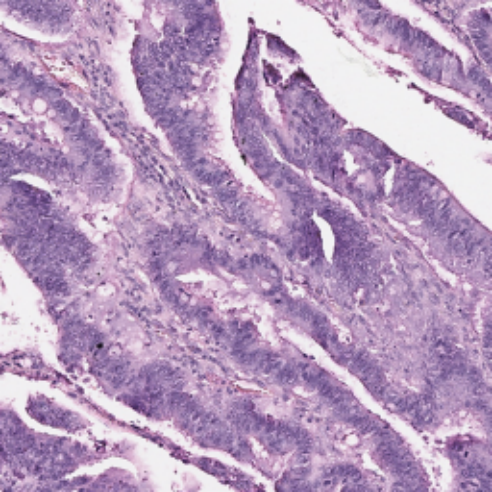

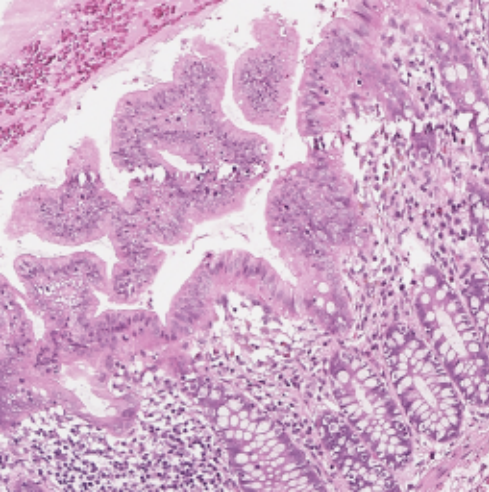

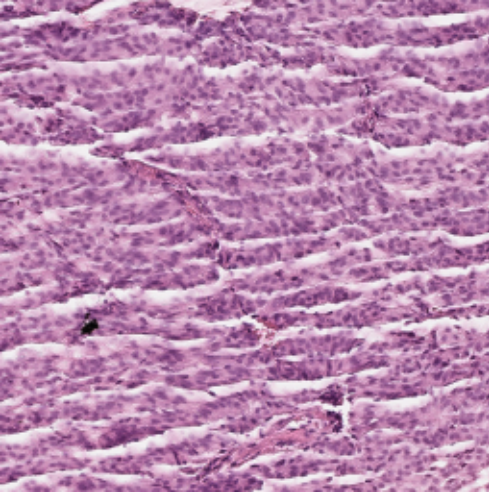

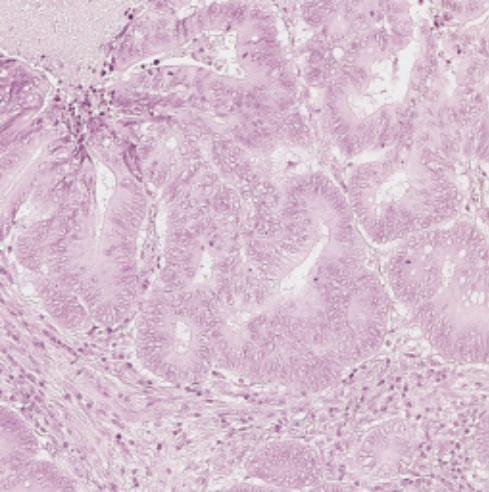

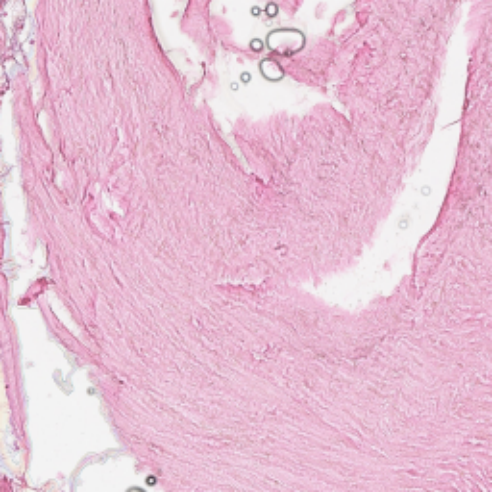

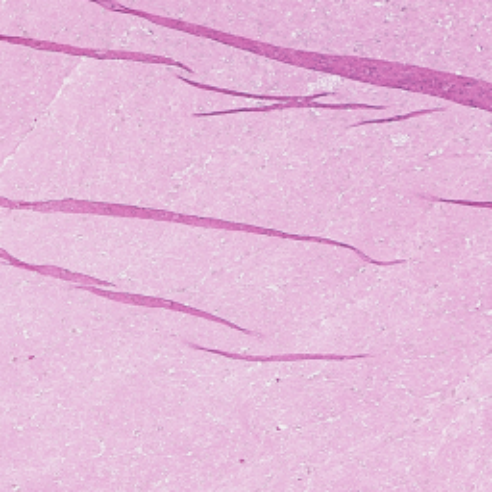

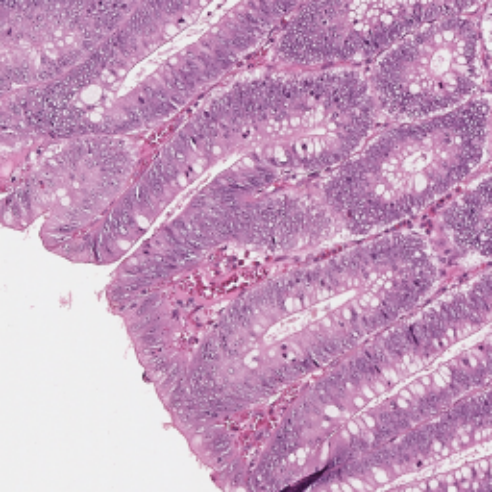

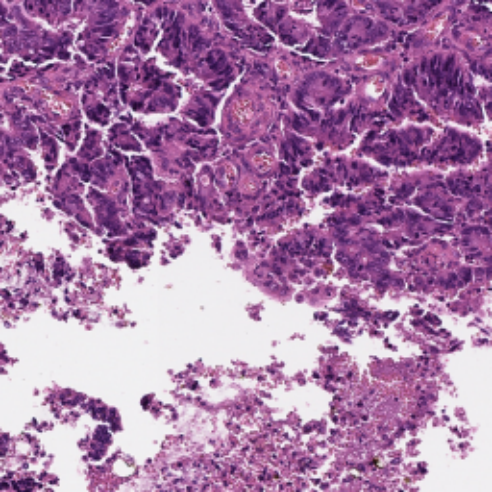

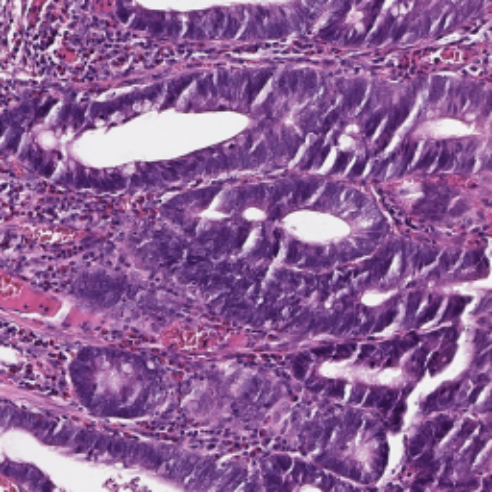

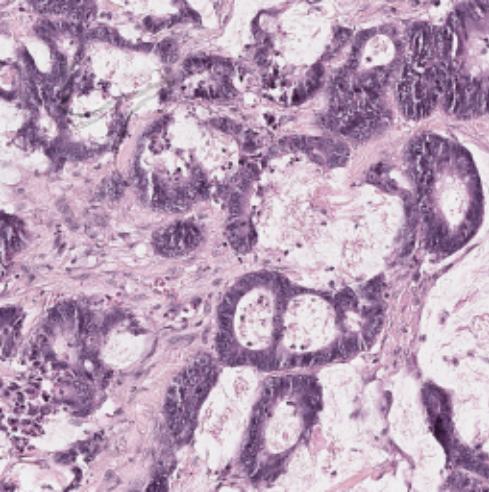

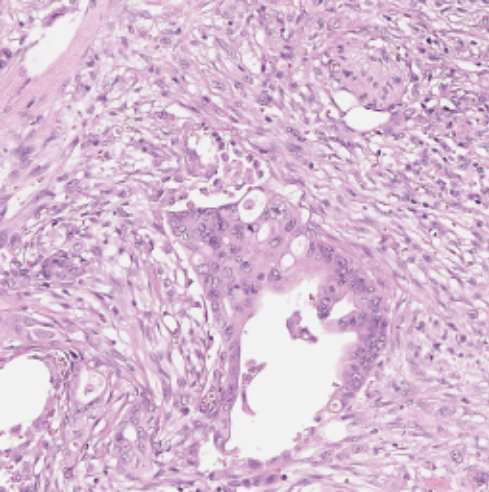

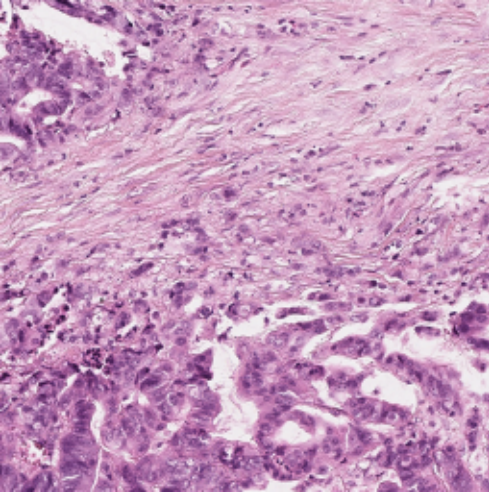

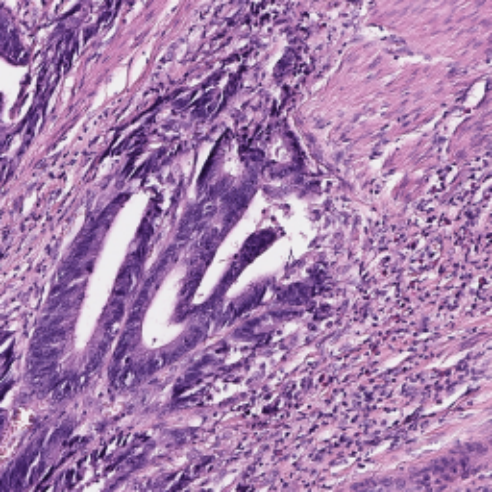

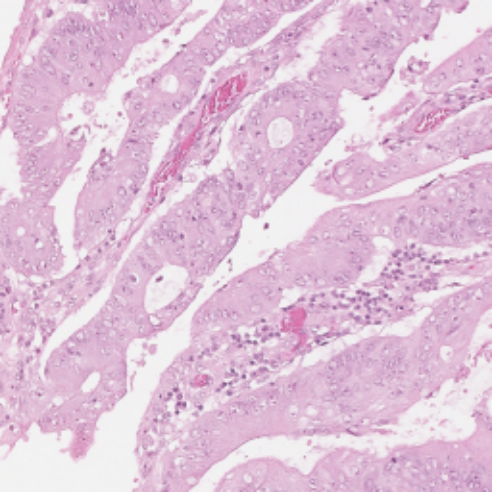

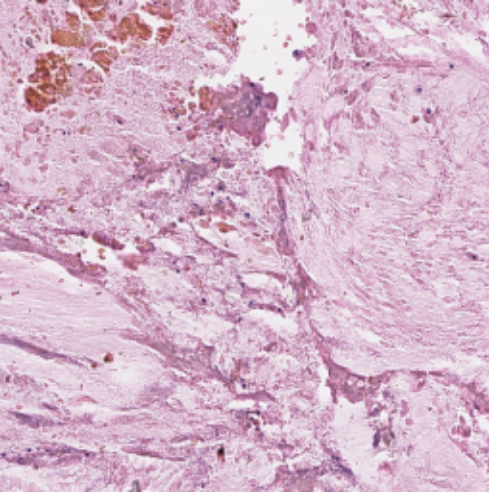

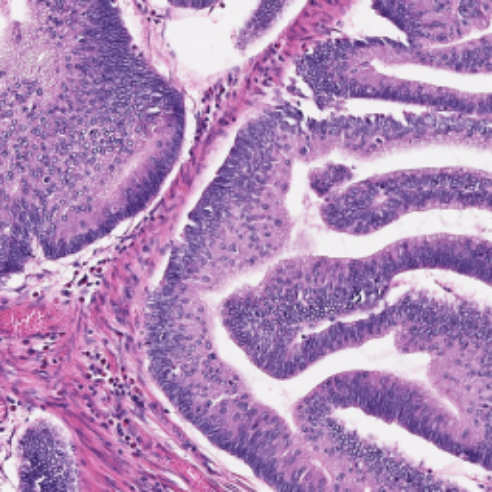

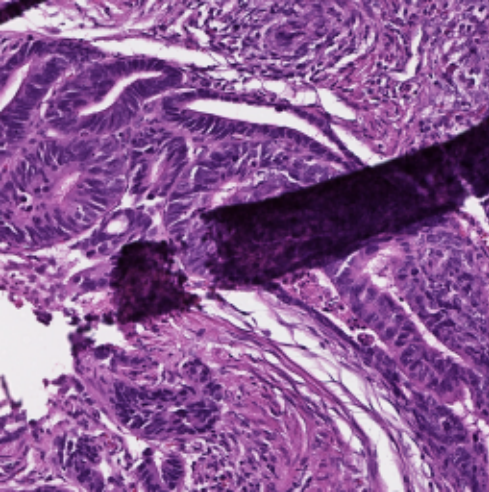

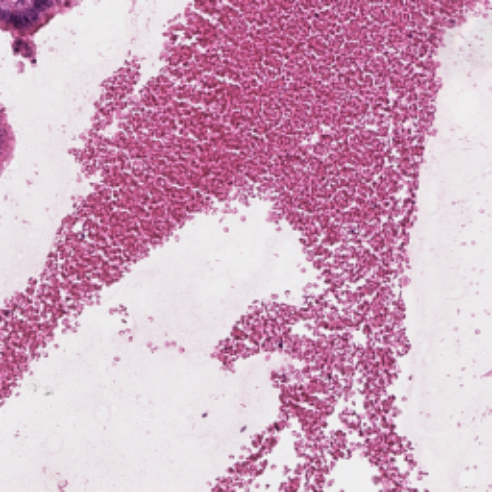

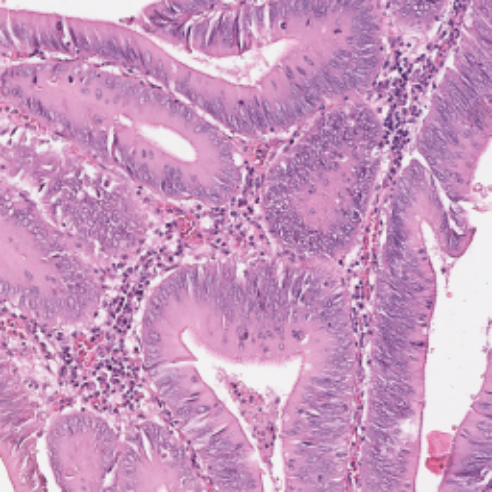

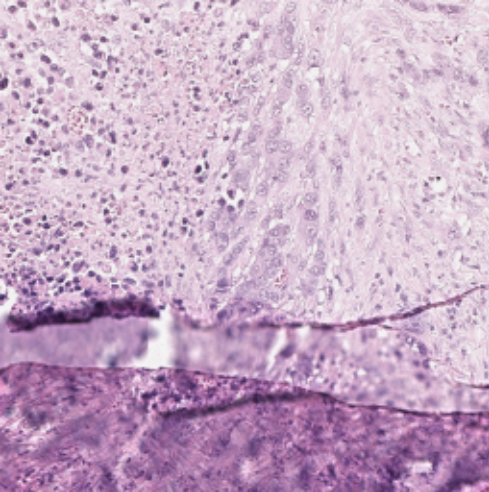

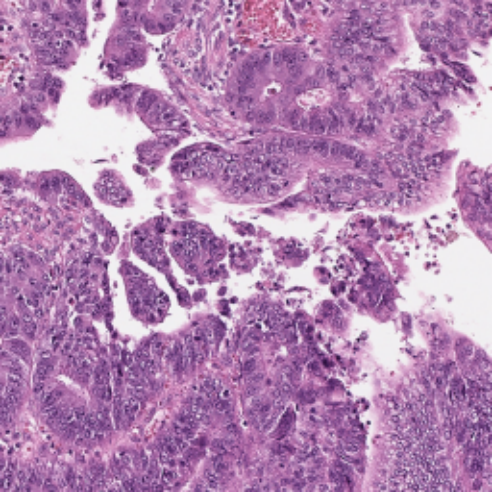

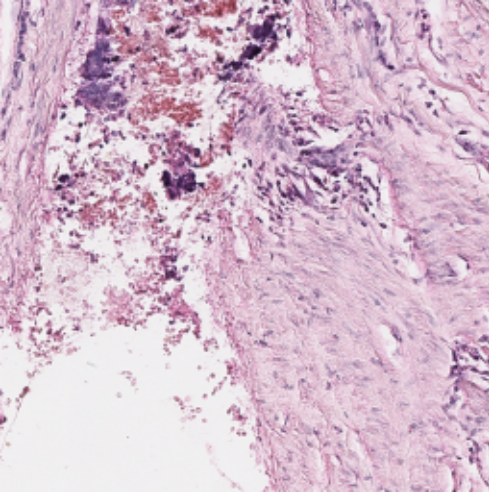

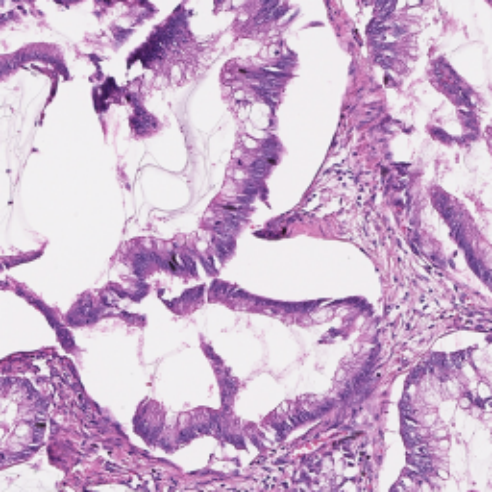

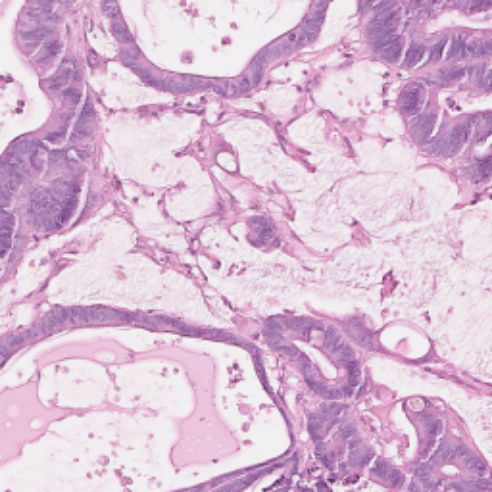

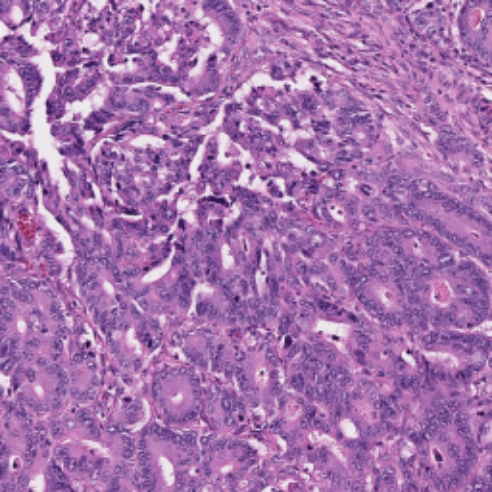

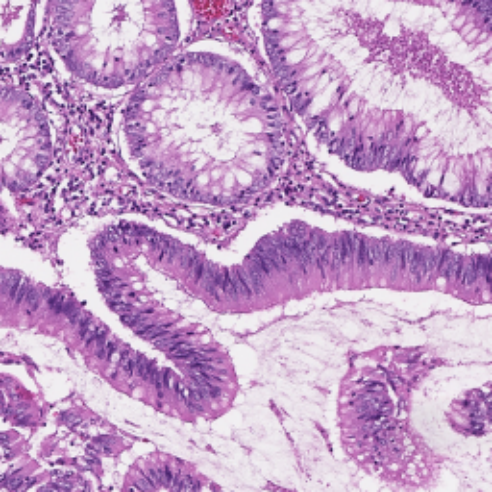

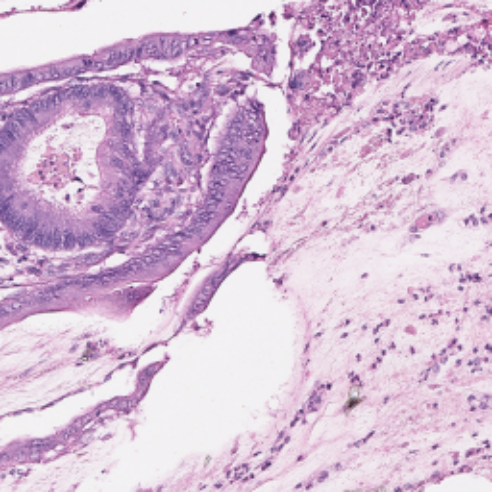

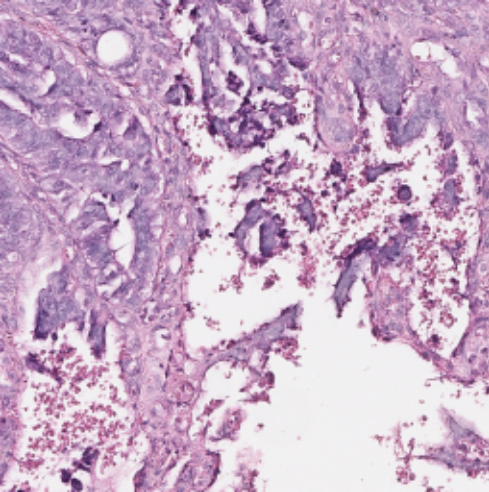

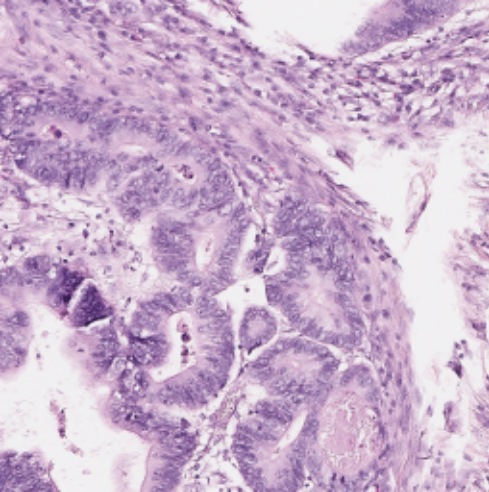

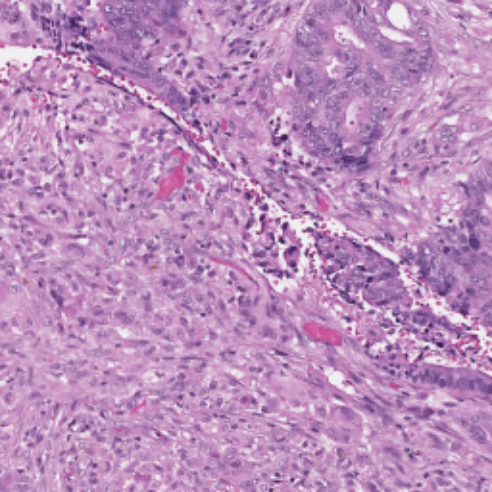

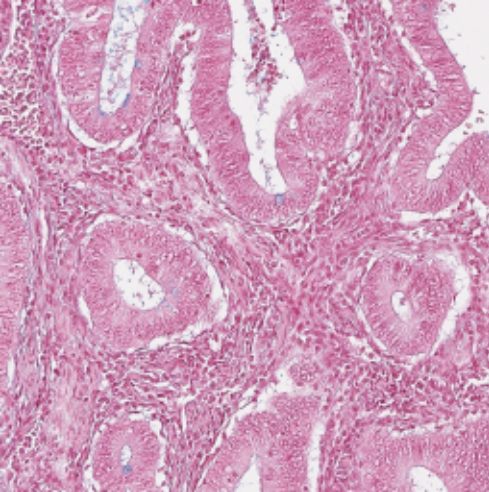

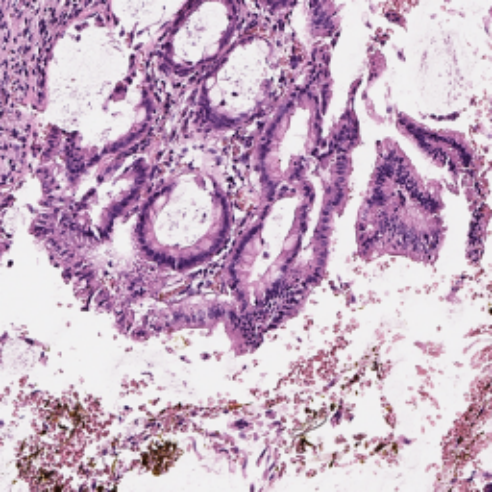

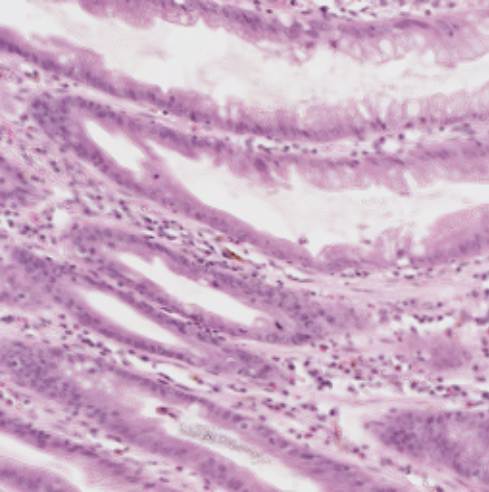

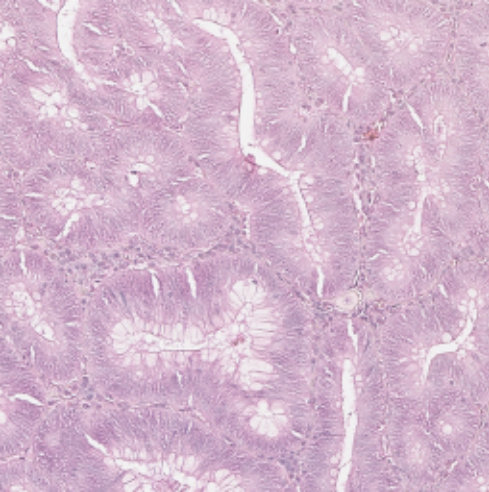

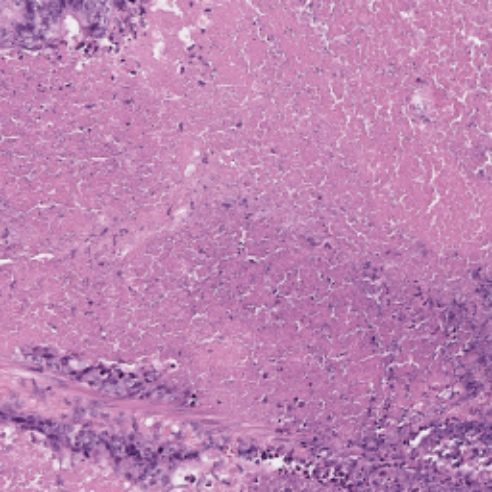

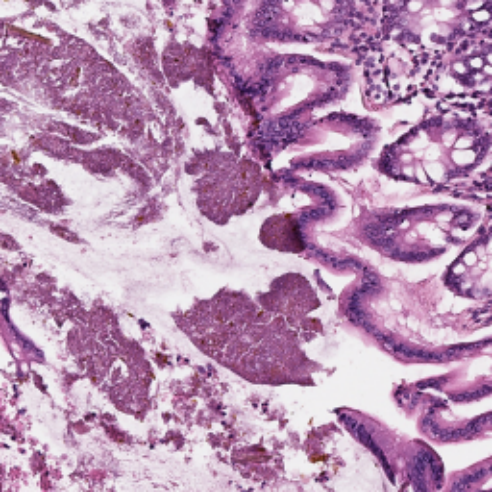

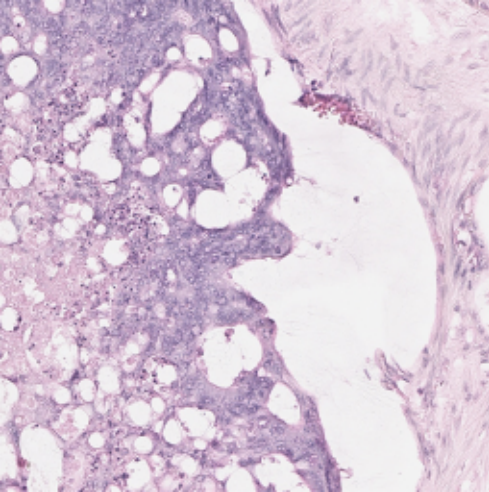

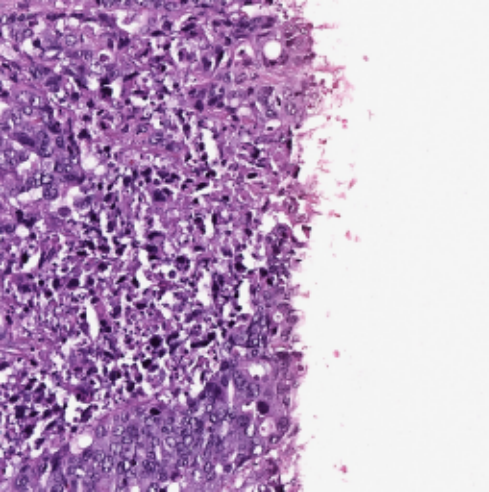

Supplement: Supplementary file 3 — Supplementary Data 3 [file 41746_2021_427_MOESM3_ESM.zip › assessment_patches_non-TAF.pdf]
