# Supplementary material for: Interpretable survival prediction for colorectal cancer using deep learning
Source: NPJ Digit Med. 2021 Apr 19;4:71. doi: 10.1038/s41746-021-00427-2 (PMC8055695; doi:10.1038/s41746-021-00427-2)
Supplement: Supplementary file 4 — Supplementary Information [file 41746_2021_427_MOESM4_ESM.pdf]

Supplementary Material

Supplementary Data 1

Zip file containing 2 PNG files, one containing 25 tumor-adipose feature (TAF) patches closest to the centroids, and one containing 25 randomly-sampled TAF patches, used for human graders as learning material to identify TAF (see “Tumor-adipose Feature” section in Methods).

Supplementary Data 2

Zip file containing 2 PDF files, one containing 25 TAF patches, and one containing 25 non-TAF patches, used for human graders to practice identifying TAF (see “Tumor-adipose Feature” section in Methods). Patches were presented to human graders in a random order.

Supplementary Data 3

Zip file containing 2 PDF files, one containing 100 TAF patches, and one containing 100 non-TAF patches, used for human graders to evaluate their ability to identify TAF (see “Tumor-adipose Feature” section in Methods). Patches were presented to human graders in a random order.

Supplementary Figures

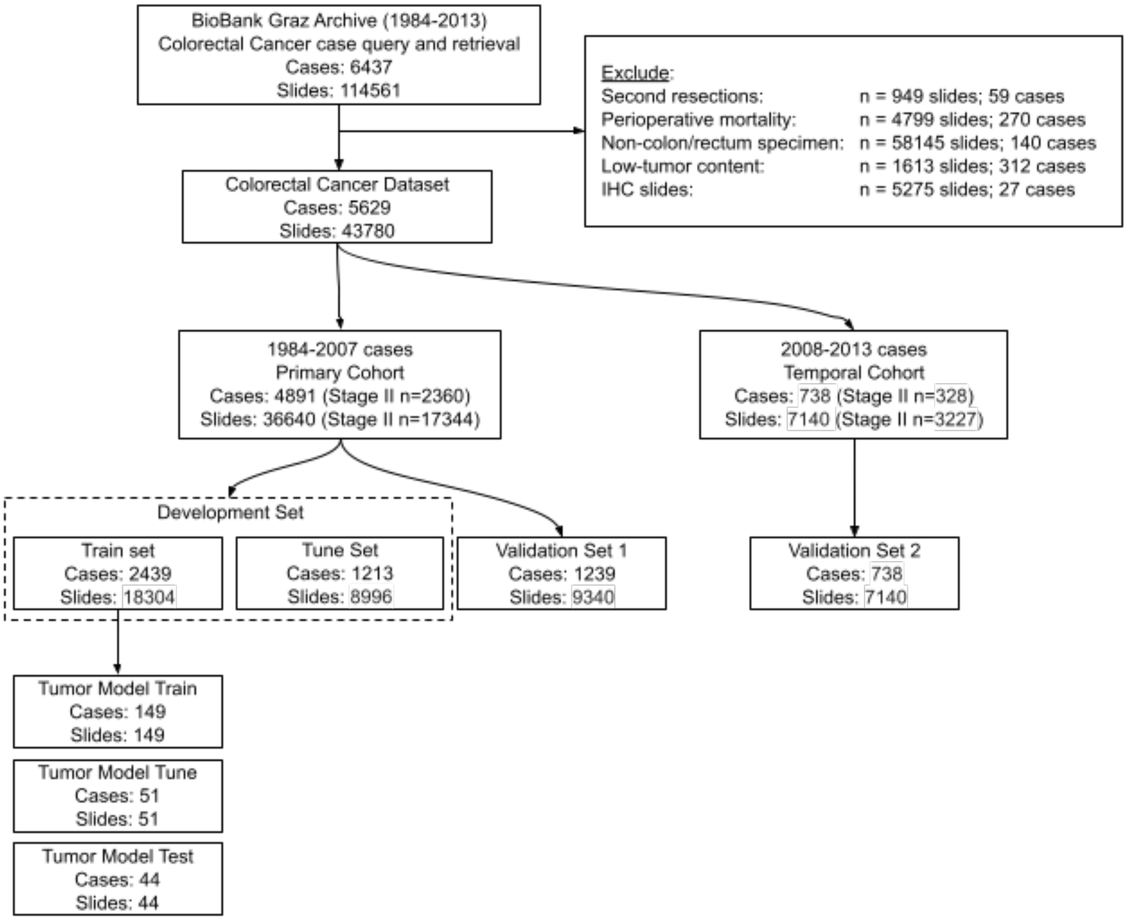

Supplementary Figure 1. STARD diagram of the dataset curation process.

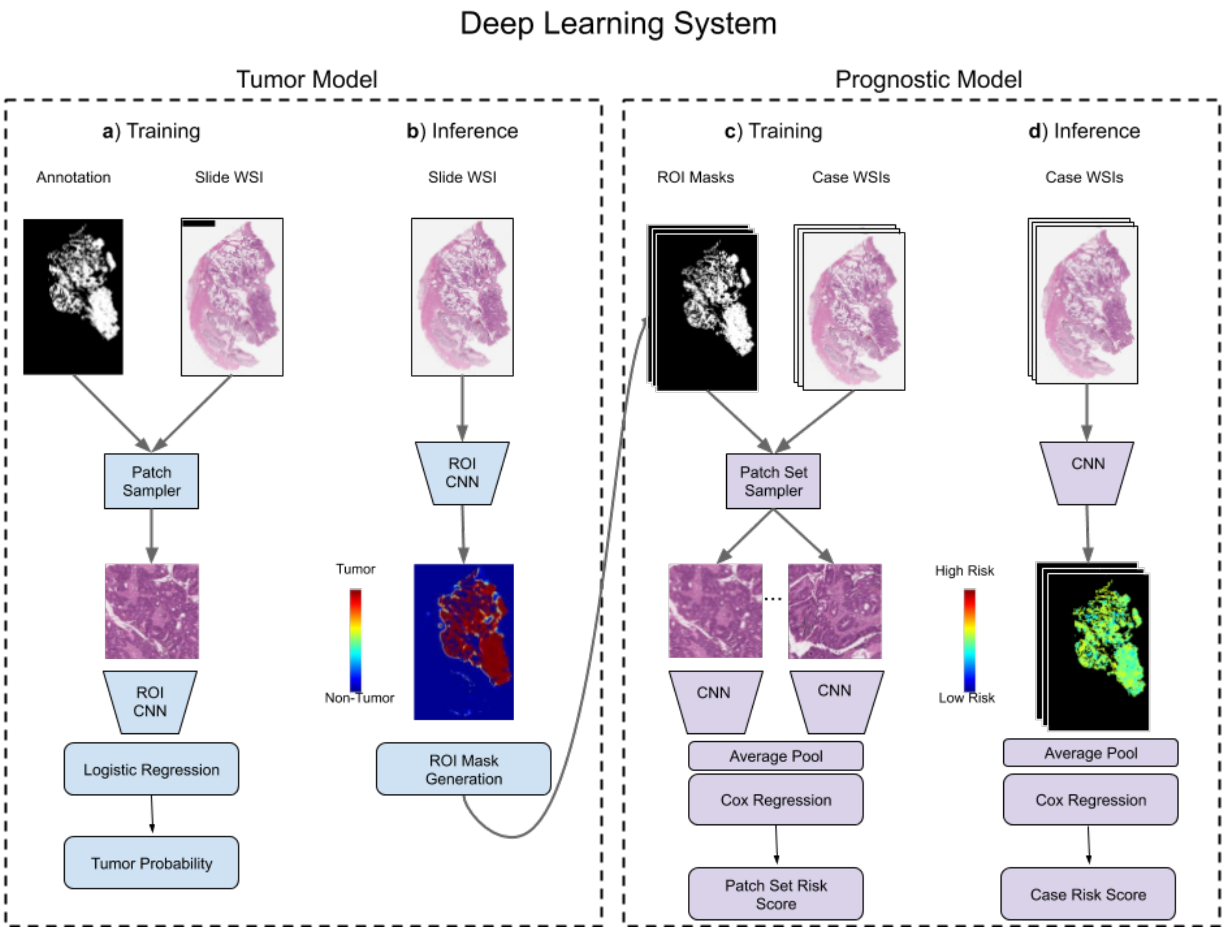

Supplementary Figure 2. Overview of deep learning system (DLS) development. (**a**) tumor model development: the tumor model was trained at the patch-level to identify colorectal adenocarcinoma from pixel-level pathologist annotations. (**b**) tumor model inference: the tumor model was run over all slides to produce region of interest (ROI) heatmaps that were binarized to generate ROI masks. (**c**) prognostic model development: The model was trained to predict case-level disease-specific survival. During training, a case is approximated by sampling a small number of patches from across the ROIs in a case. (**d**) prognostic model inference: at inference time, the prognostic model was run exhaustively across all ROIs to produce a case-level risk score. Scale bar indicates 5 mm. Note that the patch sampler's output image patches are shown for illustrative purposes only; the actual patch sizes will vary depending on the magnification (Supplementary Tables 8 and 10).

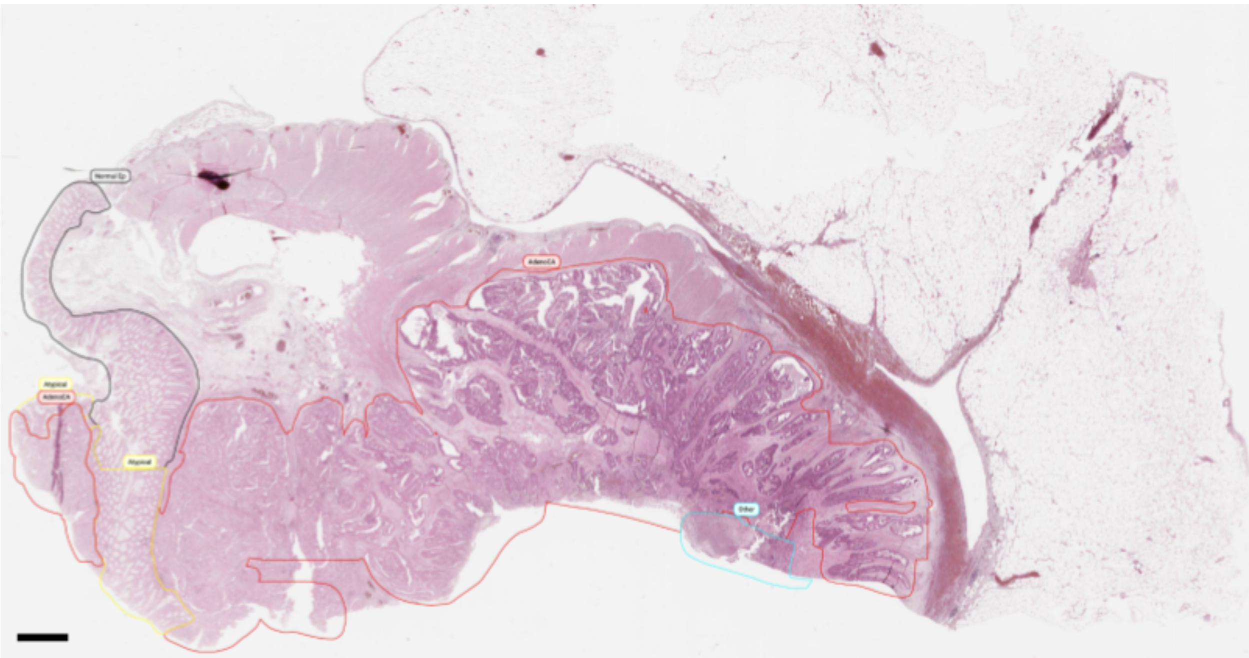

Supplementary Figure 3. Example of slide annotations for tumor model development. Annotations were provided for multiple types of histologies (e.g. normal epithelium, adenocarcinoma, atypical, and “other”). The model was developed to differentiate between colon adenocarcinoma and all other classes. Scale bar indicates 1 mm.

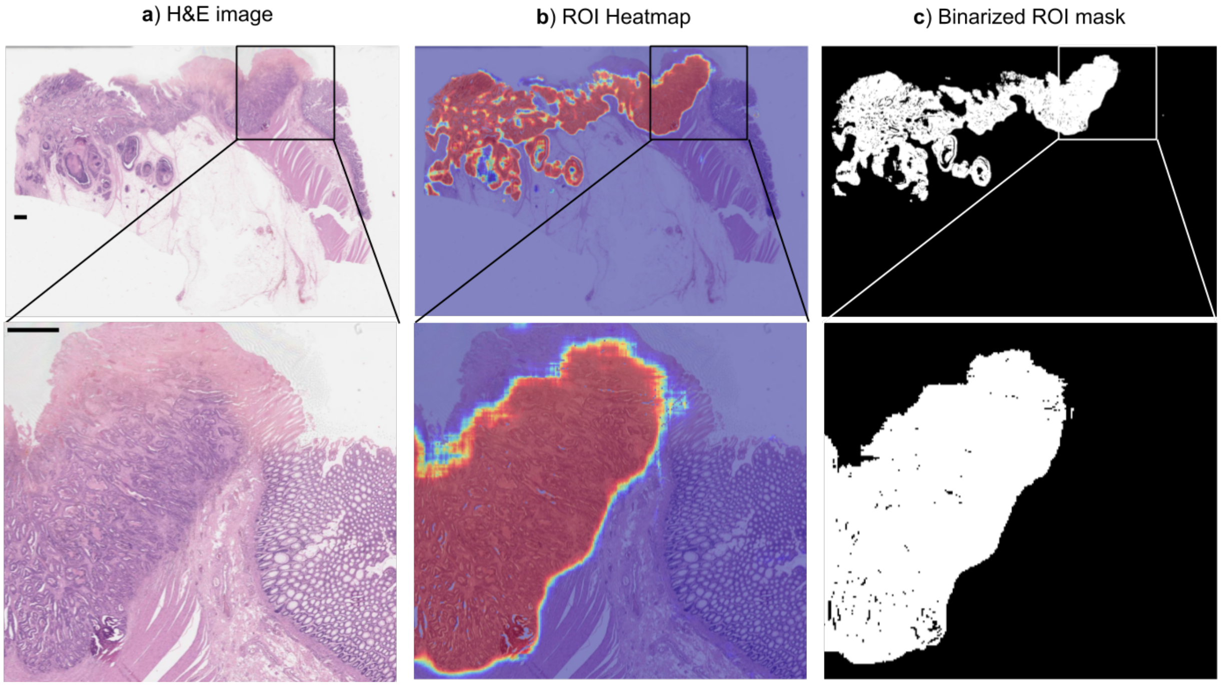

Supplementary Figure 4. Sample tumor segmentation model predictions and derived binary ROI mask that is used to sample image patches for the prognostic model. Scale bars indicate 1 mm.

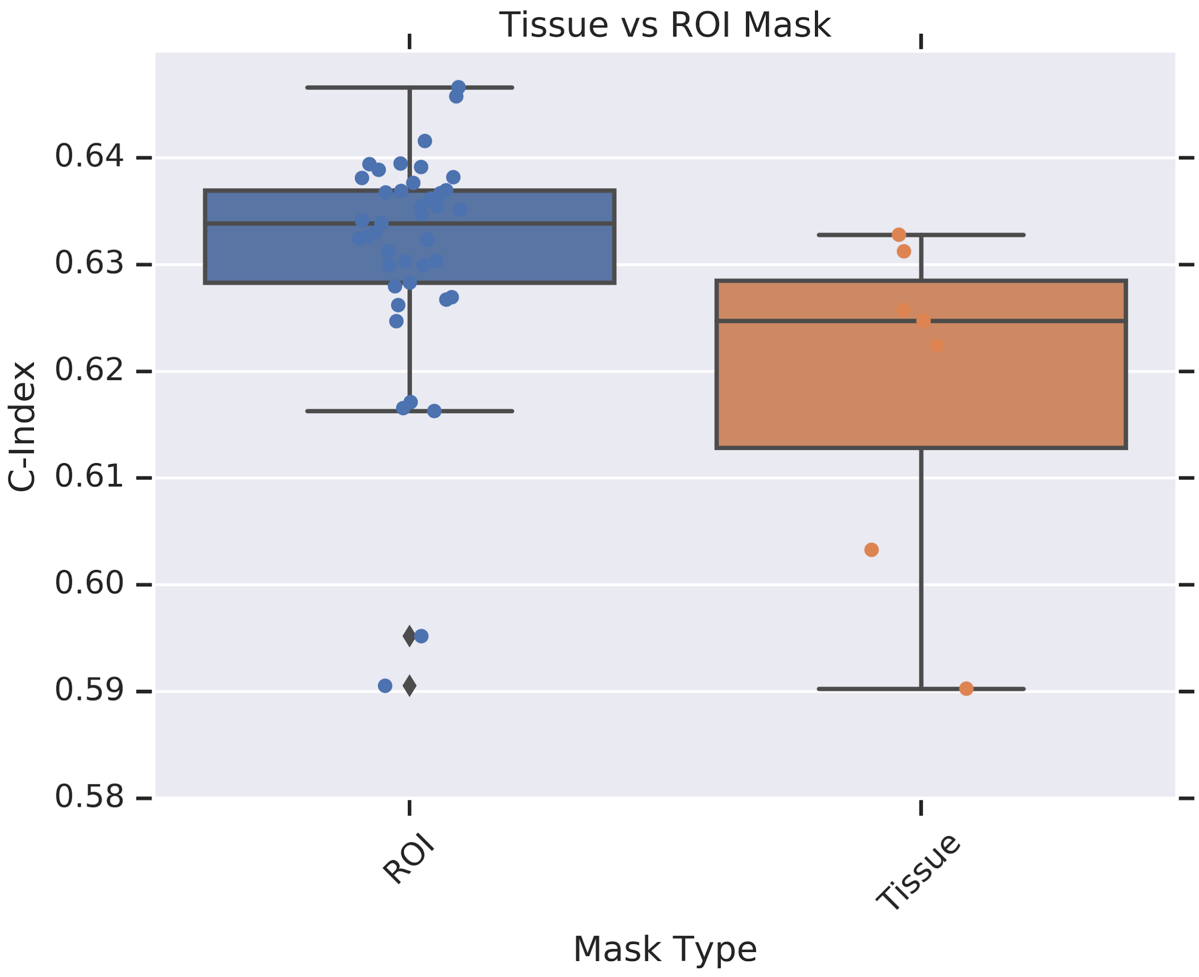

Supplementary Figure 5. Comparison of training using the entire tissue versus on a region of interest (ROI) derived using the tumor segmentation model. Variation in these box plots stems from different learning rates for both types of models and different mask generation parameters for the models trained on ROI masks. Models were evaluated on the tune set. Edges of boxes indicate quartiles, whiskers represent the ranges, and outliers are defined by 1.5 times the interquartile range.

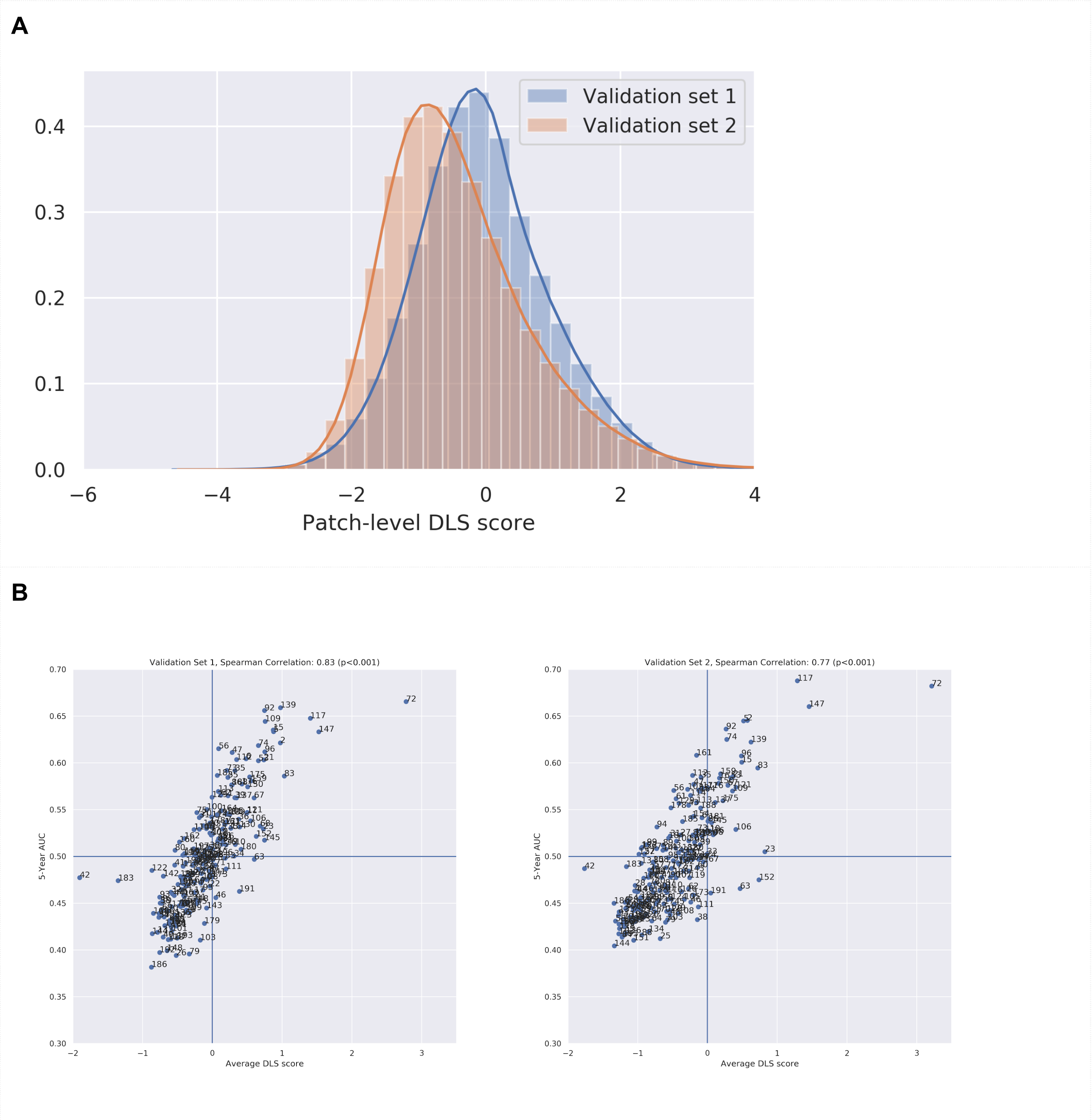

Supplementary Figure 6. Patch-level DLS score distribution for (A) all patches and (B) for each cluster. See also Supplementary Table 1 for comparison of clinicopathologic characteristics in validation set 1 and 2. Panel B additionally compares the average DLS score distribution with the 5-year AUC.

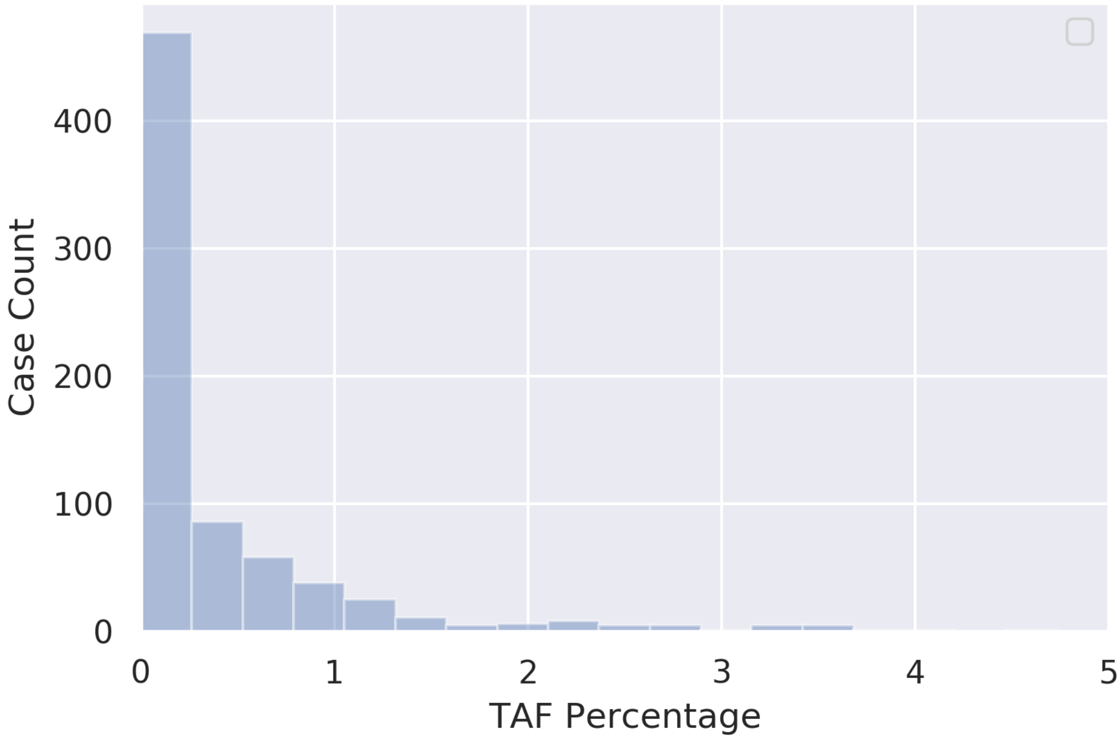

Supplementary Figure 7. Histogram of the percentage of the region of interest that is composed of the tumor-adipose feature (TAF) in validation set 2.

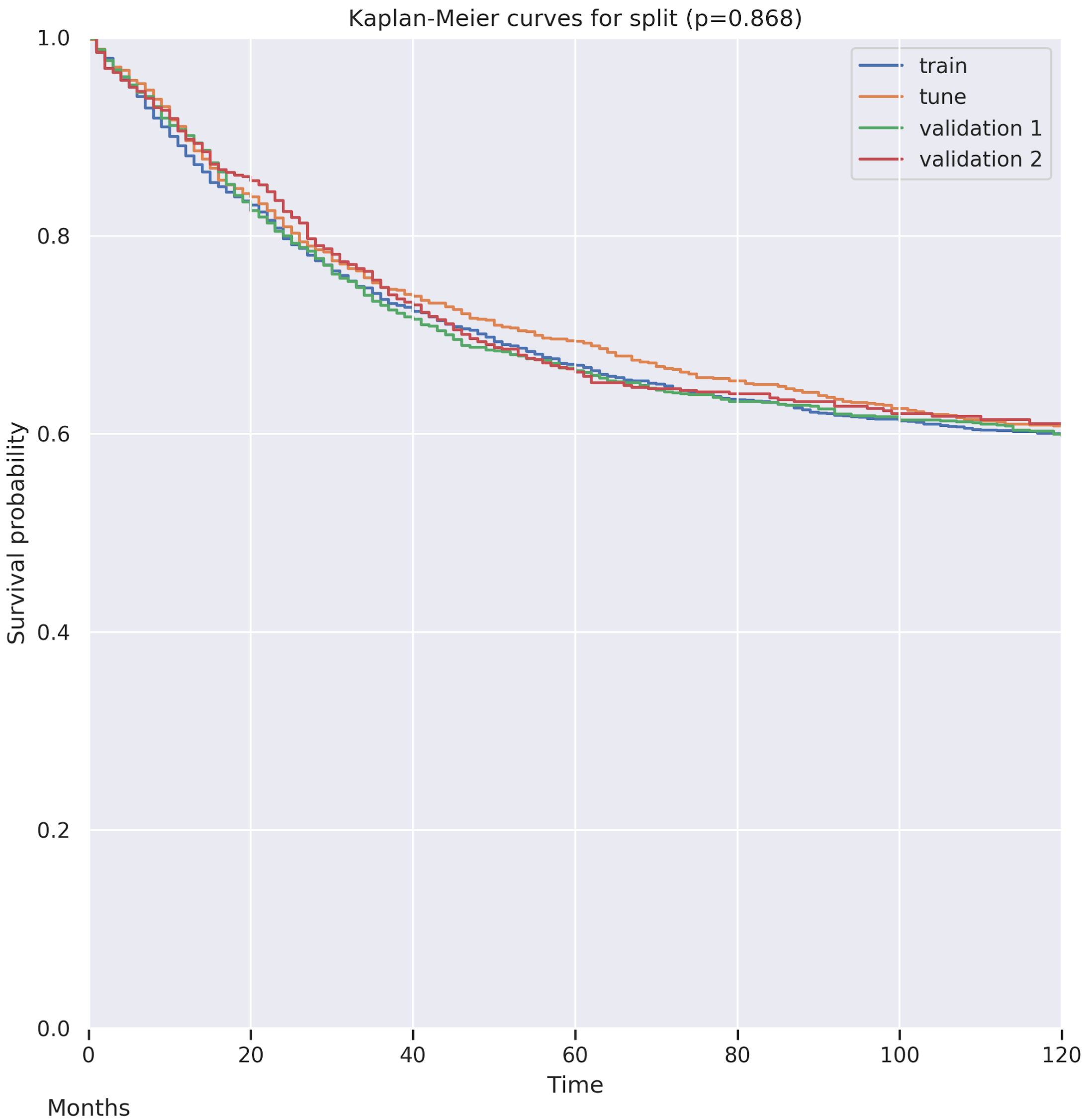

Months	0	20	40	60	80	100	120
train	2439	1948	1638	1440	1311	1205	1106
tune	1213	988	837	755	673	609	558
validation 1	1239	986	820	722	662	617	558
validation 2	738	607	498	433	348	238	124

Supplementary Figure 8. Kaplan Meier curves for all cases in the train, tune, and validation sets.

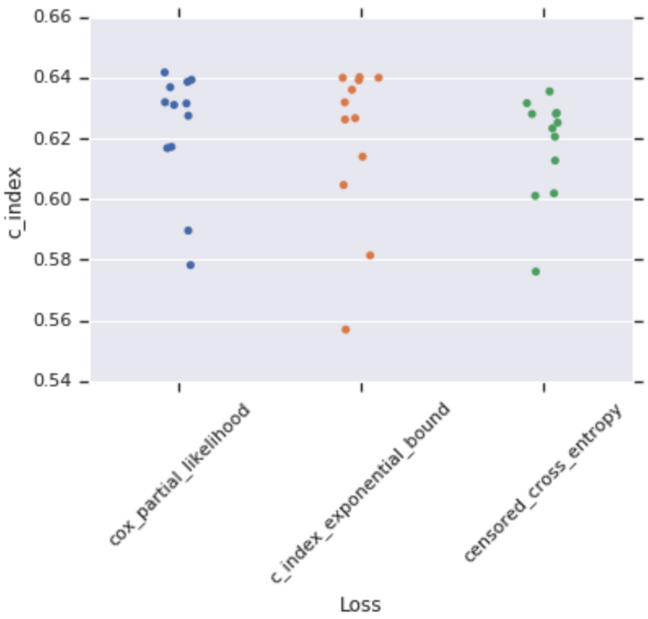

Supplementary Figure 9. Comparison of loss functions for DLS training. We compared three loss functions for DLS training: Cox partial likelihood, exponential lower bound on concordance index, and censored cross-entropy. For each loss function, 3 batch sizes (64, 128, 256) and 4 learning rates (10e-3, 5e-4, 10e-4, 5e-5, 10e-5) were tried. Models were evaluated on the tune set.

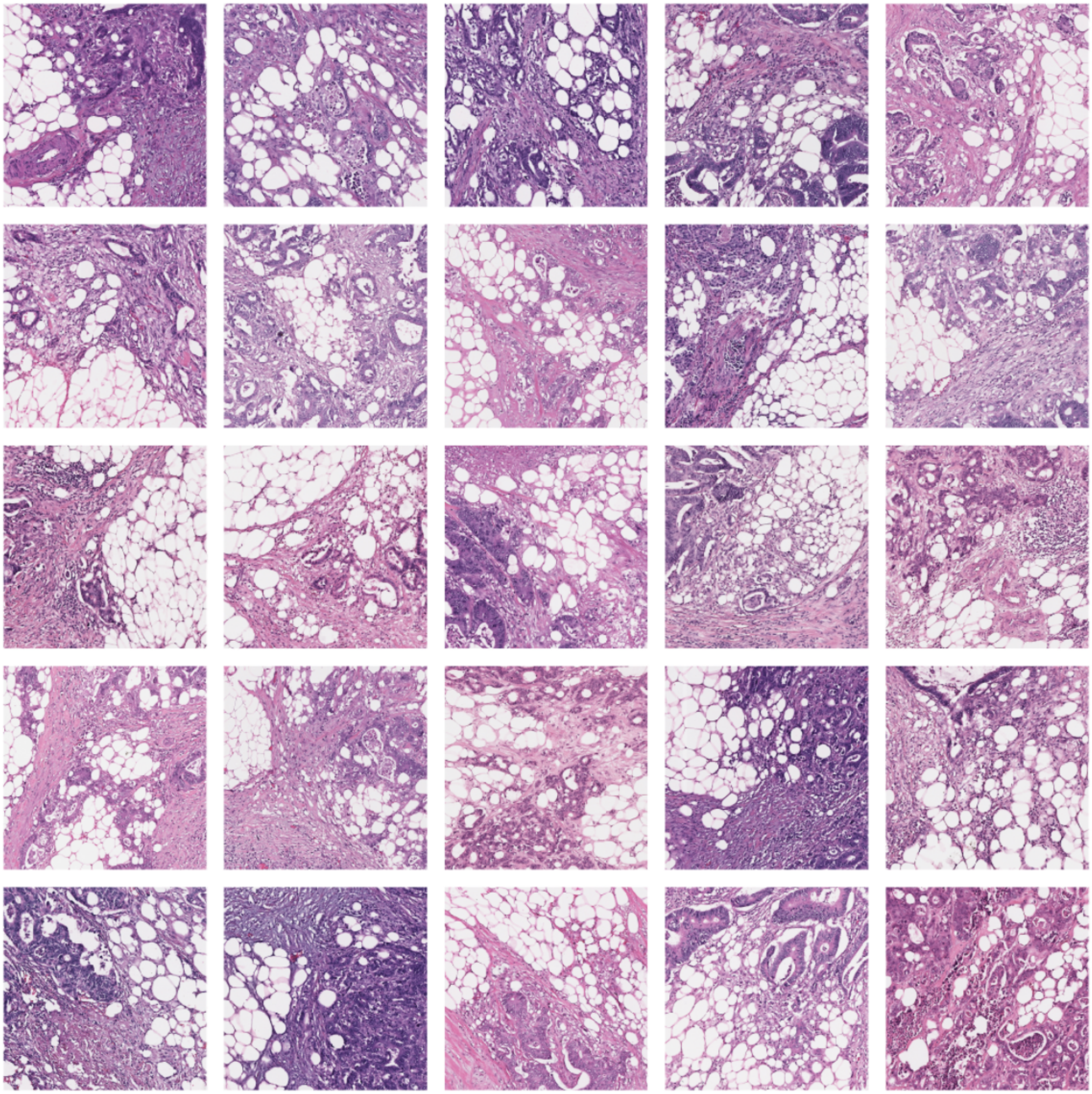

Supplementary Figure 10. Sample patches of the TAF cluster (each from a unique case), but with the clustering centroids fit on validation set 2 and used to extract patches from validation set 1.

Supplementary Tables

Supplementary Table 1. Clinical metadata distribution of the two validation sets. P-values for differences in proportions were calculated via individual t-tests.

		Stage II			Stage III		
		**Validation set 1**	**Validation set 2**	**P-value for difference**	**Validation set 1**	**Validation set 2**	**P-value for difference**
**T category**	**T1/T2**	0 (0%)	0 (0%)	N/A	70 (11%)	42 (10%)	0.7083
	**T3**	546 (91%)	270 (82%)	**0.0004**	439 (69%)	254 (62%)	**0.0235**
	**T4**	55 (9%)	58 (18%)	**0.0004**	129 (20%)	114 (28%)	**0.0055**
**N category**	**N0**	601 (100%)	328 (100%)	N/A	0 (0%)	0 (0%)	N/A
	**N1**	0 (0%)	0 (0%)	N/A	361 (57%)	245 (60%)	0.3095
	**N2**	0 (0%)	0 (0%)	N/A	189 (30%)	158 (39%)	**0.0032**
	**N3**	0 (0%)	0 (0%)	N/A	88 (14%)	7 (2%)	**0.0000**
**R category**	**R0**	588 (98%)	320 (98%)	0.7907	606 (95%)	392 (96%)	0.6388
	**R1**	13 (2%)	8 (2%)	0.7907	32 (5%)	18 (4%)	0.6388
**L category**	**L0**	532 (89%)	272 (83%)	**0.0231**	501 (79%)	274 (67%)	**0.0000**
	**L1**	69 (11%)	56 (17%)	**0.0231**	137 (21%)	136 (33%)	**0.0000**
**V category**	**V0**	580 (97%)	295 (90%)	**0.0004**	583 (91%)	312 (76%)	**0.0000**
	**V1**	21 (3%)	33 (10%)	**0.0004**	55 (9%)	98 (24%)	**0.0000**
**Tumor grade**	**G1**	27 (4%)	23 (7%)	0.1264	16 (3%)	17 (4%)	0.1598
	**G2**	464 (77%)	219 (67%)	**0.0009**	428 (67%)	226 (55%)	**0.0001**
	**G3**	102 (17%)	80 (24%)	**0.0089**	188 (29%)	155 (38%)	**0.0056**
	**GX**	8 (1%)	6 (2%)	0.5700	6 (1%)	12 (3%)	**0.0307**
**Self-reported sex**	**Male**	340 (57%)	202 (62%)	0.1369	339 (53%)	204 (50%)	0.2861
	**Female**	261 (43%)	126 (38%)	0.1369	299 (47%)	206 (50%)	0.2861
Age at diagnosis	**<= 59**	117 (19%)	43 (13%)	**0.0102**	149 (23%)	90 (22%)	0.5960
	**60-69**	166 (28%)	83 (25%)	0.4433	193 (30%)	99 (24%)	**0.0290**
	**70-79**	223 (37%)	116 (35%)	0.5982	210 (33%)	120 (29%)	0.2120
	**>= 80**	95 (16%)	86 (26%)	**0.0003**	86 (13%)	101 (25%)	**0.0000**

Supplementary Table 2. KM estimate of 5-year disease-specific survival in risk groups stratified by the deep learning system (DLS). Numbers in square brackets represent 95% confidence intervals.

Dataset	Risk Group	Stage II	Stage III	Stage II/II
Validation set 1	High<br>(top quartile)	72.84 [67.59, 77.38]	41.45 [35.66, 47.13]	53.33 [49.16, 57.32]
	Intermediate<br>(middle quartiles)	88.40 [82.75, 92.28]	62.12 [55.98, 67.66]	76.91 [72.81, 80.48]
	Low<br>(bottom quartile)	89.03 [79.25, 94.36]	76.32 [62.06, 85.81]	86.12 [78.87, 91.03]
Validation set 2	High<br>(top quartile)	57.07 [44.05, 68.13]	42.72 [32.25, 52.76]	46.10 [38.28, 53.56]
	Intermediate<br>(middle quartiles)	77.76 [68.87, 84.40]	52.82 [44.80, 60.21]	64.83 [58.78, 70.22]
	Low<br>(bottom quartile)	85.56 [78.29, 90.54]	73.07 [64.79, 79.70]	80.01 [74.66, 84.35]

Supplementary Table 3. Univariable Cox regression on the validation sets. Numbers indicate hazard ratio followed by 95% confidence intervals in square brackets, and p-values (from a Wald test) after the comma. *N/A because stage II only contains N0 and T3 or T4 and stage II only contains N1 by definition (American Joint Committee on Cancer, AJCC). Bold indicates statistically significant input variables ($p < 0.05$).

Variable		Stage II		Stage III		Stage II/III	
		Validation set 1	Validation set 2	Validation set 1	Validation set 2	Validation set 1	Validation set 2
DLS		**1.64 [1.40, 1.92], <0.001**	**1.55 [1.25, 1.92], <0.001**	**1.49 [1.33, 1.67], <0.001**	**1.51 [1.32, 1.74], <0.001**	**1.72 [1.57, 1.89], <0.001**	**1.64 [1.47, 1.84], <0.001**
Age		1.06 [0.91, 1.24], 0.446	**1.49 [1.18, 1.87], <0.001**	**1.11 [1.01, 1.22], 0.025**	**1.24 [1.09, 1.41], 0.001**	1.06 [0.98, 1.15], 0.121	**1.25 [1.12, 1.40], <0.001**
Sex	Male	1.0 (reference)					
	Female	0.78 [0.56, 1.07], 0.127	0.90 [0.57, 1.42], 0.653	0.79 [0.63, 0.98], 0.036	0.94 [0.70, 1.26], 0.682	**0.80 [0.67, 0.97], 0.019**	1.01 [0.79, 1.29], 0.929
Grade	G1	1.0 (reference)					
	G2	0.78 [0.38, 1.60], 0.503	1.67 [0.61, 4.62], 0.320	1.27 [0.56, 2.86], 0.563	3.06 [0.97, 9.65], 0.056	1.09 [0.64, 1.86], 0.754	**2.36 [1.11, 5.03], 0.027**
	G3	1.17 [0.54, 2.54], 0.682	1.49 [0.51, 4.42], 0.467	1.89 [0.83, 4.31], 0.128	**3.74 [1.18, 11.87], 0.025**	**1.81 [1.05, 3.14], 0.034**	**2.94 [1.36, 6.33], 0.006**
	GX	0.90 [0.19, 4.22], 0.889	2.38 [0.44, 13.00], 0.317	0.90 [0.18, 4.47], 0.899	2.92 [0.70, 12.21], 0.143	0.93 [0.31, 2.83], 0.902	2.75 [0.96, 7.84], 0.059
Lymphatic Invasion	L0	1.0 (reference)					
	L1	**1.71 [1.12, 2.61], 0.012**	1.02 [0.57, 1.81], 0.956	0.81 [0.61, 1.07], 0.138	1.23 [0.91, 1.66], 0.186	1.17 [0.92, 1.47], 0.199	**1.35 [1.04, 1.75], 0.026**
N-category	N0	N/A*				1.0 (reference)	
	N1	N/A*		1.0 (reference)		**2.16 [1.73, 2.69], 0.001**	**1.73 [1.28, 2.33], 0.001**
	N2	N/A*		**1.29 [1.00, 1.65], 0.046**	**1.78 [1.33, 2.38], 0.001**	**2.78 [2.16, 3.57], 0.001**	**3.09 [2.28, 4.19], 0.001**
	N3	N/A*		1.29 [0.93, 1.79], 0.129	0.70 [0.17, 2.83], 0.615	**2.79 [2.00, 3.89], 0.001**	**1.21 [0.30, 4.91], 0.793**
Margin Status	R0	1.0 (reference)					
	R1	1.19 [0.44, 3.21], 0.732	1.84 [0.58, 5.83], 0.301	1.44 [0.89, 2.31], 0.136	1.04 [0.51, 2.11], 0.921	**1.56 [1.01, 2.39], 0.043**	1.32 [0.72, 2.42], 0.365
Margin Status	T1/T2	N/A*		1.0 (reference)			
	T3	1.0 (reference)		**1.67 [1.09, 2.55], 0.017**	**2.81 [1.31, 6.06], 0.008**	1.06 [0.70, 1.61], 0.770	2.02 [0.95, 4.32], 0.068
	T4	**1.68 [1.06, 2.66], 0.027**	**1.93 [1.16, 3.20], 0.011**	**2.37 [1.50, 3.75], 0.001**	**6.42 [2.96, 13.94], 0.001**	**1.90 [1.22, 2.97], 0.005**	**4.95 [2.30, 10.66], 0.001**
Venous Invasion	V0	1.0 (reference)					
	V1	1.76 [0.90, 3.46], 0.099	1.43 [0.74, 2.77], 0.292	0.92 [0.61, 1.38], 0.671	**1.63 [1.19, 2.25], 0.003**	1.26 [0.89, 1.78], 0.199	**1.83 [1.38, 2.43], 0.001**

Supplementary Table 4. (A) 5-year AUC for the deep learning system (DLS) and Cox regression models fit on the clinical metadata, and Cox models fit on both; (B) a similar table for the tumor-adipose feature (TAF) quantitation. Numbers in square brackets represent 95% confidence intervals.

A

Dataset	Stage	DLS	Clinical	Clinical + DLS	Delta
Validation set 1	Stage II	0.680 [0.631, 0.739]	0.539 [0.485, 0.610]	0.659 [0.612, 0.716]	0.120 [0.076, 0.188]
	Stage III	0.655 [0.617, 0.694]	0.597 [0.550, 0.645]	0.662 [0.631, 0.709]	0.065 [0.026, 0.108]
	Stage II/III	0.698 [0.660, 0.729]	0.678 [0.642, 0.705]	0.733 [0.697, 0.759]	0.055 [0.036, 0.074]
Validation set 2	Stage II	0.663 [0.592, 0.730]	0.610 [0.544, 0.657]	0.695 [0.629, 0.746]	0.085 [0.036, 0.150]
	Stage III	0.655 [0.600, 0.707]	0.664 [0.606, 0.720]	0.686 [0.624, 0.736]	0.022 [-0.022, 0.070]
	Stage II/III	0.686 [0.638, 0.723]	0.684 [0.639, 0.716]	0.721 [0.688, 0.753]	0.038 [0.006, 0.064]

B

Dataset	Stage	TAF	Clinical	Clinical + TAF	Delta
Validation set 1	Stage II	0.645 [0.598, 0.700]	0.539 [0.485, 0.610]	0.595 [0.543, 0.663]	0.056 [0.034, 0.082]
	Stage III	0.629 [0.593, 0.680]	0.597 [0.550, 0.645]	0.625 [0.587, 0.676]	0.029 [0.012, 0.047]
	Stage II/III	0.666 [0.634, 0.697]	0.678 [0.642, 0.705]	0.698 [0.664, 0.723]	0.020 [0.010, 0.029]
Validation set 2	Stage II	0.634 [0.570, 0.697]	0.610 [0.544, 0.657]	0.620 [0.555, 0.661]	0.010 [-0.016, 0.036]
	Stage III	0.682 [0.638, 0.743]	0.664 [0.606, 0.720]	0.689 [0.630, 0.743]	0.025 [0.004, 0.045]
	Stage II/III	0.682 [0.641, 0.734]	0.684 [0.639, 0.716]	0.699 [0.653, 0.734]	0.015 [0.006, 0.023]

Supplementary Table 5. C-index for the deep learning system (DLS) and Cox regression models fit on the clinical metadata, and Cox models fit on both. Numbers in square brackets represent 95% confidence intervals.

Dataset	Stage	DLS	Clinical	Clinical + DLS	Delta
Validation set 1	Stage II	0.651 [0.615, 0.703]	0.535 [0.493, 0.596]	0.634 [0.597, 0.680]	0.099 [0.070, 0.143]
	Stage III	0.626 [0.601, 0.655]	0.576 [0.542, 0.613]	0.626 [0.602, 0.654]	0.050 [0.030, 0.082]
	Stage II/III	0.663 [0.636, 0.686]	0.640 [0.608, 0.664]	0.685 [0.658, 0.704]	0.045 [0.031, 0.060]
Validation set 2	Stage II	0.628 [0.568, 0.687]	0.600 [0.554, 0.653]	0.658 [0.607, 0.704]	0.058 [0.015, 0.103]
	Stage III	0.639 [0.597, 0.678]	0.631 [0.591, 0.680]	0.653 [0.609, 0.690]	0.022 [-0.018, 0.060]
	Stage II/III	0.660 [0.624, 0.694]	0.661 [0.625, 0.688]	0.689 [0.659, 0.721]	0.028 [0.008, 0.050]

Supplementary Table 6. (A) 5-year AUC in T3 cases for the deep learning system (DLS) and Cox regression models fit on the clinical metadata, and Cox models fit on both. (B) a similar table for the tumor-adipose feature (TAF) quantitation. Numbers in square brackets represent 95% confidence intervals.

A

Dataset	Stage	DLS	Clinical	Clinical + DLS	Delta
Validation set 1 (T3 only)	Stage II	0.677 [0.616, 0.739]	0.537 [0.470, 0.598]	0.657 [0.604, 0.714]	0.121 [0.064, 0.179]
	Stage III	0.639 [0.581, 0.684]	0.563 [0.515, 0.620]	0.654 [0.599, 0.708]	0.091 [0.025, 0.129]
	Stage II/III	0.697 [0.661, 0.739]	0.668 [0.629, 0.694]	0.733 [0.698, 0.770]	0.065 [0.047, 0.087]
Validation set 2 (T3 only)	Stage II	0.642 [0.567, 0.729]	0.585 [0.502, 0.680]	0.679 [0.596, 0.766]	0.094 [0.037, 0.175]
	Stage III	0.629 [0.559, 0.690]	0.590 [0.515, 0.662]	0.641 [0.561, 0.702]	0.051 [-0.002, 0.116]
	Stage II/III	0.654 [0.598, 0.701]	0.641 [0.578, 0.702]	0.685 [0.632, 0.732]	0.044 [0.004, 0.080]

B

Dataset	Stage	TAF	Clinical	Clinical + TAF	Delta
Validation set 1 (T3 only)	Stage II	0.645 [0.590, 0.691]	0.537 [0.470, 0.598]	0.592 [0.526, 0.651]	0.055 [0.032, 0.092]
	Stage III	0.618 [0.558, 0.675]	0.563 [0.515, 0.620]	0.602 [0.555, 0.656]	0.038 [0.009, 0.059]
	Stage II/III	0.668 [0.634, 0.703]	0.668 [0.629, 0.694]	0.692 [0.659, 0.720]	0.025 [0.017, 0.035]
Validation set 2 (T3 only)	Stage II	0.604 [0.530, 0.712]	0.585 [0.502, 0.680]	0.600 [0.512, 0.692]	0.015 [-0.015, 0.056]
	Stage III	0.653 [0.576, 0.714]	0.590 [0.515, 0.662]	0.633 [0.564, 0.709]	0.043 [0.022, 0.070]
	Stage II/III	0.649 [0.599, 0.707]	0.641 [0.578, 0.702]	0.666 [0.612, 0.721]	0.025 [0.011, 0.039]

Supplementary Table 7. Spearman correlation between clinicopathologic features and (A) the deep learning system (DLS) or (B) automatic quantitation of the tumor-adipose feature. P-values (from a t-test) are shown in parentheses. Cells with a p-value below 0.05 are bolded. Abbreviations for L/N/R/T/V/G are defined in the “Data Cohorts” section of Methods.

A

Dataset	Stage	T	N	R	L	V	G	Sex	Age
Validation set 1	Stage II	0.07 (0.080)	N/A	-0.08 (0.057)	0.07 (0.084)	0.02 (0.684)	**0.13 (0.002)**	0.0 (0.928)	**-0.09 (0.024)**
	Stage III	**0.27 (<0.001)**	**0.22 (<0.001)**	**0.14 (<0.001)**	-0.06 (0.141)	**0.11 (0.006)**	**0.23 (<0.001)**	0.03 (0.421)	-0.07 (0.067)
	Stage II/III	**0.18 (<0.001)**	**0.36 (<0.001)**	**0.07 (0.009)**	0.04 (0.179)	**0.10 (<0.001)**	**0.22 (<0.001)**	0.03 (0.322)	**-0.10 (0.001)**
Validation set 2	Stage II	**0.18 (0.001)**	N/A	0.11 (0.054)	0.09 (0.093)	**0.14 (0.010)**	**0.17 (0.003)**	0.07 (0.183)	0.04 (0.517)
	Stage III	**0.27 (<0.001)**	**0.19 (<0.001)**	**0.10 (0.038)**	**0.13 (0.008)**	**0.16 (0.001)**	**0.17 (0.001)**	-0.01 (0.791)	-0.04 (0.433)
	Stage II/III	**0.24 (<0.001)**	**0.34 (<0.001)**	**0.12 (0.001)**	**0.17 (<0.001)**	**0.21 (<0.001)**	**0.20 (<0.001)**	0.06 (0.115)	-0.04 (0.339)

B

Dataset	Stage	T	N	R	L	V	G	Sex	Age
Validation set 1	Stage II	**0.12 (0.003)**	N/A	0.01 (0.890)	0.04 (0.371)	-0.00 (0.986)	**0.15 (0.000)**	-0.02 (0.617)	-0.00 (0.974)
	Stage III	**0.36 (0.000)**	**0.13 (0.001)**	**0.12 (0.003)**	0.02 (0.573)	**0.16 (0.000)**	**0.16 (0.000)**	-0.03 (0.413)	0.05 (0.230)
	Stage II/III	**0.27 (0.000)**	**0.28 (0.000)**	**0.09 (0.002)**	**0.06 (0.024)**	**0.12 (0.000)**	**0.18 (0.000)**	-0.02 (0.513)	0.01 (0.785)
Validation set 2	Stage II	**0.17 (0.002)**	N/A	**0.16 (0.005)**	0.03 (0.611)	**0.12 (0.025)**	0.05 (0.384)	-0.14 (0.012)	0.05 (0.357)
	Stage III	**0.46 (0.000)**	**0.17 (0.000)**	0.08 (0.093)	**0.14 (0.005)**	**0.20 (0.000)**	0.03 (0.591)	-0.07 (0.161)	0.05 (0.278)
	Stage II/III	**0.37 (0.000)**	**0.23 (0.000)**	**0.12 (0.001)**	**0.13 (0.000)**	**0.20 (0.000)**	**0.07 (0.072)**	-0.07 (0.053)	0.04 (0.282)

Supplementary Table 8. Hyperparameter search space and optimal hyperparameters for the tumor segmentation model. We used random search (n=50 configurations) and selected the best model checkpoint based on the tuning set 5-year AUC.

Hyperparameter	Description	Values	Optimal configuration
Batch size	Number of examples in each training batch	64	64
Patch size	Height and width of each image patch	299	299
Magnification	Image magnification at which the patches are extracted	20X, 10X, 5X, 2.5X, 1.25X	5X
Neural network architecture	Convolutional neural network architecture	InceptionV3	InceptionV3
Depth Multiplier	Multiplier on the depth of each convolution layer for downscaling the number of parameters in the default network architecture	0.05, 0.1, 0.15, 0.2	0.1
Loss	Loss function used for training	Softmax cross-entropy	Softmax cross-entropy
Optimizer	The optimization algorithm used for model training	RMSProp	RMSProp
L2 regularization weight	Weight of the L2 loss used for regularization	0.001, 0.0001, 0.00001	0.0001
Initial learning rate	Initial learning rate used for the RMSPROP optimizer; decay rate was 0.99 every 20,000 steps	0.005, 0.0005, 0.00005	0.005
Learning rate decay steps	Number of steps after which the learning rate is decreased by multiplying by the decay rate	10000, 20000	10000
Learning rate decay rate	The rate at which the learning rate is decayed after a fixed number of steps	0.95, 0.99	0.99
Exponential moving average decay rate	Decay rate used for taking an exponential moving average of the model weights for evaluation	None, 0.999, 0.9999	0.999
Training steps	The number of steps for which the model is trained	2000000	2000000
Evaluation steps	The number of train steps after which the model is evaluated	10000	10000

Supplementary Table 9. Tumor segmentation model performance on its test split at three different thresholds. Thresholds were chosen based on the recall observed on the tune split. AUC was 98.50.

Threshold	Recall	Precision	Intersection over union
95% tune set recall	97.58	83.38	93.63
90% tune set recall	93.99	88.58	94.72
75% tune set recall	81.42	93.81	93.02

Supplementary Table 10. Hyperparameter search space and optimal hyperparameters for the prognostic model. (**A**) We used random search (n=100 configurations across the search space and selected the best model checkpoint based on the tuning set 5-year AUC. (**B**) The final DLS predictions were generated by ensembling the top 5 models.

A

Hyperparameter	Description	Value
Batch size*	Number of examples in each training batch.	64
Patch size*	Height and width of each image patch.	256
Patch set size*	Number of patches sampled from a case to form a single training example:	16
Magnification	Image magnification at which the patches are extracted	20X, 10X, 5X, 2.5X
ROI model recall	The recall for tumor detection. Recall of 100 corresponds to using a tissue mask instead of an ROI mask.	100, 95, 90, 75
ROI region dilation	The number of superpixels by which the ROI mask is dilated	0, 4, 16
Number of layers	Number of layers used in our MobileNet-based architecture	4, 8
Base depth	Depth of the first convolution layer in the network	8, 16, 32
Depth growth rate	The rate at which depth grows after each stride 2 layer.	1.25, 1.5, 2.0
Max depth	The maximum depth of any layer in the network	64, 256
Loss	Survival loss function used for training.	Cox partial likelihood
Optimizer	The optimization algorithm used for model training.	Adam
L2 regularization weight	Weight of the L2 loss used for regularization	0.001, 0.0001, 0.00001
Initial Learning rate	Initial learning rate used for the RMSPROP optimizer; decay rate was 0.99 every 20,000 steps.	0.005, 0.0005, 0.00005
Learning rate decay steps	Number of steps after which the learning rate is decreased by multiplying by the decay rate.	10000, 20000
Learning rate decay rate	The rate at which the learning rate is decayed after a fixed number of steps.	0.95, 0.99
Exponential moving average decay rate	Decay rate used for taking an exponential moving average of the model weights for evaluation.	None, 0.999, 0.9999
Training steps	The number of steps for which the model is trained.	2000000
Evaluation steps	The number of train steps after which the model is evaluated.	10000

* These parameters were chosen based on preliminary tuning experiments. The best values from these experiments were chosen for the full hyper-parameter tuning run described here.

B

**Hyperparameter**	**Model 1**	**Model 2**	**Model 3**	**Model 4**	**Model 5**
Magnification	5X	5X	5X	5X	5X
ROI model recall	90	90	90	90	95
ROI region dilation	16	4	4	4	16
Number of layers	8	4	4	8	8
Base depth	32	32	32	8	32
Depth growth rate	1.5	1.25	1.5	1.25	1.25
Max depth	256	64	64	256	256
L2 Regularization	1e-05	0.001	1e-05	0.001	0.001
Initial learning rate	0.0005	0.0005	0.0005	0.0005	5e-05
Learning rate decay steps	10000	10000	10000	20000	20000
Learning rate decay rate	0.95	0.95	0.95	0.95	0.95
Exponential moving average decay rate	0.9999	0.9999	0.9999	N/A	0.999
Training step	1381426	1403469	1907329	1714445	1259927

Supplementary Table 11. REMARK checklist for reporting.

Item to be reported		Location
**INTRODUCTION**		
1	State the marker examined, the study objectives, and any pre-specified hypotheses.	Last paragraph of introduction
**MATERIALS AND METHODS**		
Patients		
2	Describe the characteristics (e.g., disease stage or co-morbidities) of the study patients, including their source and inclusion and exclusion criteria.	"Data Cohorts" section
3	Describe treatments received and how chosen (e.g., randomized or rule-based).	"Data Cohorts" section
Specimen characteristics		
4	Describe type of biological material used (including control samples) and methods of preservation and storage.	"Data Cohorts" section
Assay methods		
5	Specify the assay method used and provide (or reference) a detailed protocol, including specific reagents or kits used, quality control procedures, reproducibility assessments, quantitation methods, and scoring and reporting protocols. Specify whether and how assays were performed blinded to the study endpoint.	"Data Cohorts" and "Prognostic Model Neural Network Architecture and Survival Loss" sections
Study design		
6	State the method of case selection, including whether prospective or retrospective and whether stratification or matching (e.g., by stage of disease or age) was used. Specify the time period from which cases were taken, the end of the follow-up period, and the median follow-up time.	"Data Cohorts" section
7	Precisely define all clinical endpoints examined.	"Data Cohorts" section
8	List all candidate variables initially examined or considered for inclusion in models.	"Data Cohorts" and "DLS Association with Clinicopathologic Features" section, Table 4
9	Give rationale for sample size; if the study was designed to detect a specified effect size, give the target power and effect size.	"Data Cohorts" section
Statistical analysis methods		
10	Specify all statistical methods, including details of any variable selection procedures and other model-building issues, how model assumptions were verified, and how missing data were handled.	"Tumor Segmentation Model", "Prognostic Model Neural Network Architecture and Survival Loss", and "Understanding DLS Predictions" sections
11	Clarify how marker values were handled in the analyses; if relevant, describe methods used for cutpoint determination.	"Evaluating DLS Performance" section
**RESULTS**		
Data		
12	Describe the flow of patients through the study, including the number of patients included in each stage of the analysis (a diagram may be helpful) and reasons for dropout. Specifically, both overall and for each subgroup extensively examined report the numbers of patients and the number of events.	Table 1, Supplementary Figure 1
13	Report distributions of basic demographic characteristics (at least age and sex), standard (disease-specific) prognostic variables, and tumor marker, including numbers of missing values.	Supplementary Table 1
Analysis and presentation		
14	Show the relation of the marker to standard prognostic variables.	Supplementary Tables 4 and 7
15	Present univariable analyses showing the relation between the marker and outcome, with the estimated effect (e.g., hazard ratio and survival probability). Preferably provide similar analyses for all other variables being analyzed. For the effect of a tumor marker on a time-to-event outcome, a Kaplan-Meier plot is recommended.	P5<br>Supplementary Table 3
16	For key multivariable analyses, report estimated effects (e.g., hazard ratio) with confidence intervals for the marker and, at least for the final model, all other variables in the model.	Table 3

17	Among reported results, provide estimated effects with confidence intervals from an analysis in which the marker and standard prognostic variables are included, regardless of their statistical significance.	Table 3, Supplementary Tables 4 and 5
18	If done, report results of further investigations, such as checking assumptions, sensitivity analyses, and internal validation.	Tables 4,5
**DISCUSSION**		
19	Interpret the results in the context of the pre-specified hypotheses and other relevant studies; include a discussion of limitations of the study.	Throughout Discussion
20	Discuss implications for future research and clinical value.	Throughout Discussion
