# Supplementary material for: Interpretable survival prediction for colorectal cancer using deep learning
Source: NPJ Digit Med. 2021 Apr 19;4:71. doi: 10.1038/s41746-021-00427-2 (PMC8055695; doi:10.1038/s41746-021-00427-2)
Supplement: Supplementary file 5 — Reporting Summary [file 41746_2021_427_MOESM5_ESM.pdf]

nature research

Corresponding author(s): Yun Liu
Last updated by author(s): Dec 7, 2020

Reporting Summary

Nature Research wishes to improve the reproducibility of the work that we publish. This form provides structure for consistency and transparency in reporting. For further information on Nature Research policies, see our Editorial Policies and the Editorial Policy Checklist.

Statistics

For all statistical analyses, confirm that the following items are present in the figure legend, table legend, main text, or Methods section.

n/a	Confirmed	
☐	☒	The exact sample size (n) for each experimental group/condition, given as a discrete number and unit of measurement
☐	☒	A statement on whether measurements were taken from distinct samples or whether the same sample was measured repeatedly
☐	☒	The statistical test(s) used AND whether they are one- or two-sided<br>*Only common tests should be described solely by name; describe more complex techniques in the Methods section.*
☐	☒	A description of all covariates tested
☐	☒	A description of any assumptions or corrections, such as tests of normality and adjustment for multiple comparisons
☐	☒	A full description of the statistical parameters including central tendency (e.g. means) or other basic estimates (e.g. regression coefficient) AND variation (e.g. standard deviation) or associated estimates of uncertainty (e.g. confidence intervals)
☐	☒	For null hypothesis testing, the test statistic (e.g. F, t, r) with confidence intervals, effect sizes, degrees of freedom and P value noted<br>*Give P values as exact values whenever suitable.*
☒	☐	For Bayesian analysis, information on the choice of priors and Markov chain Monte Carlo settings
☒	☐	For hierarchical and complex designs, identification of the appropriate level for tests and full reporting of outcomes
☐	☒	Estimates of effect sizes (e.g. Cohen's d, Pearson's r), indicating how they were calculated

Our web collection on statistics for biologists contains articles on many of the points above.

Software and code

Policy information about availability of computer code

Data collection	This study utilized archived formalin-fixed paraffin-embedded, hematoxylin and eosin stained pathology slides from the Institute of Pathology and the BioBank at the Medical University of Graz. Institutional Review Board approval for this retrospective study using de-identified slides was obtained from the Medical University of Graz (Protocol no. 30-184 ex 17/18).
Data analysis	The deep learning framework (TensorFlow) used in this study is available at https://www.tensorflow.org/; the deep learning architecture and statistical analysis code is provided at https://github.com/Google-Health/colorectal_survival and leverages standard depth-wise separable convolutions available from Keras as SeparableConv2D: https://github.com/tensorflow/tensorflow/blob/v2.1.0/tensorflow/python/keras/layers/convolutional.py.

For manuscripts utilizing custom algorithms or software that are central to the research but not yet described in published literature, software must be made available to editors and reviewers. We strongly encourage code deposition in a community repository (e.g. GitHub). See the Nature Research guidelines for submitting code & software for further information.

Data

Policy information about availability of data

All manuscripts must include a data availability statement. This statement should provide the following information, where applicable:

- Accession codes, unique identifiers, or web links for publicly available datasets
- A list of figures that have associated raw data
- A description of any restrictions on data availability

This study utilized archived de-identified pathology slides, clinicopathologic variables, and outcomes from the Institute of Pathology and the BioBank at the Medical University of Graz. Interested researchers should contact K. Z. to inquire about access to Biobank Graz data; requests for non-commercial academic use will be considered and require ethics review prior to access.

Field-specific reporting

Please select the one below that is the best fit for your research. If you are not sure, read the appropriate sections before making your selection.

☒ Life sciences ☐ Behavioural & social sciences ☐ Ecological, evolutionary & environmental sciences

For a reference copy of the document with all sections, see nature.com/documents/nr-reporting-summary-flat.pdf

Life sciences study design

All studies must disclose on these points even when the disclosure is negative.

Sample size	NA; cohorts were defined such that all patients had at least 5 years of followup and the temporal validation cohort spanned 5 years.
Data exclusions	Immunohistochemistry-stained slides and non-colorectal specimens such as lymph node, small intestine, and other tissue types, were excluded. In addition, cases with low tumor content, death within 30 days of surgical resection and secondary tumor resections were also excluded.
Replication	Results were validated and reproduced on a second validation set (representing a different time period relative to the first validation set).
Randomization	Cases for the first cohort were randomly partitioned into train, tune, and test sets.
Blinding	As a retrospective study, all pathologic staging and clinical evaluations and outcomes were conducted before this study began - and thus blinded to this study.

Reporting for specific materials, systems and methods

We require information from authors about some types of materials, experimental systems and methods used in many studies. Here, indicate whether each material, system or method listed is relevant to your study. If you are not sure if a list item applies to your research, read the appropriate section before selecting a response.

Materials & experimental systems

n/a	Involved in the study	
☒	☐	Antibodies
☒	☐	Eukaryotic cell lines
☒	☐	Palaeontology and archaeology
☒	☐	Animals and other organisms
☐	☒	Human research participants
☒	☐	Clinical data
☒	☐	Dual use research of concern

Methods

n/a	Involved in the study	
☒	☐	ChIP-seq
☒	☐	Flow cytometry
☒	☐	MRI-based neuroimaging

Human research participants

Policy information about studies involving human research participants

Population characteristics	Stage II and III colorectal resection cases. Please see Supplementary Table S1 for patient characteristics.
Recruitment	Retrospective, de-identified cohort based on BioBank Graz
Ethics oversight	Medical University of Graz (Protocol no. 30-184 ex 17/18).

Note that full information on the approval of the study protocol must also be provided in the manuscript.
